# Single-cell RNA sequencing and multi-omics analysis of prognosis-related staging in papillary thyroid cancer

**DOI:** 10.1007/s00262-025-04101-4

**Published:** 2025-07-12

**Authors:** Guo Ji, Hanlin Sun, Simo Chen, Xuechen Sun, Le Chang, Ruting Xie, Runzhi Huang, Lijun Zheng, Zhengyan Chang

**Affiliations:** 1 Department of Pathology, Shanghai Tenth People’s Hospital, Tongji University School of Medicine, Middle 301 Yanchang Road, Shanghai, 200072 China; 2 Department of Burn Surgery, the First Affiliated Hospital of Naval Medical University, 168 Changhai Road, Shanghai, 200433 China; 3 Shanghai Jiao Tong University School of Medicine, Shanghai, China; 4 Department of General Surgery, Shanghai Tenth People’s Hospital, Tongji University School of Medicine, Shanghai, China

**Keywords:** Papillary thyroid cancer, Single-cell RNA sequencing, Molecular subtypes, Multi-omics

## Abstract

**Background:**

Papillary thyroid cancer (PTC) is the most common thyroid cancer, but current molecular features inadequately stratify its risk. Whether distinct underlying mechanisms can further classify PTC and improve prognostic precision remains unclear.

**Methods:**

We integrated single-cell RNA sequencing data (158,577 cells from 11 PTC patients; GEO: GSE184362) with bulk-RNA sequencing data from The Cancer Genome Atlas Thyroid Carcinoma (TCGA-THCA) cohort (501 patients). Multi-omics analyses were employed to elucidate PTC heterogeneity, identify malignant cell differentiation and prognosis-related genes (MCD&PRGs), and construct a novel molecular classification, the Oncogenic Signature Of Papillary Thyroid Carcinoma Classification (OSPTCC). A prognostic risk score was developed, and the classification's prognostic relevance was further explored in an independent institutional cohort using qRT-PCR.

**Results:**

Single-cell analysis revealed three malignant cell differentiation states (PTC1-3) and a 34-gene signature (MCD&PRGs). This formed the basis of our Oncogenic Signature Of Papillary Thyroid Carcinoma Classification (OSPTCC), defining three subtypes: Inflammation-associated (IPTCC), BRAF/autophagy-related (BAPTCC), and lipid metabolism-related (LPTCC). These subtypes showed distinct molecular profiles and significantly different progression-free survival (IPTCC poorest, *P* = 0.044). A 7-gene risk score derived from MCD&PRGs independently predicted prognosis (multivariate *HR* = 21.511, *P* < 0.001). qRT-PCR validation in an independent cohort (n = 48) using key markers (DEPTOR, APOE, APOC1) confirmed that OSPTCC-based risk stratification correlated with adverse clinical features, including higher recurrence rates in the high-risk group (*P* = 0.007).

**Conclusions:**

This study introduces OSPTCC, a prognostically significant molecular classification for PTC based on tumor cell differentiation states. The identified subtypes, characterized by distinct biological mechanisms, provide deeper insights into PTC's molecular pathology and offer a framework for improved risk stratification and potential precision therapies.

**Supplementary Information:**

The online version contains supplementary material available at 10.1007/s00262-025-04101-4.

## Introduction

Papillary thyroid cancer (PTC) is a type of differentiated thyroid cancer (DTC), which accounts for about 85% of all thyroid cancer cases [1]. Thyroid cancer (THCA) has attracted global attention with the fastest incidence rate in decades [2, 3]. However, the mortality rate of DTC remains unchanged [4, 5], with a disease-specific mortality rate of less than 5% at 10 years [6, 7]. This phenomenon suggests that although PTC is less malignant, its high incidence still leads to many patients dying or suffering from the disease due to PTC. It shows that different THCA patients' prognosis may differ significantly. Most existing studies on PTC prognosis have focused on clinical features' effect on prognosis [8, 9]. This phenomenon is reflected in the application of clinical risk stratification. The American Joint Committee on Cancer (AJCC), the American Thyroid Association (ATA), and the European Thyroid Association (ETA) are now the most widely used risk classification systems [10–12]. These traditional risk stratification systems are widely used to predict the overall prognosis of patients with THCA. However, THCA risk stratification is not reflected at the molecular genetic level relative to other tumors. Whether differential pathogenic mechanisms exist to further classify or stage PTC has not been addressed. This situation also makes the research of targeted drugs for THCA, especially PTC, still in its infancy [13].

As the most common type of THCA, most PTCs are clinically inert. Due to the low copy number alterations and relatively simple genome, PTC is considered one of the cancers with the lowest density of cancer mutations [14]. Despite the low mutation rate, malignancy, and invasiveness, highly malignant tumor characteristics such as immune escape are still present in advanced PTC. Incidence of aggressive PTC (tumors > 2 cm in diameter) has increased 1.5–fivefold over the past 30 years, with a sustained increase in the incidence of time to recurrence [7]. Therefore, we believe that clinical clustering of PTC based on genetic and molecular characteristics is beneficial for risk re-stratification of PTC patients and more targeted treatment of patients at high risk for events such as metastasis and tumor progression.

In the past studies, adequate progress has been made in Bulk-RNA sequencing (Bulk-RNA-seq) of PTC, and we have gained some understanding of the genomic landscape of PTC [15]. While Bulk-RNA-seq can paint a genetic landscape of the entire tumor entity, it inevitably averages the expression profiles of different cells and obscures key differences between tumor components. Addressing this issue requires further elucidation of the complex tumor microenvironment's components, properties, and underlying mechanisms. The tumor microenvironment (TME) is considered one of the crucial links in tumor development. Through numerous immunosuppressive agents, tumors can damage host immune cells in TME and evade their surveillance [16]. It has been suggested that in PTCs, changes in the immune component of the tumor microenvironment are associated with changes in thyroid differentiation and BRAF^V600E^ mutations [17]. Single-cell RNA sequencing (scRNA-seq) [18] is a new technique that helps us explore the transcriptome diversity of the tumor and stromal microenvironment, improving the precision of analysis by independently sequencing a population of individual cells from the same tissue, which is of great significance for our more profound understanding of the THCA microenvironment.

In this study, we applied scRNA-seq data from 11 PTC tumors obtained from the Gene Expression Omnibus (GEO) databases to identify the differentiation fate trajectories of THCA cells at different time points during tumor formation and to identify tumor cell differentiation-associated genes. We propose a molecular typing of PTC, the Oncogenic Signature Of Papillary Thyroid Carcinoma Classification (OSPTCC), based on the differentiation status of tumor cells to distinguish the oncogenic mechanism. OSPTCC is a molecular typing proposed for the clinical treatment of PTC and is significantly associated with the prognosis of PTC patients, contributing to a better understanding of the molecular pathology of tumor cells in PTC. In addition, we have predicted the critical driver molecules in different subtypes of OSPTCC to provide a theoretical basis for the precise treatment of PTC patients.

## Method

### Data collection

The scRNA-seq data of thyroid tumor cells were obtained from the GSE184362 dataset, which includes the transcriptome of 158,577 cells from 11 patients with paraneoplastic, localized/advanced tumors, initially treated/recurrent lymph nodes, and radioactive iodine (RAI)-refractory distant metastases, covering the entire clinical course of PTC [19]. Patients with or without cytogenetic abnormalities are read as counts in the matrix. Genomic profiles, RNA sequencing profiles, reverse-phase protein array (RPPA) profiles, demographic characteristics, and clinical information profiles such as overall survival were obtained from The Cancer Genome Atlas (TCGA) database (https://Acga-data.nci.nihgov/tega). The TCGA-THCA project comprises data from approximately 507 unique patients. For the present study, following a rigorous quality control process (based on RNA-seq data quality, sample integrity, and availability of comprehensive clinical and molecular data), a cohort of 501 unique patients was selected for inclusion. From these 501 selected patients, a total of 510 tumor tissue samples and 58 adjacent non-tumor tissue samples with corresponding high-quality data were utilized in our analyses. An internal audit of these 501 patients confirmed that 8 cases (1.6%) were annotated by TCGA pathologists with non-PTC histologies (principally follicular or poorly differentiated carcinomas; details in Supplementary Table S1), while the remaining 493 patients were confirmed as PTC or its variants. Moreover, all raw data were downloaded from public databases, and no additional ethical proof was required. All original code is available in supplementary information. Any additional information required to reanalyze the data reported in this paper is available from the corresponding author upon request.

### Data processing

The scRNA-seq data obtained from the GSE184362 dataset were imported into the R environment for quality control. Analysis was performed using the Seurat R package. Only cells expressing more than 1,000 transcripts and with less than 10% of mitochondrial genes were included in further analysis. Moreover, only genes expressed in at least 3 cells were included in further analysis. The data are then normalized using the global scaling normalization method LogNormalize with a scaling factor of 10,000, and the normalized values are stored in the pbmc data. Before using principal component analysis (PCA), the ScaleData function was applied to linearly transform the scaled data to equalize the weights for downstream analysis so that each gene had a mean expression of 0 and a variance of 1 in all cells. The top 1,500 highly variable variants in all genes were identified using the variance stabilizing transformation (VST) method and used for subsequent dimensionality reduction analysis. The variant genes were selected for PCA using the FindVariableFeatures function. The top 20 principal components (PCs) were included in the dimensionality reduction analysis with the help of elbowplot and dimheatmap figures to determine the principal components. The statistically significant PCs were selected for Uniform Manifold Approximation and Projection for Dimension Reduction (UMAP), followed by k-means clustering.

### Cell communication analysis

We performed a cell communication analysis (https://github.com/Coolgenome/iTALK) using the iTALK package (version: 0.1.0) in R language to elucidate significant cell communication patterns between individual cell types of PTC and to identify specific ligand–receptor relationship pairs. The Wilcoxon rank sum test was used to identify cellular communication patterns that differed significantly between time groups. The top 200 cellular communication patterns with the smallest *P*-values were selected. The results were visualized using iTALK network plots and ligand–receptor plots.

### Cell-type annotation

Cells in each cluster were annotated according to differentially expressed genes in each cluster in UMAP clustering and tissue marker genes reported in previous articles. FindAllMarkers of Seurat function were used to identify specific markers for each cluster. Heat maps were used to represent the scaled expression data of these marker genes. The normalized data were expressed as feature maps or violin plots. To determine the biological cell type of each unsupervised cluster, we used the differentially expressed genes (DEGs) of each cluster as potential cell-type marker genes in combination with known cell surface markers retrieved from the CellMarker database for comprehensive cell-type annotation [20].

To assess the distribution of different subpopulations in each cell type (B cells, endothelial cells, endocrine cells, NK/T cells, fibroblasts, myeloid cells, malignant cells) at the temporal and spatial levels, we also extracted each cell type separately and constructed Seurat objects and performed subpopulation-by-subpopulation analysis. Run UMAP, FindAllMarkers function, and Wilcoxon rank sum test were used to identify the DEGs of different cell subpopulations in the eight cell types. Finally, the spatial distribution differences and expression characteristics of the seven cell subpopulations were shown by cell characteristics map, spatial characteristics map, and heat map.

### Analysis of malignant cell clusters and differentiation trajectories

To assess the distribution of different clusters of malignant cells in PTC at different differentiation times, we utilized RunUMAP, the FindAllMarkers function, and the Wilcoxon rank sum test. We constructed Seurat objects for malignant cells from all samples and performed subgroup analysis. Cell feature maps, Cleveland plots, and heat maps showed the expression characteristics of the top 4 or 5 DEGs with the most significant differences between malignant cell subpopulations. Next, malignant cell differentiation trajectory analysis was performed with the help of Monocle package (version 2.18.0) in R language. The differentiation sequence of each malignant cell subtype was determined by fitting the pseudotime of each malignant cell subtype based on the expression profile information. All malignant cells' Unique Molecular Identifiers (UMI) expression matrix was analyzed as the input data by malignant cell differentiation trajectory, and unsupervised sorting of all cells was performed. After that, the DDRTree algorithm is used to downscale the cell clusters, embed the master map into the high-dimensional scRNA-seq data, find the mapping between the high-dimensional gene expression space and the low-dimensional space, construct MST using DDRTree, project the expression data into the low-dimensional space, construct the differentiation trajectory among cells, and sort the cells with MST recursive calculation of pseudotimes. Subsequently, the hypothesis of cell differentiation fate was obtained by applying the branch expression analysis model, BEAM, to infer cell differentiation trajectories and compare the differences between branch points and branch ends [21]. Finally, we also calculated DEGs between malignant cell subtypes at different stages of differentiation (State) and defined them as Malignant cell Differentiation Genes (MDGs). MDGs were selected based on *P* < 0.05 obtained by the DDRTree algorithm and genes used to rank differentiation trajectories.

### Identification of malignant cell differentiation & prognosis-related genes (MCD&PRGs) and construction of OSPTCC subtypes

To identify a gene signature reflecting both malignant cell differentiation state and clinical prognosis, we first processed scRNA-seq data (GSE184362), performing quality control, cell-type annotation, and identification of malignant subpopulations. Pseudotime trajectory analysis was conducted on the malignant cells using Monocle2. Genes significantly associated with the differentiation trajectory were identified based on the DDRTree algorithm (q-value < 0.05) and their use in trajectory ordering; these were initially defined as MDGs. To refine this list for robust prognostic relevance and applicability to bulk tumors, we integrated these MDGs with bulk-RNA-seq data and clinical outcomes (progression-free survival, PFS) from 510 PTC patients in the TCGA-THCA cohort. We applied stringent filtering, retaining only those genes that simultaneously satisfied all four of the following criteria: (1) Identified as an MDG based on the DDRTree algorithm (q-value < 0.05) from the single-cell pseudotime analysis. (2) Exhibited a significant correlation between gene expression and pseudotime progression in the single-cell data (nonparametric test *P* < 0.05). (3) Demonstrated significant prognostic association in the TCGA cohort via Kaplan–Meier (KM) survival analysis (nonparametric test *P* < 0.05). (4) Showed significant prognostic association in the TCGA cohort via univariate Cox regression analysis (nonparametric test *P* < 0.05). This rigorous multi-step filtering process, integrating both single-cell differentiation characteristics and bulk tumor prognostic features, yielded the final core set of 34 genes termed malignant cell differentiation & prognosis-related genes (MCD&PRGs), which formed the basis for constructing the OSPTCC subtypes.

Subsequently, the OSPTCC molecular subtypes were constructed based on the expression profiles of these 34 MCD&PRGs within the TCGA-THCA bulk tumor dataset (n = 510). We employed the ConsensusClusterPlus R package, utilizing a k-means-based consensus clustering algorithm (CCA). This algorithm groups samples exhibiting similar MCD&PRG expression patterns, effectively partitioning samples based on shared molecular characteristics reflected by Euclidean distance. The stability and reliability of the clustering results were systematically evaluated across different potential cluster numbers (k), assessing quantitative and visual evidence (e.g., Cumulative Distribution Function delta area plots) to determine the optimal k value that best partitions the data into robust and distinct subtypes [22]. This process established the final OSPTCC classification structure presented in our study.

### Principal component analysis and clinical correlation analysis

Following the identification of the three OSPTCC molecular subtypes (Cluster 1–3) based on consensus clustering of the 34 MCD&PRGs, we sought to derive a continuous measure representing the underlying biological signature and to facilitate a potentially more practical binary risk stratification. To achieve this, we applied principal component analysis (PCA) to the expression data of the 34 MCD&PRGs across the TCGA-THCA samples. The score from the first principal component (PC1), which captures the largest variance in the MCD&PRG expression data, was calculated for each sample (referred to as the PCA score or PCA_THCA_score). This quantitative PCA score summarizes the dominant expression pattern of the MCD&PRG signature, transforming the categorical subtype information into a continuous variable. This continuous score was then used for several purposes: 1) To potentially reduce bias inherent in analyzing purely categorical data; 2) to enable quantitative correlation analyses between the MCD&PRG signature and other continuous multi-omics data types (e.g., tumor mutation load, immune infiltration scores); and 3) to establish a simplified binary risk stratification (high risk vs. low risk) by dichotomizing patients based on the median PCA score, aiming to capture the primary prognostic signal while enhancing potential clinical utility.

To evaluate the clinical relevance of both the categorical OSPTCC subtypes and the continuous PCA score, we performed comprehensive clinical correlation analyses. We assessed the association of both the OSPTCC clusters (Cluster 1 vs. 2 vs. 3) and the dichotomized PCA score (high risk vs. low risk) with patient prognosis, primarily using PFS as the clinical endpoint. Kaplan–Meier survival analysis and Cox proportional hazards regression (with adjusted *P*-values where appropriate) were employed. Furthermore, the relationships between the OSPTCC subtypes, the continuous PCA score, and various clinical/molecular features (e.g., copy number variation, specific gene mutations like BRAF/RAS, tumor mutation burden, microsatellite instability, predicted immunotherapy response markers, immune cell infiltration) were investigated using appropriate statistical tests (e.g., ANOVA, t tests, Chi-squared tests, correlation analyses). Results were visualized using box plots, bar plots, violin plots, heatmaps, and survival curves, with *P* < 0.05 generally considered statistically significant. This integrated analysis aimed to confirm the prognostic significance of the OSPTCC framework and explore the underlying biological and clinical correlates of the identified molecular states.

### Predictive model construction, test, and diagnosis

To develop and validate a prognostic signature based on the identified MCD&PRGs, we utilized the expression profiles of these genes and corresponding clinical survival data (PFS) from the TCGA-THCA cohort. Initially, univariate Cox proportional hazards regression analysis was specifically performed on the 34 MCD&PRGs to identify those independently associated with PFS (*P* < 0.05). Subsequently, the subset of MCD&PRGs found significant in the univariate analysis was subjected to Lasso (Least Absolute Shrinkage and Selection Operator) Cox regression algorithm. This machine learning step performed penalized variable selection to identify the most robust prognostic markers. Using tenfold cross-validation, the optimal tuning parameter (lambda) was determined based on minimum partial likelihood deviance. Finally, a multivariate Cox proportional hazards model was built using the final set of genes retained after Lasso selection. The regression coefficients from this multivariate model were used to calculate a risk score for each patient. Patients were then stratified into high-risk and low-risk groups based on the median risk score. To evaluate the model's performance rigorously, the TCGA cohort was randomly divided into training (70%) and test (30%) sets. The model's ability to stratify patients was assessed in both sets and the overall cohort using Kaplan–Meier survival curves. Performance visualization included risk curves, survival status scatter plots, and expression heatmaps of the signature genes. Time-dependent receiver operating characteristic (ROC) curves were generated, and the area under the curve (AUC) was calculated to assess the model's discriminative accuracy over time. Finally, to evaluate the independent prognostic value of the derived risk score, both univariate and multivariate Cox regression analyses were performed, incorporating the risk score along with standard clinical factors such as age, sex, and TNM stage.

### Transcription factor analysis

We obtained publicly available chromatin immunoprecipitation sequencing (ChIP-seq) data for 318 transcription factors (TFs) from the Cistrome database (http://cistrome.org/) to detect important TFs in the regulatory network [23]. And we also identified differentially expressed transcription factors (DETFs) between cancer tissue and normal paracancerous tissue. To visualize the results of all DEG analyses, we generated both heat maps and volcano maps.

### Functional enrichment analysis

We used the clusterProfiler package in R (version: 3.18.0)[24] and the Metascape database (https:/metascape.org/gp/index.html) [25] for functional analysis of the DEGs from each cell type. The databases selected for this purpose included the Gene Ontology (GO) database (http://www.geneontology.org) [26] and the Kyoto Encyclopedia of Genes and Genomes (KEGG) database [27]. GO enrichment analysis is used to explore potential biological processes and functions associated with DEGs, including biological processes (BPs), cellular components (CCs), and molecular functions (MFs). In contrast, KEGG enrichment analysis explores the relevant signaling pathways. The screening threshold for significantly enriched GO and KEGG entries was FDR < 0.05. In addition, we used Overall Representation Analysis (ORA) to assess the proportion of DEGs to known gene sets. All known gene sets in this study were obtained from the Molecular SignaturesDatabase (MSigDB) (version: 7.1) (https:/github.com/tomastokar/gsoap) [28] and can be classified into 9 categories of gene sets based on different functional characteristics, including C1-C8 gene sets and hallmark gene set. In addition, we used GSVA to quantify specific gene sets for neuroregeneration and inflammation, which was done by the GSVA package in R (version: 1.38.0) [29].

### Signaling pathway analysis

We quantified 50 Hallmark signaling pathways across 7 clusters of malignant cells by GSVA algorithm, and the gene set files for these 50 Hallmark signaling pathways were sourced from the MSigDB database (version: 7.1) [28]. Following the completion of the quantitative analysis, the results were visualized by heat map using the limma software package in R language. Since the GSVA algorithm normalizes the output results to the range [−1,1], the screening criterion for differential pathways was set to FDR < 0.05.

### Analysis of immune cell infiltration patterns

As a method for estimating the cellular composition of complex tissues based on gene expression profile characterization, cell-type identification by estimating relative subsets of RNA transcripts (CIBERSORT) is commonly used to explore the composition of infiltrating immune cells. In this study, to determine the association between identified MDGs and immune cell infiltration in PTC tissues, we first uploaded microarray expression profile data and RNA-seq gene expression matrix data to the CIBERSORT database (https://cibersort.stanford.edu) to obtain the percentage of 22 purified immune cell types in each sample, and only samples with *P* < 0.05 were considered accurate estimates and could be included in the subsequent analysis [30]. In addition, to reduce the bias generated by a single algorithm, we also used single sample gene set enrichment analysis (ssGSEA) algorithm to evaluate and quantify 23 immune cells and immune functions in PTC tissues [31].

### Construction of regulatory network for each subtype of OSPTCC

To further investigate the potential key regulatory mechanisms of each subtype of OSPTCC, we conducted co-expression analysis. The Pearson/Spearman correlation analysis was selected based on the normal distribution of data and the Chi-square test. This analysis was applied to the quantitative results of MCD&PRGs for each subtype of OSPTCC, immune cells/immune functions, and signaling pathways. We established a minimum screening criterion of a correlation coefficient (R) greater than 0.300 and a *P*-value less than 0.001 to construct a complex regulatory network. This network was centered around the MCD&PRGs of the OSPTCC subtypes as the regulatory core and included upstream transcription factors (TFs), downstream signaling pathways, and potential regulation of immune cells/functions. The regulatory network was visualized using the igraph package in R language. (http://igraph.sourceforge.net) [32].

### Prediction of small molecule active drugs targeted by each subtype of OSPTCC

The Connectivity Map (Cmap) is an algorithm to study the statistical matching relationship between small molecule active drugs and their target genes. This is achieved by utilizing genome-wide transcriptional expression data from human cells treated with these small molecule active drugs. The Cmap algorithm incorporates more than 7,000 cell culture systems and 1,309 small molecule active drugs, all of which are FDA-approved. It creates a network that connects drugs, functional genes, and diseases, providing a statistical basis for identifying small molecule inhibitors related to target genes for the treatment of associated diseases (https://portals.broadinstitute.org/cmap/) [33]. The Cmap algorithm was used in this study to explore small molecule drugs targeting the regulatory network for each OSPTCC subtype. Finally, we screened the 10 drugs with the smallest *P*-values for visualization using dotted heat maps as the target drugs for each OSPTCC subtype, and subsequent analysis combined with typed gene expression profiles to select the best active drug. Furthermore, we utilized genomic data from the Genomics of Drug Sensitivity in Cancer (GDSC) for predictive modeling of pharmacological susceptibility. This involved applying information on eight common thyroid cancer cell lines, integrating pharmacological data with genomic information, and visualizing the results as heat maps and box plots [34].

### Patient collection

To further verify the clinical classification proposed by this study, we collected clinical patient samples. This study was approved by the Institutional Review Board for Clinical Research of Shanghai Tenth People’s Hospital affiliated with Tongji University. Samples were collected at Shanghai Tenth People’s Hospital affiliated with Tongji University from 2019 to 2022. The cohort selection adhered to stringent criteria: Inclusion required histopathological confirmation of PTC via surgical resection, age ≥ 18 years to ensure physiological relevance and ethical compliance, treatment-naïve status for newly diagnosed PTC to eliminate prior treatment interference, complete clinical documentation including preoperative imaging, laboratory results, and follow-up records, standardized surgical intervention (total/partial thyroidectomy), and exclusion eliminated non-PTC malignancies (e.g., follicular/medullary/anaplastic carcinoma), concurrent malignancies or autoimmune comorbidities (e.g., Hashimoto's thyroiditis), pregnancy or lactation, informed consent deficiencies, insufficient preserved tissue, non-compliance with long-term monitoring, or psychiatric conditions affecting protocol adherence.

To ensure diagnostic accuracy and consistency, the following steps were also implemented in terms of pathological re-evaluation: (1) Diagnosis was confirmed through a dual independent review process conducted by two pathologists who remained blinded to clinical outcomes. (2) All cases underwent reclassification aligned with the 2022 WHO Classification of Thyroid Tumors and AJCC Eighth Edition staging protocols to maintain contemporary diagnostic standards. Stringent quality control protocols were systematically implemented, comprising three principal measures: (1) Sample stability: Exclusion of specimens subjected to more than 3 freeze–thaw cycles was excluded to ensure sample stability. (2) Continuous storage verification at − 80 °C was maintained for cryo-preserved specimens. (3) All cases were reconfirmed by Multidisciplinary Tumor Board (MDT).

### Experimental validation cohort stratification for PCR analysis

For the experimental validation using quantitative real-time PCR (qRT-PCR), an initial cohort of 76 PTC patients, meeting the aforementioned inclusion criteria, was assessed. Expression levels of four characteristic genes (APOE, APOC1, DEPTOR, and SQSTM1), representing key biological themes of the OSPTCC subtypes, were measured. For the purpose of validating prognostic stratification based on these markers, patients were categorized into high-risk and low-risk groups using a stringent expression profile definition based on DEPTOR, APOE, and APOC1. Specifically: (1) The median expression level for DEPTOR, APOE, and APOC1 was calculated across all 76 samples. (2) The high-risk group was defined as patients whose tumor samples exhibited DEPTOR expression above its median AND (APOE expression below its median OR APOC1 expression below its median). (3) The low-risk group was defined as patients whose tumor samples exhibited DEPTOR expression below its median AND (APOE expression above its median OR APOC1 expression above its median). SQSTM1 expression, while measured, was primarily considered a marker for the BAPTCC (intermediate) subtype and was not used for this specific dichotomous high-/low-risk stratification. Patients whose expression profiles for DEPTOR, APOE, and APOC1 did not simultaneously meet the criteria for either the high-risk or low-risk group were classified into an"other expression pattern"category and were excluded from the subsequent comparative analysis of clinical features presented in Table 1 and Fig. 7. This re-stratification resulted in a final set of 48 patients (25 high risk, 23 low risk) for this validation analysis. Raw clinical data for all 76 initially assessed patients are provided in Supplementary Material (Table S2-S3).

**Table 1:** Differences in clinical prognostic features among 48 PTC patients categorized into high- and low-risk groups

Variables	Total (n = 48)	High risk (n = 25)	Low risk (n = 23)	Statistic	*P*
Age, Mean ± SD (yrs)	46.94 ± 12.82	51.08 ± 12.63	42.43 ± 11.67	t = 2.47	**0.017**
Size, Mean ± SD (cm)	2.18 ± 2.20	2.79 ± 2.70	1.51 ± 1.22	t = 2.15	**0.039**
Lymph node metastasis rate, Mean ± SD	0.45 ± 0.36	0.49 ± 0.37	0.41 ± 0.34	t = 0.71	0.483
Gender, n (%)				χ^2^ = 0.68	0.410
Female	29 (60.42)	17 (68.00)	12 (52.17)		
Male	19 (39.58)	8 (32.00)	11 (47.83)		
Multiple foci, n (%)				χ^2^ = 0.05	0.815
No	29 (60.42)	16 (64.00)	13 (56.52)		
Yes	19 (39.58)	9 (36.00)	10 (43.48)		
Thyroiditis, n (%)				χ^2^ = 0.27	0.602
No	30 (62.50)	17 (68.00)	13 (56.52)		
Yes	18 (37.50)	8 (32.00)	10 (43.48)		
Thyroid nodules, n (%)				χ^2^ = 0.31	0.578
No	22 (45.83)	10 (40.00)	12 (52.17)		
Yes	26 (54.17)	15 (60.00)	11 (47.83)		
KRAS mutations, n (%)				–	1.000
No	48 (100.00)	25 (100.00)	23 (100.00)		
Yes	0 (0.00)	0 (0.00)	0 (0.00)		
BRAF mutations, n (%)				χ^2^ = 0.00	1.000
No	5 (10.42)	3 (12.00)	2 (8.70)		
Yes	43 (89.58)	22 (88.00)	21 (91.30)		
TERT mutations, n (%)				χ^2^ = 0.00	1.000
No	46 (95.83)	24 (96.00)	22 (95.65)		
Yes	2 (4.17)	1 (4.00)	1 (4.35)		
Recurrent, n (%)				χ^2^ = 7.40	**0.007**
No	29 (60.42)	10 (40.00)	19 (82.61)		
Yes	19 (39.58)	15 (60.00)	4 (17.39)		
TPO, n (%)				χ^2^ = 0.00	1.000
Negative	46 (95.83)	24 (96.00)	22 (95.65)		
Positive	2 (4.17)	1 (4.00)	1 (4.35)		
HBME-1, n (%)				χ^2^ = 0.02	0.875
Negative	11 (22.92)	5 (20.00)	6 (26.09)		
Positive	37 (77.08)	20 (80.00)	17 (73.91)		
CD56, n (%)				χ^2^ = 0.00	1.000
Negative	45 (93.75)	23 (92.00)	22 (95.65)		
Positive	3 (6.25)	2 (8.00)	1 (4.35)		

t: t test, χ^2^: Chi-square test, -: Fisher exact

*SD* standard deviation

### RNA extraction and quantitative real-time PCR

TRIzol reagent (Invitrogen) was utilized to isolate total RNA following the manufacturer’s instructions. Briefly, the OD260/OD280 ratio ranged from 1.8 to 2.0. M-MLV reverse transcriptase (Takara, Japan), and 500 ng of RNA was added to a 10 µl reaction volume for reverse transcription. Quantitative real-time PCR was conducted using the QuantStudio DX Detection System with the SYBR Premix ExTaq (Takara, Japan). The reaction process included an initial denaturation step for 3 min at 95℃, followed by a proliferation step including 40 cycles of PCR (95℃ for 5 s, 60℃ for 34 s). The final dissociation stage started at 60℃, with 0.5℃ increments every 15 s up to 95℃. Following PCR completion, the cycle threshold (CT) data and the mean CT were determined using the fixed threshold settings and triplicate PCRs. To compare each condition to the control reactions, the comparative CT method was performed. Furthermore, mRNA levels and the relative amount of the gene were normalized to β-actin and control, respectively. The primers for APOC1,APOE,DEPTOR,SQSTM1, and β-actin are listed in Supplementary Table S4.

### Statistical analysis

All statistical analysis was performed using R version 4.0.3 (Institute for Statistics and Mathematics, Vienna, Austria; www.r-project.org). Except where noted, two-tailed *P* < 0.05 was regarded statistically significant.

## Result

### scRNA-seq analysis reveals cellular heterogeneity and microenvironmental landscape in PTC

**Fig. 1:**
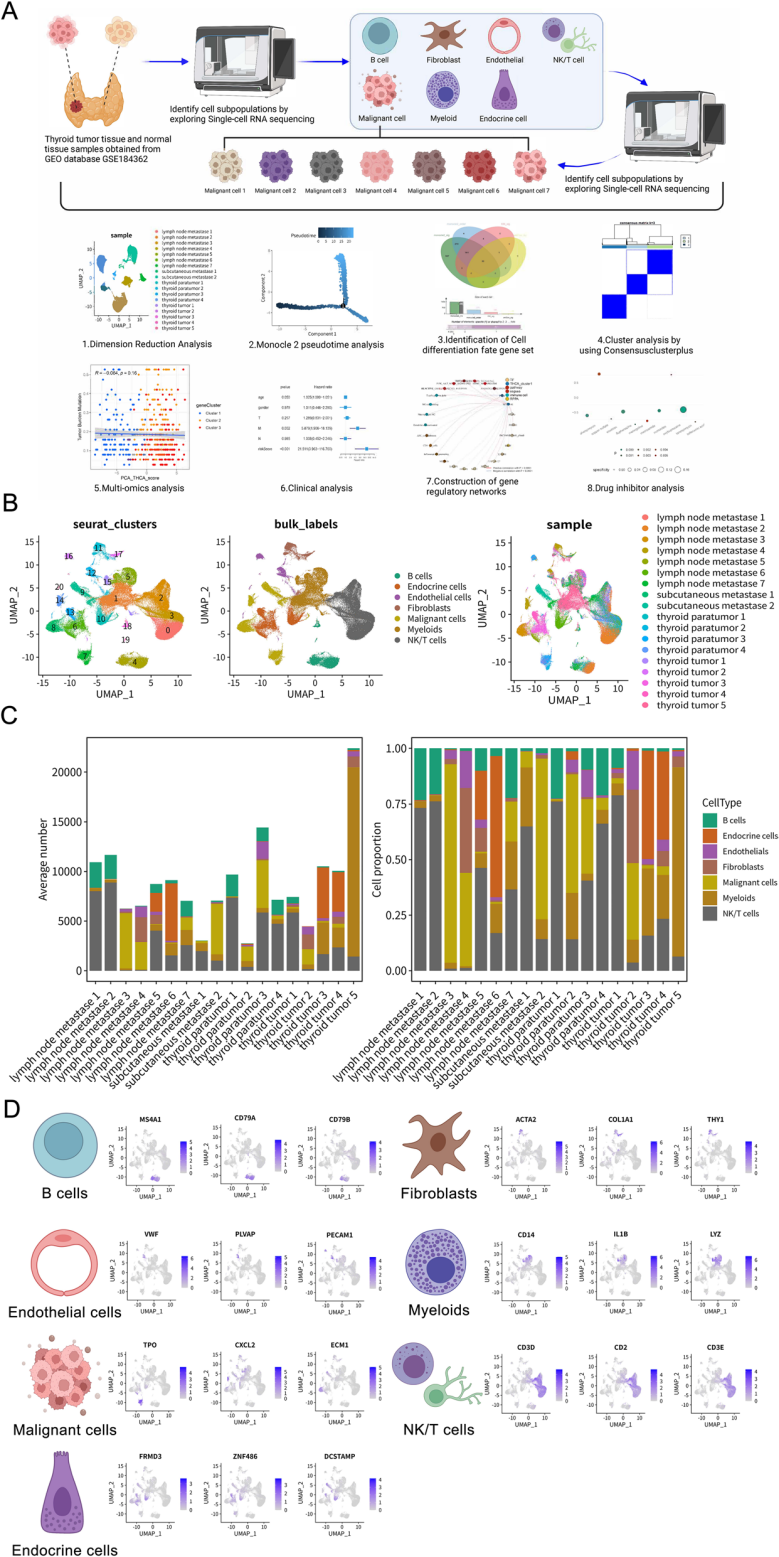
Single-cell RNA sequencing reveals cellular heterogeneity in papillary thyroid cancer. **A** Schematic overview of the study workflow, including data acquisition from GSE184362, scRNA-seq analysis steps (dimensionality reduction, pseudotime analysis, gene set identification, consensus clustering), multi-omics integration, clinical analysis, regulatory network construction, and drug prediction. **B** Uniform Manifold Approximation and Projection (UMAP) plots of 158,577 cells. Left: Cells colored by unsupervised Seurat clusters (0–19). Middle: Cells colored by annotated cell types (B cells, endocrine cells, endothelial cells, fibroblasts, malignant cells, myeloids, NK/T cells). Right: Cells colored by sample origin (lymph node metastasis, subcutaneous metastasis, thyroid paratumor, thyroid tumor). **C** Bar plots showing the average absolute number (left y-axis) and relative proportion (right y-axis, stacked bars) of each annotated cell type across different tissue samples. **D** UMAP feature plots illustrating the expression levels of representative canonical marker genes used for annotating major cell types: B cells (MS4A1, CD79A, CD79B), fibroblasts (ACTA2, COL1A1, THY1), endothelial cells (VWF, PLVAP, PECAM1), myeloids (CD14, IL1B, LYZ), malignant cells (TPO, CXCL2, ECM1), endocrine cells (FRMD3, ZNF486, DCSTAMP), and NK/T cells (CD3D, CD2, CD3E). Color intensity indicates expression level

To characterize the cellular landscape of PTC, we analyzed scRNA-seq data from the GSE184362 dataset (11 patients; 158,577 cells), including thyroid tumors, paratumor tissues, and metastases (lymph nodes and subcutaneous) (Table S5). The experimental workflow is detailed in Fig. 1A and Figure S1. Unsupervised clustering using UMAP identified 20 distinct clusters, which were annotated into seven major cell types: malignant cells, fibroblasts, myeloid cells, endothelial cells, endocrine cells, NK/T cells, and B cells (Fig. 1B). UMAP visualization demonstrated spatial segregation between parenchymal cells (e.g., malignant/endocrine) and immune cells (e.g., NK/T/B cells), with malignant cells forming a large, diffuse cluster, indicative of intratumor heterogeneity. Cells predominantly clustered by tissue origin (tumor vs. paratumor), suggesting tissue-specific microenvironmental influences, and cross-sample contributions to cell-type annotation confirmed minimal batch effects after quality control.

Quantitative analysis of cell composition across tissue types revealed distinct TME profiles (Fig. 1C). Primary thyroid tumors ("thyroid tumors 1–5") exhibited the highest average proportion of malignant cells among all samples. In contrast, paratumor tissues ("thyroid paratumor 1–4") were dominated by NK/T cells and fibroblasts, with minimal malignant cell infiltration. Metastatic lesions—including lymph nodes ("lymph node metastases 1–7") and subcutaneous sites ("subcutaneous metastases 1–2")—showed substantially elevated myeloid and NK/T cell proportions compared to primary tumors, while malignant cell percentages varied widely in metastases. This immune-enriched shift in cellular composition suggests profound TME remodeling during PTC metastasis.

To validate the accuracy of cell-type annotation, we examined the expression patterns of established marker genes (Fig. 1D). The analysis confirmed specific marker expression patterns: MS4A1, CD79A, and CD79B in B cells; ACTA2, COL1A1, and THY1 in fibroblasts; CD14, IL1B, and LYZ in myeloid cells; VWF, PLVAP, and PECAM1 in endothelial cells; TPO, CXCL2, and ECM1 in malignant thyroid cells; FRMD3, ZNF486, and DCSTAMP in endocrine cells; and CD3D, CD2, and CD3E in NK/T cells. The distinct, cluster-specific expression of these markers robustly validated our cell-type identification.

To further validate the identified cell types and explore the broader TME interactions, we examined specific gene expression patterns and predicted intercellular communication networks (Figure S2). The specific expression of selected canonical markers was confirmed across the major cell types using a Cleveland dot plot (e.g., TG in malignant cells, C1QB in myeloids, CD3D in NK/T cells, COL1A1 in fibroblasts) (Figure S2A, top panel). Analysis of sample composition within each major cell type reinforced the observation of distinct TME makeups, showing varying contributions from primary, paratumor, and metastatic samples to each lineage (Figure S2A, bottom panel). A broader heatmap of top differentially expressed genes provided robust molecular signatures for each of the seven main cell types, further confirming their identities (Figure S2B).

Beyond cell-type markers, we investigated the communication landscape. An overall cell communication network analysis predicted extensive signaling interactions between the seven major cell types within the PTC TME (Figure S2C). Malignant cells, myeloid cells, and fibroblasts appeared centrally involved in this network. The Circos plot visualized specific predicted ligand–receptor pairs mediating these interactions across the entire TME, highlighting molecules like FN1, GNAS, CCL5, and various collagens/integrins as potentially key mediators of cross-talk between malignant cells and the diverse stromal and immune components (Figure S2D).

### Identification and functional characterization of heterogeneous malignant cell subpopulations

**Fig. 2:**
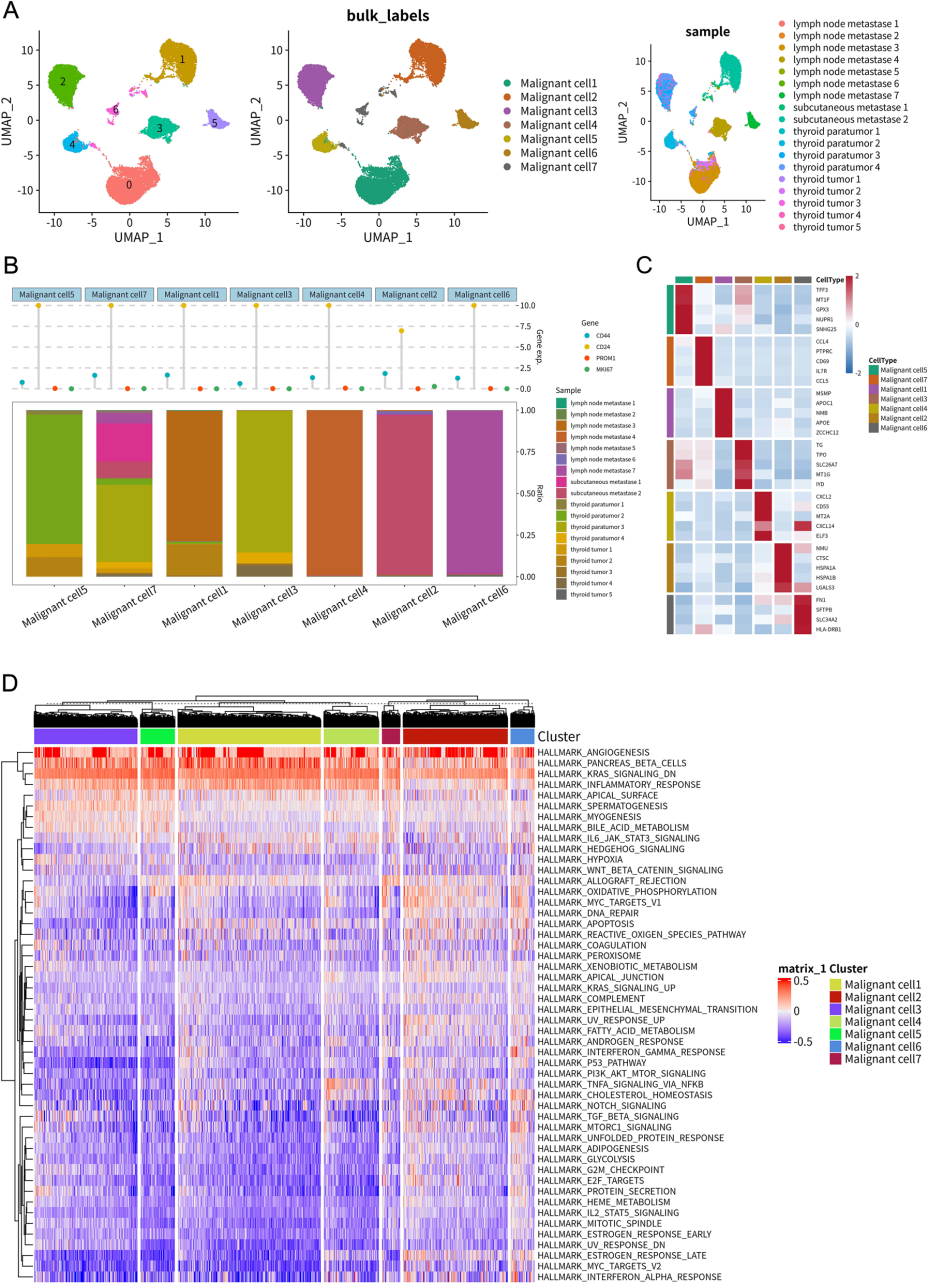
Heterogeneity and functional characteristics of malignant cell subtypes in PTC. **A** UMAP plots focusing on 24,096 malignant cells. Left: Cells colored by malignant subtype annotation (malignant cell 1–7). Middle: Same as left, showing subtype labels. Right: Malignant cells colored by sample origin. **B** Top: Cleveland dot plot showing scaled expression levels of selected cancer-related markers (CD44, CD24, PROM1, MKI67) across the seven malignant subtypes. Bottom: Stacked bar plot illustrating the proportion of cells contributed by each sample origin within each malignant subtype. **C** Heatmap displaying the scaled expression of top differentially expressed genes (DEGs) characterizing each malignant cell subtype. Rows represent genes and columns represent subtypes. Red indicates higher expression, and blue indicates lower expression. **D** Heatmap showing Gene Set Variation Analysis (GSVA) scores for 50 Hallmark pathways across the seven malignant cell subtypes. Rows represent pathways; columns represent cell clusters corresponding to subtypes. Red indicates higher pathway activity; blue indicates lower activity. Hierarchical clustering is applied to pathways (rows)

To investigate malignant cells heterogeneity, we conducted subclustering analysis of 24,096 malignant cells, identifying seven distinct subtypes (malignant cells 1–7) that showed clear separation in UMAP visualization (Fig. 2A). Sample origin analysis revealed distinct distribution patterns: Subtypes 3 and 4 were primarily derived from primary tumors, while subtypes 1, 2, 5, 6, and 7 were significantly enriched in metastatic sites (lymph nodes and subcutaneous) (Fig. 2A-2B). This tissue-specific enrichment pattern suggests that metastasis-associated subtypes, particularly subtype 1, may exhibit enhanced metastatic capacity or represent adaptations to metastatic microenvironments.

Examination of key cancer-associated markers revealed distinct subtype-specific expression patterns (Fig. 2B), with the proliferation marker MKI67 showing higher expression in malignant subtypes 2 and 6, while the potential stem cell marker CD44 was elevated in subtype 3. DEG profiles further emphasized unique molecular identities across subtypes (Fig. 2C). Notably, subtype 1 exhibited high expression of extracellular matrix interaction and invasion-related genes (FN1, TGFBI), subtype 3 showed elevated ALCAM and CD44 expression, and subtype 4 demonstrated distinctive lipid metabolism features characterized by APOE and APOC1 expression. These findings collectively highlight the functional specialization among malignant cell subpopulations.

Cell communication analysis revealed a complex signaling network between malignant subtypes (Figure S2E). Malignant cells 1, 2, and 5 emerged as potential signaling hubs. The frequent involvement of specific ligand–receptor pairs, particularly those interactions involving FN1 and integrins (e.g., ITGA2, ITGA3, ITGB1, ITGB7), suggested an important role for cell–matrix/cell–cell adhesion signaling (Figure S2F). GSVA of hallmark pathways revealed distinct functional profiles across subtypes (Fig. 2D). Metastasis-enriched subtypes 1, 2, 5, 6, and 7 exhibited significantly elevated activity in pathways linked to aggressive tumor behavior, including epithelial–mesenchymal transition (EMT), angiogenesis, hypoxia, and KRAS signaling, with subtype 1 showing particularly strong activation of these aggressive phenotypes. In contrast, subtype 3 displayed predominant activation of inflammatory pathways (inflammatory response, IL6-JAK-STAT3 signaling), while subtype 4 was characterized by enrichment of metabolic pathways. These findings collectively demonstrate the functional diversity among malignant cell subpopulations and their potential contributions to distinct aspects of tumor biology.

### Inferring malignant cell differentiation trajectories and defining molecular subtypes with prognostic relevance

**Fig. 3:**
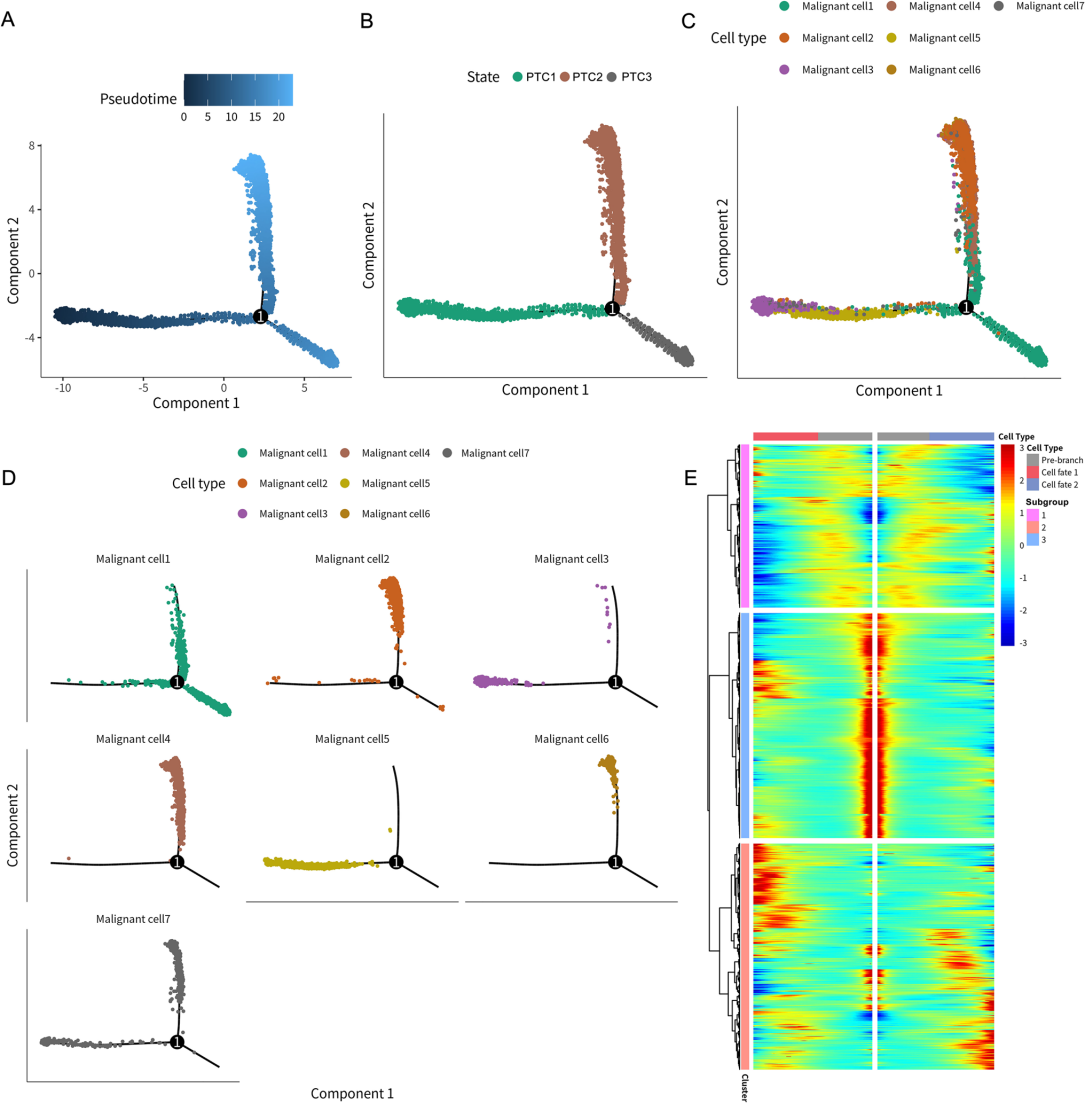
Pseudotime trajectory analysis of malignant cell differentiation. **A** Pseudotime trajectory of malignant cells inferred by Monocle2. Cells are colored according to their calculated pseudotime value (dark blue = early, light blue/yellow = late). The black line represents the inferred trajectory path. **B** The same trajectory plot with cells colored by the three identified principal states (State 1, State 2, State 3) along the differentiation path. The branch point is labeled'1.'**C** The trajectory plot with cells colored by their malignant cell subtype annotation (malignant cell 1–7). **D** Individual trajectory plots faceted by malignant cell subtype, illustrating the distribution of each subtype along the inferred differentiation path. **E** Heatmap showing smoothed gene expression dynamics across pseudotime based on branch point 1 of the pseudotime trajectory. Rows represent trajectory-dependent genes grouped into three clusters (Subgroup 1–3). Columns represent cells ordered by pseudotime, segmented into pre-branch (PTC1) and two differentiation branches (cell fate 1 and cell fate 2, corresponding to PTC2 and PTC3). The distinct expression patterns validate the effectiveness of pseudotime trajectory analysis in capturing transcriptional differences associated with cell fate divergence. Red indicates higher expression, and blue indicates lower expression relative to the average expression across pseudotime

To elucidate the developmental origins of this heterogeneity, we conducted temporal trajectory analysis using Monocle2 (Fig. 3). The analysis reconstructed a branched developmental trajectory originating from an early state (PTC1) that diverged into two terminal fates (PTC2 and PTC3) (Fig. 3A-B). Subtype mapping revealed that primary tumor-associated subtypes 3 and 5 predominantly localized to the trajectory root (PTC1), while the aggressive, metastasis-enriched subtype 1 was primarily positioned at the terminus of the PTC3 branch (Fig. 3C-D). Other metastasis-associated subtypes (2, 4, 6) defined the endpoints of PTC2, with subtype 7 also mapping to PTC3. This spatial organization clearly associates discrete malignant subtypes with specific positions along the differentiation continuum, particularly linking the aggressive subtype 1 with the PTC3 fate. The heatmap in Fig. 3E depicts gene expression dynamics based on branch point 1 in the pseudotime trajectory analysis. It clearly illustrates distinct gene expression patterns across the pre-branch state (PTC1) and the two subsequent differentiation branches (cell fate 1 and cell fate 2, corresponding to PTC2 and PTC3, respectively). The markedly different expression profiles observed before and after branching validate the effectiveness of pseudotime trajectory analysis in identifying key transcriptional changes associated with cell differentiation trajectories.

**Fig. 4:**
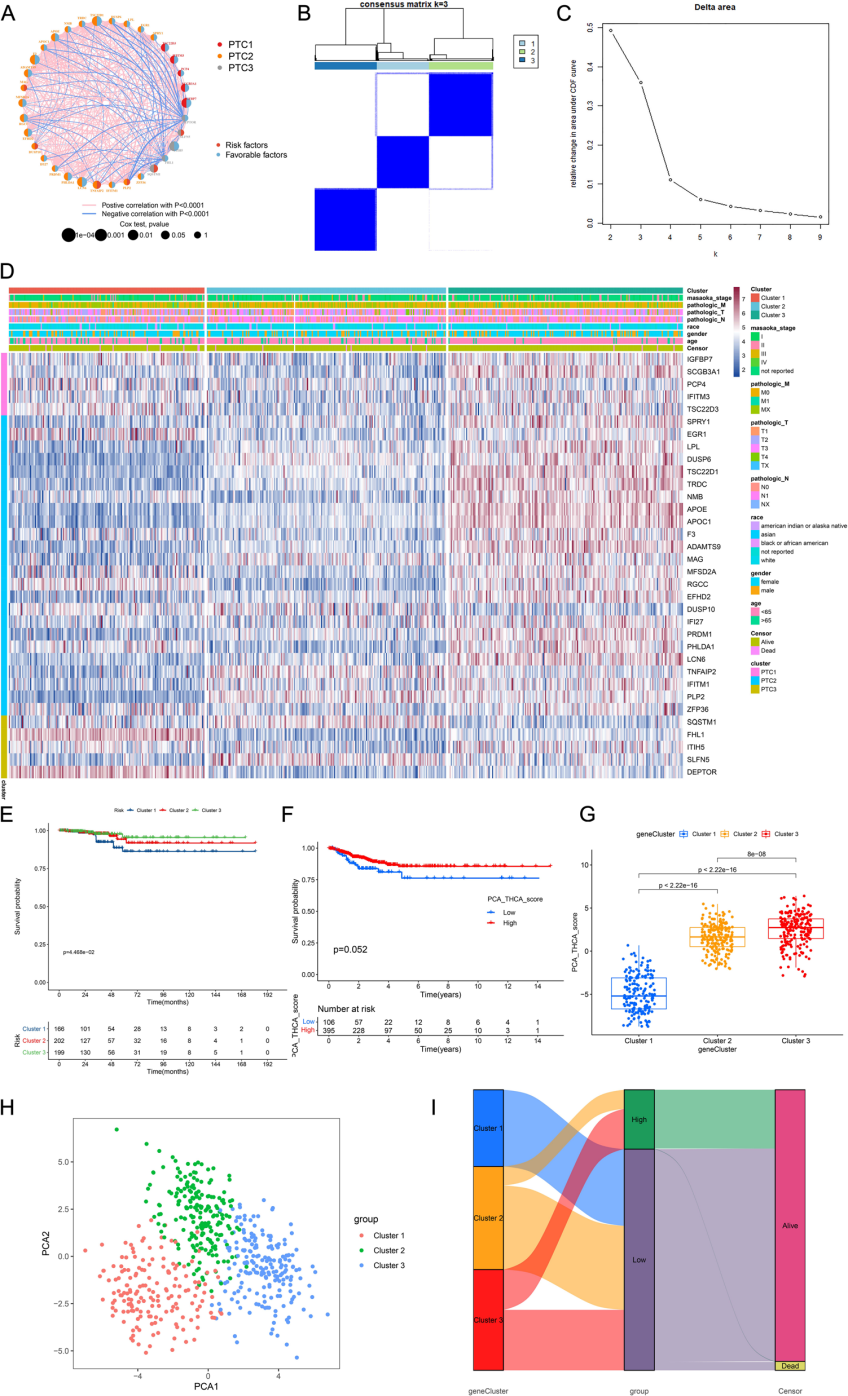
Construction and prognostic significance of OSPTCC molecular subtypes. **A** Co-expression network of the 34 MCD&PRGs. Nodes are colored based on association with differentiation states (PTC1, PTC2, PTC3 from Fig. 3) and prognostic impact (risk vs. favorable factors based on Cox analysis). Edges represent significant correlations (pink = positive, blue = negative, *P* < 0.0001). Node size reflects Cox test *P*-value significance. **B** Consensus matrix heatmap for k = 3 clusters based on MCD&PRG expression in TCGA-THCA. Dark blue indicates high consensus (samples frequently cluster together). **C** Delta area plot showing the relative change in area under the CDF curve for different numbers of clusters (k), indicating optimal stability at k = 3. **D** Heatmap showing the expression profiles of the 34 MCD&PRGs (rows) across TCGA-THCA tumor samples (columns), ordered by OSPTCC cluster assignment (Cluster 1, 2, 3). Clinical annotations (e.g., stage, TNM, gender, age, survival status) are shown above the heatmap. Red indicates higher expression; blue indicates lower expression. **E** Kaplan–Meier curves comparing PFS among the three OSPTCC subtypes (Clusters 1, 2, 3) in the TCGA-THCA cohort. Log-rank *P*-value is shown. **F** Kaplan–Meier curves comparing survival (likely Overall Survival or similar, based on timescale) between patients stratified by the median PCA_THCA_score (low vs. high). Log-rank *P*-value is shown. **G** Boxplots comparing the PCA_THCA_score distribution across the three OSPTCC subtypes. *P*-values from pairwise comparisons (e.g., Wilcoxon test) are indicated. **H** Principal component analysis (PCA) plot of TCGA-THCA samples based on the expression of the 34 MCD&PRGs. Points are colored by OSPTCC subtype assignment (Cluster 1 = Red, Cluster 2 = Green, Cluster 3 = Blue). **I** Sankey diagram illustrating the flow of patients between OSPTCC subtypes (geneCluster: Clusters 1–3), PCA score groups (group: low vs. high based on median), and survival status (censor: alive vs. dead). The width of the flows represents the number of patients

To bridge our single-cell discoveries with clinical applications in bulk tumor samples, we systematically analyzed the 1,404 malignant cell differentiation genes (MDGs). Through integration of temporal trajectory associations and clinical outcome data from the TCGA-THCA cohort (n = 510), we identified genes significantly correlated with PFS using Kaplan–Meier and univariate Cox regression analyses. Quantitative results revealed that 1,404 MDGs were initially derived from single-cell trajectory analysis, of which 487 genes showed significant prognostic associations in TCGA Kaplan–Meier analysis (*P* < 0.05). Further filtering via univariate Cox regression reduced this set to 112 genes, with 34 genes ultimately meeting all criteria for pseudotime correlation and clinical relevance. Through this multi-layered screening process, we identified and retained 34 genes concurrently characterized by malignant cell differentiation and significant prognostic associations. These genes were defined as malignant cell differentiation & prognosis-related genes (MCD & PRGs), upon which we subsequently built the OSPTCC subtyping and the LASSO-Cox prognostic model. The selection process, illustrated in Figure S3A through Venn diagrams, applied four stringent criteria to ultimately derive a refined malignant cell differentiation & prognosis-related gene set (MCD&PRGs) comprising 34 genes. The clinical relevance of these 34 genes was robustly validated in TCGA bulk data, with detailed Cox risk ratios and *P*-values presented in Figure S3B and corresponding Kaplan–Meier survival curves shown in Figure S3C. Co-expression network analysis of the MCD&PRGs (Fig. 4A) revealed distinct interaction patterns: Genes linked to the initial state (PTC1) exhibited strong positive correlations with PTC3 genes but negative correlations with PTC2 genes, reinforcing the concept of divergent differentiation pathways from PTC1 to either PTC2 or PTC3. Notably, the network topology further classified individual genes as either risk factors (red nodes, associated with poorer prognosis) or favorable factors (blue nodes, correlated with better survival), based on their Cox regression results.

Using the expression profiles of these 34 MCD&PRGs in the TCGA-THCA cohort, we performed concordance clustering analysis to identify robust molecular subtypes in bulk tumors (Fig. 4B, 4 C). The analysis demonstrated optimal stability at k = 3 clusters (Fig. 4C), defining our proposed oncogenic characteristic classification (OSPTCC) for papillary thyroid carcinoma as Cluster 1, Cluster 2, and Cluster 3. Heatmap analysis (Fig. 4D) revealed critical molecular associations: Cluster 1 exhibited relatively high expression of MCD&PRGs associated with both initial PTC1 and critical PTC3, including DEPTOR, ZFP36, FHL1, and SQSTM1, whereas Cluster 3 showed enrichment for genes linked to PTC1 and PTC2 such as APOE, APOC1, TSC22D1, and DUSP6. Cluster 2 displayed an intermediate expression pattern between these extremes. This stratification pattern establishes a direct molecular connection between Cluster 1 and the PTC3 transcriptional signature derived from the invasive malignant cell 1 subtype identified in single-cell analyses.

This molecular linkage demonstrated direct clinical relevance through survival outcome analyses. Kaplan–Meier analysis revealed that Cluster 1 (characterized by PTC3-associated features) exhibited significantly worse PFS (*P* = 4.468e−02, Fig. 4E), while Cluster 3 (PTC2-associated) showed the most favorable prognosis, with Cluster 2 displaying intermediate survival outcomes. Furthermore, PCA scores derived from MCD&PRG expression profiles were significantly reduced in Cluster 1 (*P* < 2.22e−16, Fig. 4G), corroborating the association between PTC3-driven molecular features, Cluster 1 classification, and adverse clinical outcomes, as supported by prognostic trend visualization in Fig. 4F. Principal component analysis (PCA) visualization demonstrated clear separation of the three clusters in reduced-dimensional space (Fig. 4H), where Cluster 1 (red) occupied a distinct region compared to Clusters 2 (green) and 3 (blue). The relationship between molecular subtypes (OSPTCC clusters), PCA-based stratification (low vs. high groups divided by median score), and clinical outcomes was further elucidated through Sankey diagram analysis (Fig. 4I). This visualization revealed that Cluster 1 was predominantly classified in the low PCA score group and showed a higher proportion of deceased patients relative to Clusters 2 and 3, reinforcing the association between Cluster 1 membership and poorer clinical outcomes.

This integrative approach, combining single-cell trajectory data, bulk-RNA-seq profiles, and clinical outcomes, therefore established three key differentiation states (PTC1-PTC3), a prognostic 34-gene signature (MCD&PRGs, Figure S3), and three molecularly distinct bulk tumor subtypes (Cluster 1–3). These subtypes demonstrated unique gene expression profiles corresponding to their differentiation states, with Cluster 1—characterized by molecular features of the aggressive malignant cell 1 subtype mapped to PTC3—exhibiting the poorest clinical prognosis, thereby creating a biologically informed framework for PTC risk stratification (Fig. 4) that directly links single-cell-derived differentiation trajectories to clinically actionable molecular subtypes.

### Construction and validation of a prognostic risk scoring model based on MCD&PRG features

**Fig. 5:**
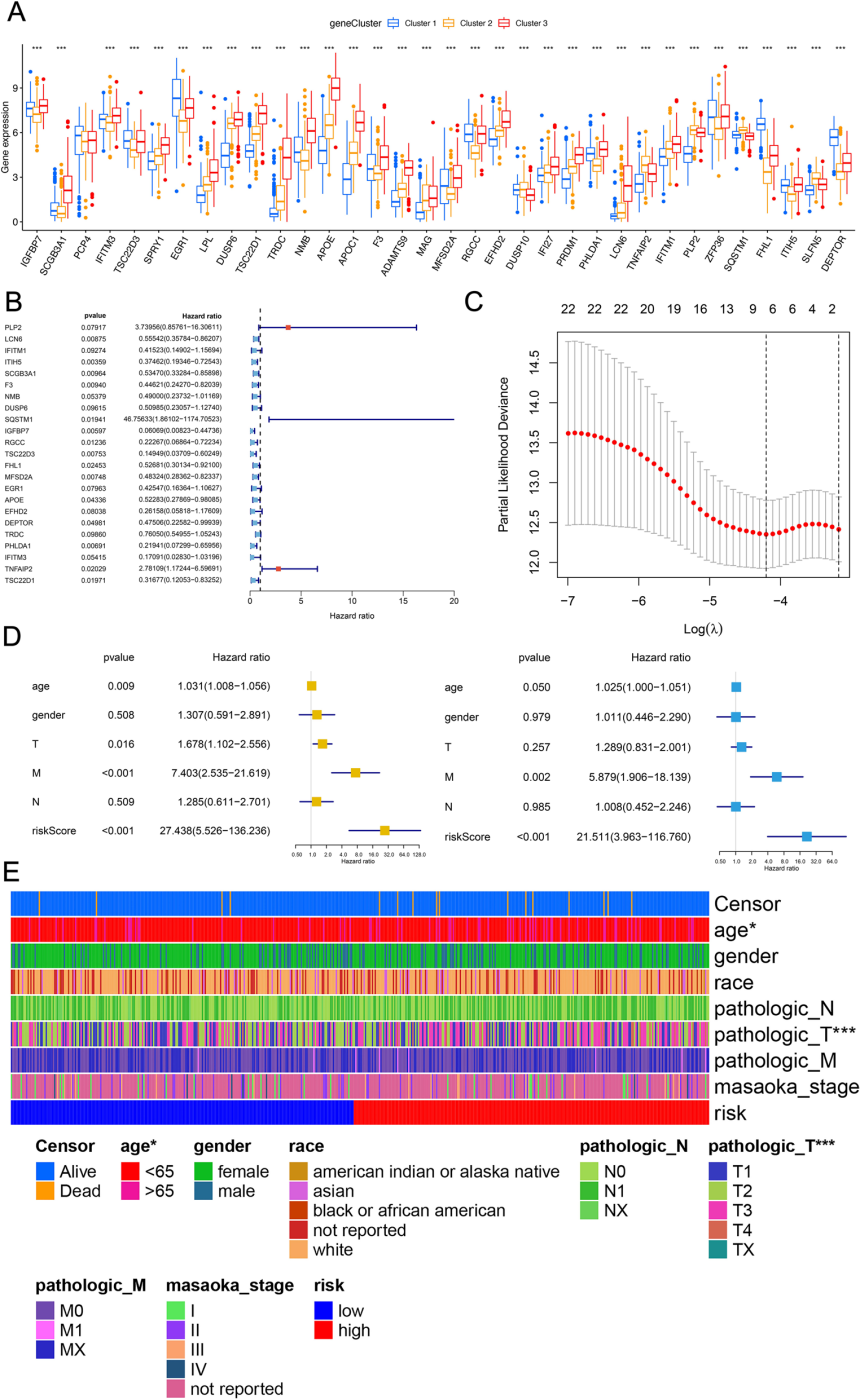
Development and validation of the MCD&PRG-based prognostic risk score. **A** Boxplots showing the expression levels of selected MCD&PRGs (likely those in the final risk model) across the three OSPTCC subtypes (Clusters 1, 2, 3) in the TCGA-THCA cohort. Asterisks denote statistical significance (*P < 0.05, **P < 0.01, ***P < 0.001). **B** Forest plot displaying the results of univariate Cox regression analysis for the 22 MCD&PRGs initially associated with PFS. Hazard ratios (HRs) and 95% confidence intervals (CIs) are shown for each gene. **C** LASSO regression ten-fold cross-validation plot. The Y-axis represents the partial likelihood deviance, and the X-axis represents the log(λ) penalty parameter. Vertical dashed lines indicate the lambda values corresponding to the minimum deviance (lambda.min) and the minimum deviance plus one standard error (lambda.1se). The numbers at the top indicate the number of nonzero coefficients (selected genes) at different lambda values. **D** Forest plots summarizing univariate (left) and multivariate (right) Cox regression analyses for PFS, including clinical variables (age, gender, pathological T, M, N stage) and the derived risk score. HRs and 95% CIs are shown. **E** Heatmap illustrating the association between the risk score groups (low vs. high risk) and various clinicopathological features in the TCGA-THCA cohort. Rows represent clinical features; columns represent individual patients ordered by risk score. Colors indicate different categories for each clinical variable as defined in the legend

Based on the 34 malignant cell differentiation and prognosis-related genes (MCD&PRGs) identified through integrated scRNA-seq and Bulk-RNA-seq analyses, we sought to develop a prognostic model for PTC. Our initial analysis confirmed that these MCD&PRGs showed differential expression patterns among the three OSPTCC subtypes (Cluster 1, 2, and 3) in the TCGA-THCA cohort, with many genes demonstrating statistically significant differences (marked by asterisks, Fig. 5A). These observations provide preliminary evidence supporting the potential utility of these genes in prognostic stratification.

To identify genes with the strongest independent prognostic value for PFS, we conducted univariate Cox proportional hazards regression analysis on all 34 MCD&PRGs using TCGA data. This initial screening identified 22 genes showing significant associations with PFS (*P* < 0.05), with their hazard ratios and confidence intervals visualized in the forest plot (Fig. 5B). To derive a more parsimonious and clinically practical prognostic signature while minimizing overfitting, we subsequently applied LASSO (Least Absolute Shrinkage and Selection Operator) Cox regression analysis to these 22 candidate genes. Through tenfold cross-validation, we determined the optimal penalty parameter (lambda), ultimately yielding a refined 7-gene prognostic signature (Fig. 5C). Subsequently, a risk score was calculated for each patient in the TCGA cohort based on the expression levels of these final characterized genes, weighted by their respective LASSO coefficients. Patients were then stratified into high- and low-risk groups using the median risk score as a threshold.

To evaluate the prognostic model's performance, we randomly divided the TCGA cohort into training (70%) and test (30%) sets. The seven signature genes showed significantly different expression levels between high- and low-risk groups in all datasets (whole cohort, training, and test sets; Figure S4A). Kaplan–Meier analysis revealed significantly worse PFS for high-risk patients across the entire cohort, training set, and test set (all *P* < 0.001, Figure S4B). Additional visualizations including gene expression heatmaps (Figure S4C), survival status scatter plots (Figure S4D), and risk score distributions (Figure S4E) consistently demonstrated the model's risk stratification capability, showing clear correlations between higher risk scores, elevated risk-gene expression, and increased progression events in all datasets. Time-dependent ROC analysis of the entire cohort yielded AUC values of 0.774 (all), 0.774 (training), and 0.763 (test), confirming the model's strong predictive accuracy for PFS events (Figure S4F).

To determine whether the risk score provided independent prognostic value beyond established clinical parameters, we conducted both univariate and multivariate Cox regression analyses incorporating the risk score along with clinical variables including age, sex, and pathological stage (T, N, M). Univariate analysis identified significant associations between PFS and several factors: the risk score, age, pathological T stage, and pathological M stage (Fig. 5D, left panel). Most notably, multivariate analysis demonstrated that the risk score maintained strong independent predictive value for PFS after adjustment for these clinical covariates (HR = 21.511, 95% CI = 3.963–116.760, *P* < 0.001; Fig. 5D, right panel), underscoring its potential clinical utility. These findings were further visualized by a comprehensive heatmap analysis that integrated risk group stratification (high/low), calculated risk scores, patient survival status (alive/deceased), and key clinical parameters within the TCGA cohort, revealing consistent correlations between elevated risk scores, unfavorable clinical characteristics, and poorer outcomes (Fig. 5E).

Thus, utilizing the differentiation-related prognostic gene signatures (MCD&PRGs), a risk scoring model containing seven genes was successfully constructed and validated. This model proved effective in categorizing PTC patients into high-risk and low-risk groups with significantly different PFS outcomes, demonstrated good predictive accuracy, and served as an independent prognostic factor beyond traditional clinical parameters.

### Multi-omics characterization links OSPTCC classification to genomic alterations, functional pathways, and immune microenvironment

To further investigate the molecular underpinnings of the OSPTCC classification, we performed comprehensive multi-omics integration using TCGA-THCA data. Somatic mutation analysis confirmed the anticipated high prevalence of BRAF mutations, with co-occurring RAS mutations (Figure S5A-B oncoplots). Prognostic stratification by PCA score revealed distinct mutational patterns, with Cluster 1 (poor prognosis/low PCA score) showing reduced BRAF mutation frequency and a potential increase in RAS mutations compared to other clusters.

We further examined the interplay between Tumor Mutational Burden (TMB) and our risk score model. Kaplan–Meier analysis revealed significant survival differences (*P* < 0.001) when patients were stratified by both parameters, with the high TMB/high-risk subgroup demonstrating markedly worse outcomes (Figure S5C).

Copy number variation (CNV) analysis of the 34 MCD&PRGs identified frequent genomic alterations, including recurrent amplifications (> 20% frequency) of SCGB3A1, EGR1, DUSP10, and SQSTM1, and deletions of PRDM1, ZFP36, FHL1, DEPTOR, and SPRY1 (Figure S5D). These dosage-altering events likely contribute to the expression patterns characterizing each subtype. Genome-wide visualization via Circos plot confirmed the chromosomal distribution of MCD&PRGs and highlighted regions of genomic instability (Figure S5E).

To elucidate the functional pathways driving the prognostic differences associated with our classification (represented by the risk score derived from MCD&PRGs), we performed enrichment analyses comparing high-risk versus low-risk groups. Overall Representation Analysis suggested broad functional differences across MSigDB categories between the groups (Figure S6A). Gene Set Enrichment Analysis (GSEA) provided more specific insights. Consistent with the designation for the high-risk Cluster 1, GSEA revealed significant enrichment of inflammatory and immune response pathways based on Gene Ontology Biological Process terms (GOBP, e.g., Activation of Immune Response, Adaptive Immune Response), KEGG pathways (e.g., Cell Adhesion Molecules, Cytokine–Cytokine Receptor Interaction), and Hallmark pathways (e.g., Inflammatory Response, Interferon Alpha Response, Allograft Rejection) in the high-risk group (Figure S6B-D, left panels). Conversely, the low-risk group (corresponding to Cluster 3) showed significant enrichment primarily in metabolic processes, particularly those related to lipid metabolism (e.g., Hallmark Fatty Acid Metabolism, Bile Acid Metabolism, Adipogenesis) and other diverse functions like axoneme assembly or calcium signaling (Figure S6B-D, right panels). These GSEA results strongly support our core hypothesis, linking the poor prognosis of the high-risk group (Cluster 1) specifically to heightened inflammatory pathway activity, while associating the better prognosis of the low-risk group (Cluster 3) with distinct metabolic features.

Assessment of the TME revealed significant differences associated with the subtypes. Quantification of immune cell infiltration showed distinct profiles across the OSPTCC clusters (Figure S7A). Cluster 1 (poor prognosis) displayed significantly higher infiltration of pro-inflammatory/effector cells like activated CD4 +/CD8 + T cells and activated dendritic cells (*P* < 0.001), aligning with its inflammatory functional enrichment seen in GSEA. In contrast, Cluster 3 (better prognosis) was characterized by higher levels of Mast cells and relatively lower activated cytotoxic lymphocytes compared to Cluster 1. Cluster 2 generally showed intermediate levels. While correlation analysis indicated that a higher PCA score (characteristic of Cluster 3) was associated with increased infiltration levels of many immune cell types (Figure S7C), suggesting significant overall immune cell recruitment, the functional GSEA results (Figure S6) imply this infiltrate differs in its activation state or functional polarization compared to the strongly pro-inflammatory signature of Cluster 1. Analysis of PD-L1 (CD274) expression showed significantly higher levels in the high PCA score group (Cluster 3) (*P* = 0.018) (Figure S7B), which might reflect complex regulation or an exhausted phenotype in this metabolically distinct, yet immune cell-rich, subtype.

Finally, comparing PTC tumors to adjacent normal tissues provided essential context for the overall molecular changes. Differential expression analysis identified thousands of DEGs clearly separating the tissue types (Figure S8A Heatmap, S8B Volcano plot). Crucially, the 34 MCD&PRGs themselves were significantly differentially expressed between tumor and normal tissues, with genes like APOE, APOC1, and SQSTM1 being upregulated and DEPTOR, FHL1, PCP4, and ZFP36 downregulated in tumors, validating their strong association with the cancerous state (Figure S8E Heatmap, S8F Volcano plot). Similarly, numerous transcription factors (DETFs) showed differential expression, including upregulation of ATF3 and EGR1 and downregulation of FOXA2 and SREBF1 in tumors (Figure S8C Heatmap, S8D Volcano plot), pinpointing potential regulatory drivers. GSVA confirmed extensive pathway deregulation in tumors compared to normal tissue, with significant upregulation of pathways related to cell cycle, oncogenic signaling (MYC, KRAS, PI3K/AKT/mTOR), metabolism (Glycolysis), inflammation, and EMT (Figure S8G Heatmap, S8H Bar plot). Together, these analyses confirm the extensive molecular reprogramming in PTC, validate the cancer relevance of the MCD&PRGs, and identify key genomic, transcriptomic, pathway, and immunological features associated with the OSPTCC subtypes, setting the stage for constructing regulatory networks.

### Construction of regulatory network for each subtype of OSPTCC

**Fig. 6:**
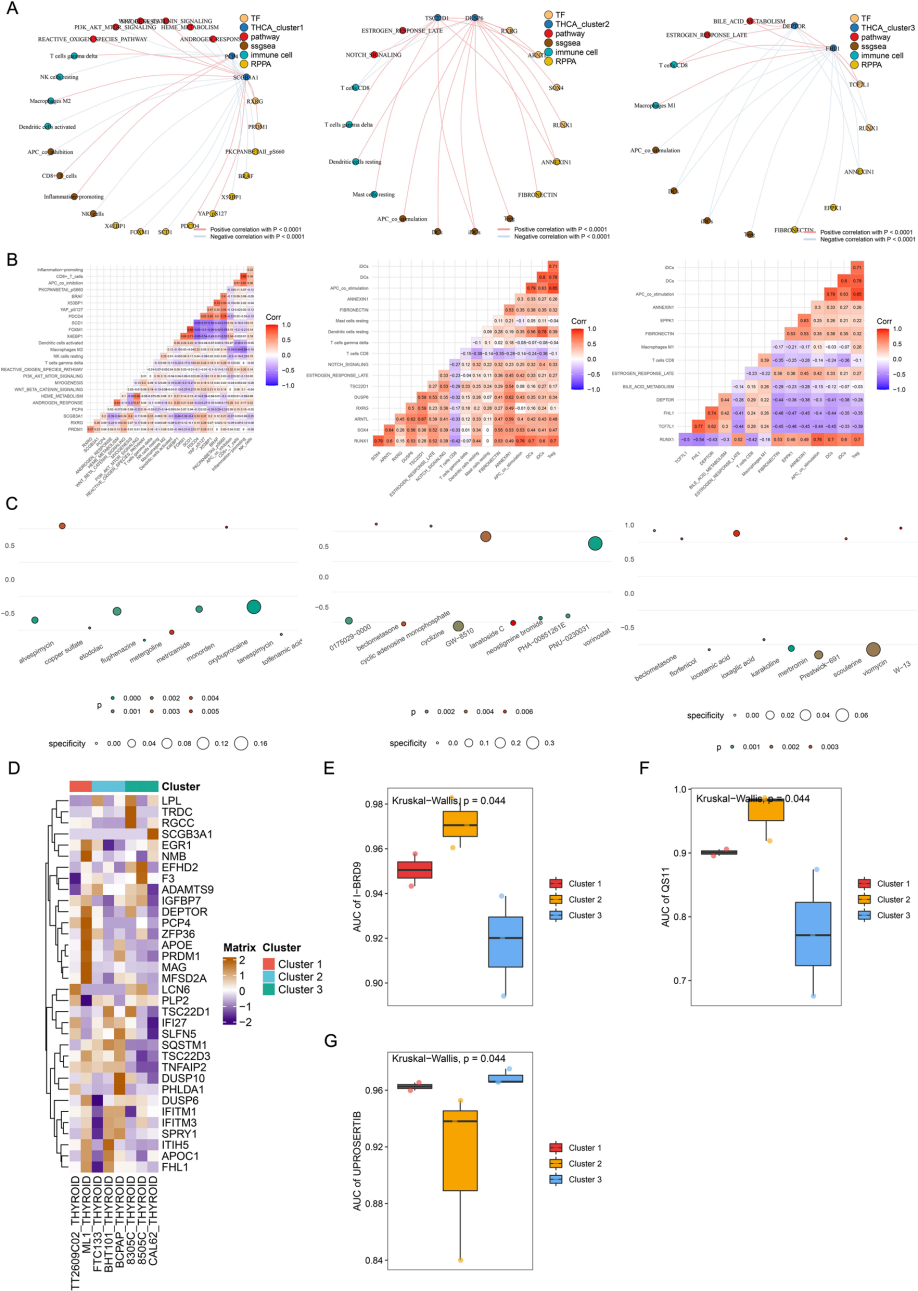
Subtype-specific regulatory networks and therapeutic predictions. **A** Network diagrams illustrating potential regulatory interactions within each OSPTCC subtype (Cluster 1/IPTCC, Cluster 2/BAPTCC, Cluster 3/LPTCC). Nodes represent transcription factors (TF), core MCD&PRGs (THCA_cluster1/2/3), signaling pathways (pathway), immune cell infiltration scores (ssgsea), immune cell types (immune cell), and RPPA protein levels (RPPA). Edges indicate significant correlations (Pearson/Spearman |R|> 0.3, *P* < 0.001), with red lines for positive and blue lines for negative correlations. **B** Corresponding heatmaps showing the correlation coefficients between selected components within each subtype-specific network depicted in **A**. Red indicates positive correlation; blue indicates negative correlation. **C** Bubble plots summarizing Connectivity Map (CMap) predictions for small molecule inhibitors targeting each OSPTCC subtype signature. Bubble size represents specificity score; color intensity represents statistical significance (*P*-value). Top candidate drugs are labeled. **D** Heatmap displaying the expression levels (scaled Z-score) of the 34 MCD&PRGs across a panel of thyroid cancer cell lines. Rows represent genes; columns represent cell lines. (E–G) Boxplots showing the predicted drug sensitivity (area under the curve, AUC; lower AUC indicates higher sensitivity) based on GDSC data for specific inhibitors across the three OSPTCC clusters. **E** Sensitivity to I-BRD9. **F** Sensitivity to QS11. **G** Sensitivity to UPROSERTIB. Kruskal–Wallis test *P*-values are indicated

To further investigate the potential key regulatory mechanisms of each OSPTCC subtype, we applied co-expression analysis based on the quantitative results of uniformly standardized DETFs, MCD&PRGs, immune cells/immune functions, and signaling pathways of each OSPTCC subtype and constructed three MCD&PRGs as regulatory cores with correlation coefficients *R* > 0.300 and *P* < 0.001 as the minimum screening criteria, respectively. A complex regulatory network containing upstream TFs, downstream signaling pathways, and potential regulation of immune cells/function was constructed (Fig. 6A). Based on the gene expression pattern, inhibitor analysis, and regulatory network integration analysis, we named the Cluster1–3 as: Inflammation-associated Papillary Thyroid Carcinoma Classification (IPTCC), BRAF mutation and Autophagy-related Papillary Thyroid Carcinoma Classification (BAPTCC), and Lipid metabolism-related Papillary Thyroid Carcinoma Classification (LPTCC). We also used co-expression heat maps to show the specific correlation coefficients for the correlation analysis in each regulatory network (Fig. 6B).

Prediction of small molecule active drugs targeted by each subtype of OSPTCC. To further integrate our proposed clinical staging with clinical treatment decisions, we first identified small molecule drugs targeting MCD&PRGs and TFs in the regulatory network of each OSPTCC subtype by using the Cmap algorithm and screened the 10 drugs with the smallest *P*-values for visualization using dotted heat maps as target drugs for each OSPTCC subtype (Fig. 6C). Then with the help of GDSC database, drug sensitivity analysis of cell fate-related genes was performed and obtained specific inhibitors I-BRD9 and QS11 in BAPTCC and UPROSERTIB in LPTCC (Fig. 6D-G). By integrating the results of Cmap and GDSC database, AKT pathway inhibitor LY209001 [36], WNT pathway sensitizer QS11 [37], and AKT pathway inhibitor UPROSERTIB [38] were found to be specific for IPTCC, BAPTCC, and LPTCC, respectively.

### PCR validation

To experimentally validate whether the gene expression characteristics underpinning the OSPTCC subtypes translate to prognostic differences, we analyzed an independent cohort. From an initial set of 76 patients diagnosed with PTC, we utilized qRT-PCR to assess the expression levels of four characteristic genes associated with OSPTCC subtypes (APOE, APOC1, DEPTOR, and SQSTM1) in patient tumor samples.

Patients were stratified into high-risk and low-risk groups based on a stringent definition using the median expression values of DEPTOR, APOE, and APOC1 across the 76 samples. The high-risk group (n = 25) was defined by DEPTOR expression > median AND (APOE expression < median OR APOC1 expression < median). Conversely, the low-risk group (n = 23) was defined by DEPTOR expression < median AND (APOE expression > median OR APOC1 expression > median). Patients not conforming to these specific profiles (n = 28) were excluded from this comparative analysis, resulting in 48 patients for the final validation set.

**Fig. 7:**
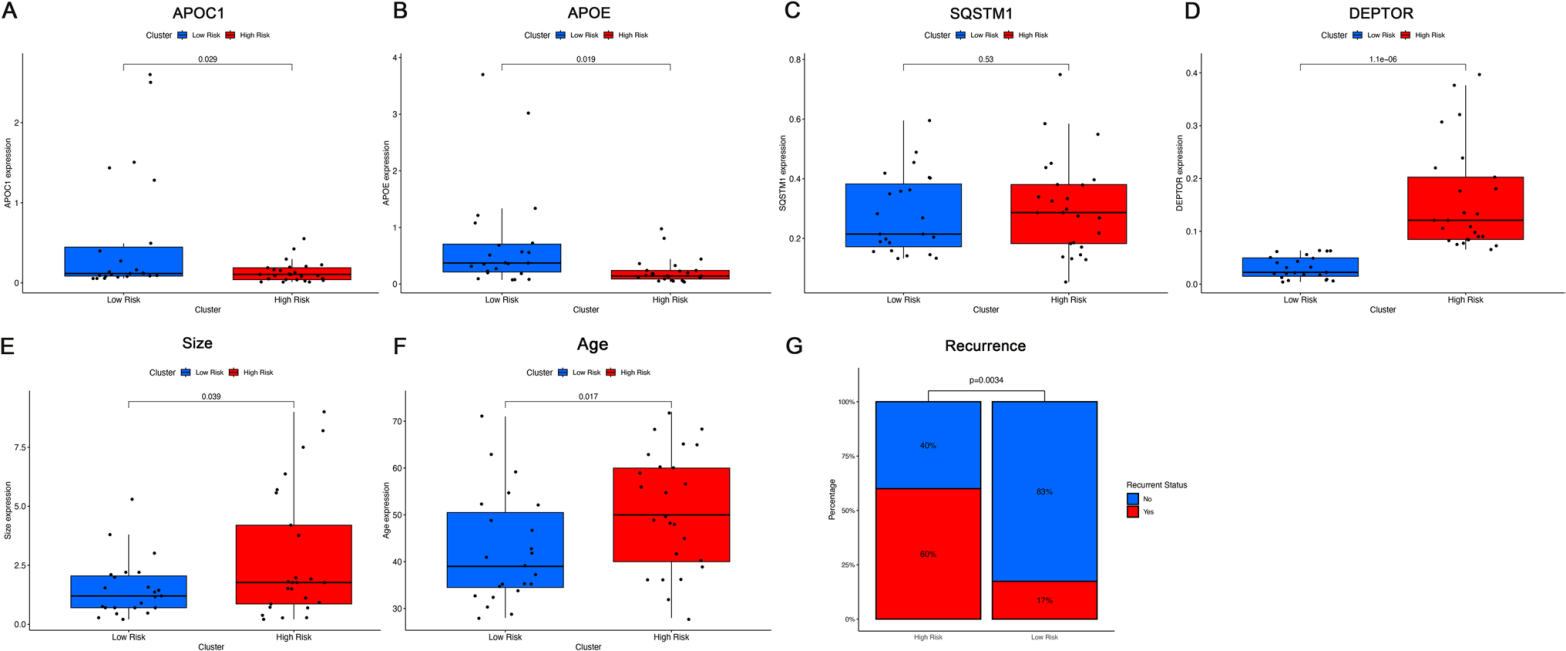
qRT-PCR and clinical feature validation of OSPTCC typing. **A** Differential expression of the APOC1 gene between high- and low-risk groups. **B** Differential expression of the APOE gene between high- and low-risk groups. **C** Differential expression of the SQSTM1 gene between high- and low-risk groups. **D** Differential expression of the DEPTOR gene between high- and low-risk groups. **E** Difference in tumor size between high-risk and low-risk groups. **F** Difference in age between high-risk and low-risk groups. **G** Difference in the incidence of recurrence events between high-risk and low-risk groups

Analysis of prognostic-related clinical features for these 48 stratified patients was conducted (Table 1). Figure 7A-D demonstrates the expression levels of the characteristic genes in the high-risk and low-risk patient groups, confirming specific expression patterns (e.g., higher DEPTOR in the high-risk group, higher APOE and/or APOC1 in the low-risk group, and vice-versa for the opposing risk group).

When comparing clinical indicators related to PTC prognosis, the high-risk group showed significantly older age at diagnosis (Mean ± SD: 51.08 ± 12.63 yrs vs. 42.43 ± 11.67 yrs, *P* = 0.017) and significantly larger tumor size (Mean ± SD: 2.79 ± 2.70 cm vs. 1.51 ± 1.22 cm, *P* = 0.039) compared to the low-risk group. Most notably, the recurrence rate was significantly higher in the high-risk group (60.00% vs. 17.39%, *P* = 0.007). However, the lymph node metastasis rate did not show a statistically significant difference between the two groups in this re-stratified cohort (Figs. 7E-G, Table 1). Other features such as gender, multifocality, thyroiditis, presence of thyroid nodules, and mutation status of KRAS, BRAF, and TERT did not significantly differ between the high- and low-risk groups in this validation set.

Overall, the significant differences in tumor size and particularly in recurrence rate suggest that this 3-gene expression signature (DEPTOR, APOE, APOC1, with the specified OR logic for APOE/APOC1) holds potential for distinguishing PTC patients with differing prognostic outcomes, broadly consistent with the prognostic implications derived from our primary bioinformatics analysis of OSPTCC subtypes.

## Discussion

By integrating scRNA-seq and Bulk-RNA-seq data, this study introduces the OSPTCC classification, a novel molecular subtyping system for papillary thyroid cancer (PTC) derived from single-cell differentiation trajectories. Grounded in distinct cellular states (PTC1-3) reflecting inferred differentiation pathways, OSPTCC offers a unique biological perspective compared to traditional risk stratification systems. Our institutional cohort validation, assessing key characteristic genes (DEPTOR, APOE, APOC1, SQSTM1) via qRT-PCR in 76 PTC patients (48 stratified into high/low-risk groups), demonstrated that this OSPTCC-based stratification correlated with older age, larger tumors, and significantly higher recurrence rates in the high-risk group, suggesting its potential to distinguish prognostic outcomes.

We propose OSPTCC as a complementary molecular layer to existing frameworks, potentially refining risk assessment. A key strength, supported by validation, is its linkage to distinct biological mechanisms: The IPTCC subtype, characterized by high DEPTOR expression and associated with a poorer prognosis in our validation cohort, points toward a significant inflammatory component. This connection to inflammation is particularly intriguing given the increasing emphasis on the tumor microenvironment and its interplay with conditions like autoimmune thyroid diseases, a theme we will explore in detail. Similarly, the BAPTCC subtype is associated with autophagy/BRAF signaling (linked to SQSTM1), and the LPTCC subtype with lipid metabolism (characterized by APOE/APOC1), each with their distinct regulatory networks. These mechanistic insights not only enhance our understanding of PTC pathobiology but also pave the way for investigating subtype-specific vulnerabilities and therapeutic strategies, as suggested by our preliminary drug sensitivity predictions. However, we emphasize that OSPTCC is currently a research-based classification derived from retrospective data analysis, and its translation into a clinically applicable tool necessitates rigorous prospective validation and the development of a standardized diagnostic assay.

Given the increasing emphasis on the importance of the tumor microenvironment in cancer research, the impact of autoimmune diseases on tumors is attracting attention, but the underlying mechanisms remain unclear [39]. Studies suggest that long-term, persistent immune stimulation caused by autoimmune diseases may promote tumor progression [40]. The relationship between autoimmune thyroid diseases (AITDs) and PTC has been widely debated in recent decades. AITDs, a group of common diseases characterized by immune system dysregulation leading to T cell mediated thyroid damage, present with diverse clinical manifestations [41]. Research data indicate that chronic thyroiditis accompanies 20–50% of PTC samples [42]. Similarly, lymphocytic infiltration (LI) around the lesion in PTC or lymphocytic infiltrative thyroiditis lesions are significantly more common than in benign thyroid lesions [43, 44]. Although the exact mechanism of interaction between thyroiditis and thyroid malignancy is unclear, there is no doubt that autoimmune thyroiditis and its corresponding mechanism of inflammatory action is an inescapable link in thyroid tumor research.

Interestingly, key genes differentially highly expressed in IPTCC (TSC22D3, ZFP36, DEPTOR) also exhibited features associated with inflammatory mechanisms, suggesting a potential mechanism of action for IPTCC.

TSC22D3, also known as glucocorticoid-induced leucine zipper (GILZ), is thought to be an important transcription factor regulating apoptosis [45]. In a series of studies, TSC22D3 was shown to be an important mediator of the anti-inflammatory effects of glucocorticoids and to inhibit pro-inflammatory gene expression [46–48]. TSC22D3 is also thought to be important in autoimmune inflammation-related regulation [49]. In addition, TSC22D3 is thought to be involved in monitoring behaviors that disrupt anti-tumor immunity, and this behavior may be associated with altered psychological states of the body, such as stress [50]. Several previous investigations [51–53] have suggested that altered mental status may be a potential risk factor for thyroid cancer. This suggests that the role of TSC22D3 in IPTCC may not be limited to inflammation promotion, and its role in tumor immunity deserves further attention.

ZFP36 is an important member of the ZFP36 family, characterized by one or more CCCH-type zinc finger structural domains containing three cysteinese (C) and one histidine (H) residues. ZFP36 family proteins can bind to specific sites of target mRNAs, leading to mRNA decay [54]. ZFP36 interferes with TNF-α [55], IL-6 [56], and IL-10 [57] levels in vivo through post-transcriptional regulatory mechanisms in multiple pathways to control the initiation and regression of inflammatory responses. Although the exact mechanism is unclear, ZFP36 is closely associated with a variety of inflammatory diseases[58–61]. Due to its ability to regulate mRNA stability and its loss of expression in various human malignancies, ZFP36 is considered a potential tumor suppressor [62]. This view has been confirmed in several tumor studies covering malignant glioma [63], breast cancer [64], and colon cancer [65]. However, a recent study [66] on the mechanism of action of ZFP36 in hepatocellular carcinoma seems to suggest the complexity of ZFP36 in the mechanism of tumor action. This study observed that inhibition of ZFP36 expression exerted anti-tumor effects while suppressing hepatic inflammatory and lipid synthesis responses. The results suggest that since hepatocarcinogenesis is associated with inflammatory and lipid metabolic mechanisms, high ZFP36 expression may act as a promoter of tumor development in the early stages of tumorigenesis rather than a purely anti-tumor effect, which is consistent with the high expression of ZFP36 that we found in BATCC. Despite the differences in the mechanisms of hepatocellular carcinoma and thyroid tumorigenesis, inflammation, and lipid metabolism are equally important promoting mechanisms in thyroid cancer [67, 68]. All of the above information suggests that ZFP36 may be involved in thyroid cancer progression by affecting inflammation and lipid metabolism, corroborating with the results found about TSC22D3.

DEPTOR is a 409-amino acid/48-kDa protein only found in vertebrates. It contains two tandem NH2-terminal DEP domains and a COOH-terminal PDZ domain [69, 70]. As an important regulator of mTOR signaling, DEPTOR exerts complex regulatory effects on multiple aspects of the mTOR signaling pathway, mainly by interacting with mTORC1 and mTORC2. DEPTOR was found to play opposite regulatory roles in different tumors through two different regulatory mechanisms. DEPTOR was shown to be an oncogene in hepatocellular carcinoma [71], ERα-negative breast cancer [72] and esophageal squamous cell carcinoma [73], multiple myeloma [70] and differentiated thyroid cancer [74], possibly through a"feedback model"mechanism. In this mechanism, DEPTOR overexpression leads to inhibition of MTOC1 and activation of AKT pathway expression [71, 72, 75–77], while DEPTOR was found to be a tumor suppressor in pancreatic cancer [78], ESCC [79], lung cancer [80], triple-negative breast cancer [72], and B-ALL [76]. The role of DEPTOR found in previous studies is consistent with the results of our analysis. At the time of the drug analysis, LY209001, an AKT pathway inhibitor [36], specifically inhibited IPTCC, suggesting that the AKT pathway may be activated in this phenotype, as corroborated by the results of the regulatory network. At this point, we conclude that IPTCC typing may initiate inflammatory mechanisms through various inflammatory molecules such as TSC22D3 and ZFP36, and exert oncogenic effects through activation of AKT pathway by DEPTOR, which may also involve more complex inflammatory cancer transformation and disruption of anti-tumor immunity. This was confirmed in our subsequent PCR experiment validation, where patients in the high-risk group with high DEPTOR expression exhibited poorer prognostic outcomes (Figs. 7E-H). And the specific mechanism of typing still needs further molecular experimental validation to confirm.

BAPTCC regulatory network and differential expression of key genes suggest that TSC22D1 and DUSP6 are involved in building the BAPTCC regulatory network, while SQSTM1 is specifically highly expressed in BAPTCC. These three genes in this typology constitute an interesting regulatory relationship, corroborated by the drug sensitivity results of previous studies and the present study mentioned below. The BRAF^V600E^ gene mutation is the most common in PTC [81, 82]. Nevertheless, targeted therapy for BRAF-mutant PTC is still in its infancy. The study demonstrates that DUSP6 and TSC22D1 are significantly upregulated in BRAF^V600E^ mutant thyroid cancer and play an important role in the escape mechanism of tumor cells from OIS [83, 84]. Oncogene-induced senescence (OIS) is a growth arrest triggered by the forced expression of cancer-promoting genes and acts as a barrier to malignant transformation in vivo [85, 86]. OIS is an important protective factor against carcinogenesis in various tumors, including BRAF^V600E^ mutant PTC [87]. The active expression of DUSP6 and TSC22D1 in BAPTCC suggests that BRAF^V600E^ mutation may be one of the important mechanisms of BAPTCC.

At the same time, the phenomenon of SQSTM1-specific high expression in this phenotype suggests a more complex pathogenic mechanism. The relationship between SQSTM1 and autophagy has been reported in several studies [88, 89]. SQSTM1 participates in the autophagic process as an important molecule that constitutes the autophagosome and plays an important role in the regulation of autophagy [90]. This suggests that autophagy may be involved in the pathogenic mechanism of BAPTCC. And this conjecture is supported by additional evidence in the study of the relationship between BRAF genes and autophagy, where autophagy is considered a suppressor of BRAF-driven malignancies [91]. In particular, combined inhibition of oncogenic signaling and autophagy is an effective strategy for treating cancers driven by oncogenic KRAS or BRAF [92–95]. Notably, the WNT pathway was shown to form a regulatory chain with SQSTM1 in autophagy regulation, and WNT signaling led to autophagy inhibition by inhibiting the SQSTM1 promoter [96]. In summary, high expression of DUSP6 and TSC22D3 suggests that BATCC is driven by BRAF mutations, and high expression of SQSTM1 suggests that the WNT pathway and autophagy mechanisms may be involved in BAPTCC pathogenesis, which is corroborated by drug-sensitive phenotype analysis: The WNT sensitizer QS11[37] is specific for BAPTCC. From this, we infer that the pathogenic mechanism of BAPTCC is associated with BRAF-mutant PTC and autophagy involving DUSP6 and TSC22D3 and may act through the WNT pathway.

Fatty acid metabolism is an important metabolic process that affects cancer progression. Studies have demonstrated that fatty acid metabolism alters tumor progression, affecting energy storage, regulating cell proliferation, and stimulating the extracellular environment [97–99]. The effect of fatty acid metabolism on thyroid function [100] and the prognosis of differentiated thyroid cancer [101] have also been demonstrated. And among fatty acid metabolism, lipid metabolic reprogramming has become a significant direction in studying lipid metabolism. Lipid metabolic reprogramming is one of the types of metabolic reprogramming that is considered one of the characteristics of tumors. Abnormally active fatty acid (FA) synthesis and/or β-oxidation is major features of FA metabolic reprogramming [102, 103]. And inhibition of key enzymes of FA synthesis and β-oxidation (e.g., acetyl coenzyme A carboxylase, acetyl coenzyme A synthase, ATP citrate lyase, and carnitine palmitoyltransferase 1 C) inhibits tumor cell growth and delays disease progression [104–108]. Thus FA metabolic reprogramming is thought to be involved in tumor progression. At the same time, several studies have demonstrated changes in FA levels in PTC specimens [109–112], suggesting that FA metabolism may be involved in the development of PTC. And this metabolic process is reflected in changes in LPL levels, which were significantly upregulated in PTC specimens in lipidomic analysis. Elevated LPL expression was also observed in the GEO dataset (GSE104006) in thyroid cancer specimens [113]. The consistency in the levels of LPL, FA changes in PTC, and the known relationship between LPL and FA metabolism suggest that LPL may play a crucial role in thyroid progression by promoting FA metabolism. Also, lipid metabolism studies have identified upregulation of LPL in PTC and a direct correlation between LPL expression and tumor size and lymph node metastasis [101]. It can be concluded that LPL somewhat suggests the adverse progression of PTC.

Interestingly, in our analysis results, APOE and APOC1 molecules were equally highly expressed in LPTCC. APOE and APOC1 belong to the same APOE/APOC1/APOC2 gene cluster, while APOC1 is a potent inhibitor of LPL-mediated TG lipolysis [114]. This seems to explain the better prognosis of LPTCC than IPTCC and BAPTCC in the results of this study, and we speculate that APOE and APOC1 synergistically antagonize the oncogenic effects of LPL, forming a complex regulatory relationship and relatively better clinical prognostic outcome.

In the results of the inhibitor analysis, the broad-spectrum inhibitor of the AKT pathway, UPROSERTIB [38], showed specificity for LPTCC, and consistent with this result, activation of the AKT pathway was shown to increase lipid metabolism levels, suggesting that the AKT pathway may be involved in the pathogenic process of LPTCC by affecting lipid metabolism.

Although LPTCC has a distinctive lipid metabolism profile, inflammation-related genes expressed in ITCC (TSC22D3, ZFP36) and BRAF mutation-related genes expressed in BAPTCC (TSC22D1, DUSP6) are all expressed in subtype 3. Still, this feature is not reflected in the regulatory network. We speculate that these two mechanisms may not play a major role in the pathogenesis of LPTCC, and further experimental verification is needed regarding the specific mechanism of the complex pathogenic relationship of LPTCC.

We acknowledge several limitations inherent in this study. Our findings are primarily derived from publicly available datasets (GEO GSE184362, TCGA-THCA), which may carry intrinsic selection biases. The TCGA-THCA bulk cohort we used for molecular subtyping is overwhelmingly composed of PTC, yet it does contain a very small fraction of non-PTC thyroid malignancies (< 2%). We elected to retain these samples because (i) their numerical weight is negligible, (ii) our subtype-defining gene set was distilled from single-cell PTC data and therefore dominates the clustering signal. Nonetheless, this choice introduces a modest source of histological heterogeneity that could, in theory, dilute subtype-specific effects. Future prospective studies restricted to pathologist-re-adjudicated PTC cases will be necessary to confirm that the OSPTCC framework and the associated risk model are fully generalizable within strictly defined PTC populations. The foundational single-cell RNA sequencing analysis, while revealing key insights, was based on data from 11 patients (GSE184362), raising considerations about the generalizability of the inferred differentiation trajectories and initial subtypes that warrant confirmation in larger, more diverse single-cell cohorts. Furthermore, our independent experimental validation, although providing valuable proof-of-concept by correlating key marker expression with adverse clinical features in 48 patients, was limited in sample size and scope, focusing on four representative genes rather than the full 34-gene MCD&PRG signature or the derived risk score. Consequently, while supportive of the underlying biology, this does not constitute a definitive validation of the specific multi-gene prognostic model itself. The retrospective nature of the study necessitates large-scale prospective investigations to firmly establish the predictive power and clinical utility of the OSPTCC subtypes and the associated risk score. Additionally, while our integrated analyses suggest plausible regulatory mechanisms for each subtype, these remain largely bioinformatic inferences requiring direct experimental validation through functional studies (in vitro and in vivo) to confirm causality. Translating the RNA-based signature into a robust and clinically practical assay presents a further challenge. Finally, while our model captures significant heterogeneity linked to differentiation state, it may not encompass the full spectrum of PTC's molecular complexity, including the influence of rare histological variants or detailed interactions with specific driver mutations beyond the trends observed. These limitations underscore that while our study provides a novel framework and significant insights, further research, including functional experiments and prospective clinical trials, is crucial to fully validate these findings and explore their translational potential, representing key directions for our future work.

## Conclusion

In this study, we successfully developed and validated a novel molecular classification system for papillary thyroid cancer, termed OSPTCC, by integrating single-cell RNA sequencing insights with bulk multi-omics data. Our findings demonstrate that PTC can be stratified into three distinct subtypes—inflammation-associated (IPTCC), BRAF/autophagy-related (BAPTCC), and lipid metabolism-related (LPTCC)—each characterized by unique biological mechanisms and significantly different clinical prognoses. The OSPTCC framework, along with a derived 7-gene prognostic risk score, offers a more nuanced understanding of PTC heterogeneity beyond traditional clinicopathological features, and our experimental validation provided initial support for its clinical relevance, particularly in identifying patients at higher risk of recurrence. Collectively, this research provides a new lens through which to view PTC pathobiology, highlighting the importance of tumor cell differentiation states. The OSPTCC classification holds the potential to refine patient risk stratification, identify novel therapeutic targets specific to each subtype, and ultimately contribute to the development of more precise and personalized treatment strategies for PTC patients, warranting further large-scale prospective studies to fully translate these findings into routine clinical practice.

## Supplementary Information

Below is the link to the electronic supplementary material.

Supplementary file1 (ZIP 131 KB)

Supplementary file2 (ZIP 46990 KB)

Supplementary file3 (ZIP 77 KB)

## Data Availability

The scRNA-seq data of thyroid tumor cells were obtained from the GSE184362 dataset, which includes the transcriptome of 158,577 cells from 11 patients. Genomic profiles, RNA sequencing profiles, reverse-phase protein array (RPPA) profiles and transposase sequencing profiles (ATAC-seq), demographic characteristics, and clinical information profiles such as overall survival were obtained from The Cancer Genome Atlas (TCGA) database (http:/Acga-data.nci.nihgov/tega), including 510 THCA tumor tissues and 58 adjacent non-tumor tissues. Moreover, all raw data were downloaded from public databases, and no additional ethical proof was required. The specific data in each figure are compiled in Excel files. All the codes generated during the data analysis process in this study are stored in code file. Any additional information required to reanalyze the data reported in this paper is available from the correspondence author upon request.
