# Supplementary material for: Single-cell RNA sequencing and multi-omics analysis of prognosis-related staging in papillary thyroid cancer
Source: Cancer Immunol Immunother. 2025 Jul 12;74(8):267. doi: 10.1007/s00262-025-04101-4 (PMC12255609; doi:10.1007/s00262-025-04101-4)
Supplement: Supplementary file 1 — Supplementary file1 (ZIP 131 KB) [file 262_2025_4101_MOESM1_ESM.zip › New folder1/code_SC.docx]

cd /home/lq/scRNAseq/GSE184362_PTC

R

library(limma)

library(Seurat)

library(dplyr)

library(magrittr)

library(SingleR)

library(reticulate)

library(cowplot)

library(stringr)

library(RColorBrewer)

library(ggplotify)

library(cowplot)

library(ggplot2)

library(patchwork)

library(ggalluvial)

library(tidyverse)

library(patchwork)

library(reshape2)

library(scales)

library(ggpubr)

library(pheatmap)

setwd("/home/lq/scRNAseq/GSE184362_PTC")

#pbmc = readRDS("pbmc.rds")

#read.data

#read.data

#################################

sm1 <- Read10X(data.dir = "/home/lq/scRNAseq/GSE184362_PTC/GSM5585102")

sm1 <- CreateSeuratObject(counts = sm1, project = "thyroid tumor 1", cells = 3, min.features = 200)

P1 <- ScaleData(object =sm1)

sm2 <- Read10X(data.dir = "/home/lq/scRNAseq/GSE184362_PTC/GSM5585103")

sm2 <- CreateSeuratObject(counts = sm2, project = "thyroid paratumor 1", min.cells = 3, min.features = 200)

P2 <- ScaleData(object =sm2)

sm3 <- Read10X(data.dir = "/home/lq/scRNAseq/GSE184362_PTC/GSM5585104")

sm3 <- CreateSeuratObject(counts = sm3, project = "thyroid tumor 2", min.cells = 3, min.features = 200)

P3 <- ScaleData(object =sm3)

sm4 <-Read10X(data.dir = "/home/lq/scRNAseq/GSE184362_PTC/GSM5585105")

sm4 <- CreateSeuratObject(counts = sm4, project = "thyroid paratumor 2", min.cells = 3, min.features = 200)

P4 <- ScaleData(object =sm4)

sm5 <- Read10X(data.dir = "/home/lq/scRNAseq/GSE184362_PTC/GSM5585106")

sm5 <- CreateSeuratObject(counts = sm5, project = "lymph node metastase 1", min.cells = 3, min.features = 200)

P5 <- ScaleData(object =sm5)

sm6 <- Read10X(data.dir = "/home/lq/scRNAseq/GSE184362_PTC/GSM5585107")

sm6 <- CreateSeuratObject(counts = sm6, project = "thyroid tumor 3", min.cells = 3, min.features = 200)

P6 <- ScaleData(object =sm6)

sm7 <- Read10X(data.dir = "/home/lq/scRNAseq/GSE184362_PTC/GSM5585108")

sm7 <- CreateSeuratObject(counts = sm7, project = "thyroid paratumor 3", min.cells = 3, min.features = 200)

P7 <- ScaleData(object =sm7)

sm8 <- Read10X(data.dir = "/home/lq/scRNAseq/GSE184362_PTC/GSM5585109")

sm8 <- CreateSeuratObject(counts = sm8, project = "lymph node metastase 2", min.cells = 3, min.features = 200)

P8 <- ScaleData(object =sm8)

sm9 <- Read10X(data.dir = "/home/lq/scRNAseq/GSE184362_PTC/GSM5585110")

sm9 <- CreateSeuratObject(counts = sm9, project = "lymph node metastase 3", min.cells = 3, min.features = 200)

P9 <- ScaleData(object =sm9)

sm10 <- Read10X(data.dir = "/home/lq/scRNAseq/GSE184362_PTC/GSM5585111")

sm10 <- CreateSeuratObject(counts = sm10, project = "subcutaneous metastase 1", min.cells = 3, min.features = 200)

P10 <- ScaleData(object =sm10)

sm11 <- Read10X(data.dir = "/home/lq/scRNAseq/GSE184362_PTC/GSM5585112")

sm11 <- CreateSeuratObject(counts = sm11, project = "thyroid tumor 4", min.cells = 3, min.features = 200)

P11 <- ScaleData(object =sm11)

sm12 <- Read10X(data.dir = "/home/lq/scRNAseq/GSE184362_PTC/GSM5585113")

sm12 <- CreateSeuratObject(counts = sm12, project = "thyroid paratumor 4", min.cells = 3, min.features = 200)

P12 <- ScaleData(object =sm12)

sm13 <- Read10X(data.dir = "/home/lq/scRNAseq/GSE184362_PTC/GSM5585114")

sm13 <- CreateSeuratObject(counts = sm13, project = "lymph node metastase 4", min.cells = 3, min.features = 200)

P13 <- ScaleData(object =sm13)

sm14 <- Read10X(data.dir = "/home/lq/scRNAseq/GSE184362_PTC/GSM5585115")

sm14 <- CreateSeuratObject(counts = sm14, project = "lymph node metastase 5", min.cells = 3, min.features = 200)

P14 <- ScaleData(object =sm14)

sm15 <- Read10X(data.dir = "/home/lq/scRNAseq/GSE184362_PTC/GSM5585116")

sm15 <- CreateSeuratObject(counts = sm15, project = "lymph node metastase 6", min.cells = 3, min.features = 200)

P15 <- ScaleData(object =sm15)

sm16 <- Read10X(data.dir = "/home/lq/scRNAseq/GSE184362_PTC/GSM5585117")

sm16 <- CreateSeuratObject(counts = sm16, project = "thyroid tumor 5", min.cells = 3, min.features = 200)

P16 <- ScaleData(object =sm16)

sm17 <- Read10X(data.dir = "/home/lq/scRNAseq/GSE184362_PTC/GSM5585118")

sm17 <- CreateSeuratObject(counts = sm17, project = "thyroid paratumor 5", min.cells = 3, min.features = 200)

P17 <- ScaleData(object =sm17)

sm18 <- Read10X(data.dir = "/home/lq/scRNAseq/GSE184362_PTC/GSM5585119")

sm18 <- CreateSeuratObject(counts = sm18, project = "thyroid tumor 6", min.cells = 3, min.features = 200)

P18 <- ScaleData(object =sm18)

sm19 <- Read10X(data.dir = "/home/lq/scRNAseq/GSE184362_PTC/GSM5585120")

sm19 <- CreateSeuratObject(counts = sm19, project = "thyroid paratumor 6", min.cells = 3, min.features = 200)

P19 <- ScaleData(object =sm19)

sm20 <- Read10X(data.dir = "/home/lq/scRNAseq/GSE184362_PTC/GSM5585121")

sm20 <- CreateSeuratObject(counts = sm20, project = "thyroid tumor 7", min.cells = 3, min.features = 200)

P20 <- ScaleData(object =sm20)

sm21 <- Read10X(data.dir = "/home/lq/scRNAseq/GSE184362_PTC/GSM5585122")

sm21 <- CreateSeuratObject(counts = sm21, project = "lymph node metastase 7", min.cells = 3, min.features = 200)

P21 <- ScaleData(object =sm21)

sm22 <- Read10X(data.dir = "/home/lq/scRNAseq/GSE184362_PTC/GSM5585123")

sm22 <- CreateSeuratObject(counts = sm22, project = "lymph node metastase 8", min.cells = 3, min.features = 200)

P22 <- ScaleData(object =sm22)

sm23 <- Read10X(data.dir = "/home/lq/scRNAseq/GSE184362_PTC/GSM5585124")

sm23 <- CreateSeuratObject(counts = sm23, project = "subcutaneous metastase 2", min.cells = 3, min.features = 200)

P23 <- ScaleData(object =sm23)

P1@meta.data$sample <- "thyroid tumor 1"

P2@meta.data$sample <- "thyroid paratumor 1"

P3@meta.data$sample <- "thyroid tumor 2"

P4@meta.data$sample <- "thyroid paratumor 2"

P5@meta.data$sample <- "lymph node metastase 1"

P6@meta.data$sample <- "thyroid tumor 3"

P7@meta.data$sample <- "thyroid paratumor 3"

P8@meta.data$sample <- "lymph node metastase 2"

P9@meta.data$sample <- "lymph node metastase 3"

P10@meta.data$sample <- "subcutaneous metastase 1"

P11@meta.data$sample <- "thyroid tumor 4"

P12@meta.data$sample <- "thyroid paratumor 4"

P13@meta.data$sample <- "lymph node metastase 4"

P14@meta.data$sample <- "lymph node metastase 5"

P15@meta.data$sample <- "lymph node metastase 6"

P16@meta.data$sample <- "thyroid tumor 5"

P17@meta.data$sample <- "thyroid paratumor 5"

P18@meta.data$sample <- "thyroid tumor 6"

P19@meta.data$sample <- "thyroid paratumor 6"

P20@meta.data$sample <- "thyroid tumor 7"

P21@meta.data$sample <- "lymph node metastase 7"

P22@meta.data$sample <- "lymph node metastase 8"

P23@meta.data$sample <- "subcutaneous metastase 2"

#######################################

#################################

sm6 <- Read10X(data.dir = "/home/lq/scRNAseq/GSE184362_PTC/GSM5585107")

sm6 <- CreateSeuratObject(counts = sm6, project = "thyroid tumor 1", min.cells = 3, min.features = 200)

P6 <- ScaleData(object =sm6)

sm7 <- Read10X(data.dir = "/home/lq/scRNAseq/GSE184362_PTC/GSM5585108")

sm7 <- CreateSeuratObject(counts = sm7, project = "thyroid paratumor 1", min.cells = 3, min.features = 200)

P7 <- ScaleData(object =sm7)

sm8 <- Read10X(data.dir = "/home/lq/scRNAseq/GSE184362_PTC/GSM5585109")

sm8 <- CreateSeuratObject(counts = sm8, project = "lymph node metastase 1", min.cells = 3, min.features = 200)

P8 <- ScaleData(object =sm8)

sm9 <- Read10X(data.dir = "/home/lq/scRNAseq/GSE184362_PTC/GSM5585110")

sm9 <- CreateSeuratObject(counts = sm9, project = "lymph node metastase 2", min.cells = 3, min.features = 200)

P9 <- ScaleData(object =sm9)

sm10 <- Read10X(data.dir = "/home/lq/scRNAseq/GSE184362_PTC/GSM5585111")

sm10 <- CreateSeuratObject(counts = sm10, project = "subcutaneous metastase 1", min.cells = 3, min.features = 200)

P10 <- ScaleData(object =sm10)

sm11 <- Read10X(data.dir = "/home/lq/scRNAseq/GSE184362_PTC/GSM5585112")

sm11 <- CreateSeuratObject(counts = sm11, project = "thyroid tumor 2", min.cells = 3, min.features = 200)

P11 <- ScaleData(object =sm11)

sm12 <- Read10X(data.dir = "/home/lq/scRNAseq/GSE184362_PTC/GSM5585113")

sm12 <- CreateSeuratObject(counts = sm12, project = "thyroid paratumor 2", min.cells = 3, min.features = 200)

P12 <- ScaleData(object =sm12)

sm13 <- Read10X(data.dir = "/home/lq/scRNAseq/GSE184362_PTC/GSM5585114")

sm13 <- CreateSeuratObject(counts = sm13, project = "lymph node metastase 3", min.cells = 3, min.features = 200)

P13 <- ScaleData(object =sm13)

sm14 <- Read10X(data.dir = "/home/lq/scRNAseq/GSE184362_PTC/GSM5585115")

sm14 <- CreateSeuratObject(counts = sm14, project = "lymph node metastase 4", min.cells = 3, min.features = 200)

P14 <- ScaleData(object =sm14)

sm15 <- Read10X(data.dir = "/home/lq/scRNAseq/GSE184362_PTC/GSM5585116")

sm15 <- CreateSeuratObject(counts = sm15, project = "lymph node metastase 5", min.cells = 3, min.features = 200)

P15 <- ScaleData(object =sm15)

sm16 <- Read10X(data.dir = "/home/lq/scRNAseq/GSE184362_PTC/GSM5585117")

sm16 <- CreateSeuratObject(counts = sm16, project = "thyroid tumor 3", min.cells = 3, min.features = 200)

P16 <- ScaleData(object =sm16)

sm17 <- Read10X(data.dir = "/home/lq/scRNAseq/GSE184362_PTC/GSM5585118")

sm17 <- CreateSeuratObject(counts = sm17, project = "thyroid paratumor 3", min.cells = 3, min.features = 200)

P17 <- ScaleData(object =sm17)

sm18 <- Read10X(data.dir = "/home/lq/scRNAseq/GSE184362_PTC/GSM5585119")

sm18 <- CreateSeuratObject(counts = sm18, project = "thyroid tumor 4", min.cells = 3, min.features = 200)

P18 <- ScaleData(object =sm18)

sm19 <- Read10X(data.dir = "/home/lq/scRNAseq/GSE184362_PTC/GSM5585120")

sm19 <- CreateSeuratObject(counts = sm19, project = "thyroid paratumor 4", min.cells = 3, min.features = 200)

P19 <- ScaleData(object =sm19)

sm20 <- Read10X(data.dir = "/home/lq/scRNAseq/GSE184362_PTC/GSM5585121")

sm20 <- CreateSeuratObject(counts = sm20, project = "thyroid tumor 5", min.cells = 3, min.features = 200)

P20 <- ScaleData(object =sm20)

sm21 <- Read10X(data.dir = "/home/lq/scRNAseq/GSE184362_PTC/GSM5585122")

sm21 <- CreateSeuratObject(counts = sm21, project = "lymph node metastase 6", min.cells = 3, min.features = 200)

P21 <- ScaleData(object =sm21)

sm22 <- Read10X(data.dir = "/home/lq/scRNAseq/GSE184362_PTC/GSM5585123")

sm22 <- CreateSeuratObject(counts = sm22, project = "lymph node metastase 7", min.cells = 3, min.features = 200)

P22 <- ScaleData(object =sm22)

sm23 <- Read10X(data.dir = "/home/lq/scRNAseq/GSE184362_PTC/GSM5585124")

sm23 <- CreateSeuratObject(counts = sm23, project = "subcutaneous metastase 2", min.cells = 3, min.features = 200)

P23 <- ScaleData(object =sm23)

P6@meta.data$sample <- "thyroid tumor 1"

P7@meta.data$sample <- "thyroid paratumor 1"

P8@meta.data$sample <- "lymph node metastase 1"

P9@meta.data$sample <- "lymph node metastase 2"

P10@meta.data$sample <- "subcutaneous metastase 1"

P11@meta.data$sample <- "thyroid tumor 2"

P12@meta.data$sample <- "thyroid paratumor 2"

P13@meta.data$sample <- "lymph node metastase 3"

P14@meta.data$sample <- "lymph node metastase 4"

P15@meta.data$sample <- "lymph node metastase 5"

P16@meta.data$sample <- "thyroid tumor 3"

P17@meta.data$sample <- "thyroid paratumor 3"

P18@meta.data$sample <- "thyroid tumor 4"

P19@meta.data$sample <- "thyroid paratumor 4"

P20@meta.data$sample <- "thyroid tumor 5"

P21@meta.data$sample <- "lymph node metastase 6"

P22@meta.data$sample <- "lymph node metastase 7"

P23@meta.data$sample <- "subcutaneous metastase 2"

#SC.list <- list(P1,P2,P3,P4,P5,P6,P7,P8,P9,P10,P11)

SC.list <- list(P6,P7,P8,P9,P10,P11,P12,P13,P14,P15,P16,P17,P18,P19,P20,P21,P22,P23)

#SC.list <- list(P3,P4,P5,P6)

#SC.list <- list(P1,P2,P3,P4,P7,P10,P11)

#SC.list <- list(P1,P2)

#rm(P1,P2,P3,P4,P5,P6,P7)

#rm(sm1,sm2,sm3,sm4,sm5,sm6,sm7)

#rm(SC.list)

for (i in 1:length(x = SC.list)) {

SC.list[[i]] <- FindVariableFeatures(object = SC.list[[i]],

selection.method = "vst", nfeatures = 1000, verbose = FALSE)

}

SC.anchors <- FindIntegrationAnchors(object.list = SC.list, dims = 1:20)

save(SC.anchors ,file = "SC.Rdata")

#load("SC.Rdata")

# create list of common genes to keep

#to_integrate <- Reduce(intersect, lapply(GCT.anchors@object.list, rownames))

# integrate data and keep full geneset

pbmc <- IntegrateData(anchorset = SC.anchors, dims = 1:20)

pbmc <- ScaleData(object = pbmc, verbose = FALSE)

pbmc <- RunPCA(object = pbmc, npcs = 50, verbose = FALSE)

source("seurat2scanpy.R")

seurat2scanpy(pbmc)

#read.data

sm1 <- Read10X(data.dir = "/home/lq/scRNAseq/GSE184362_PTC/seurat")

sm1 <- CreateSeuratObject(counts = sm1, project = "PTC")

pbmc <- ScaleData(object = sm1, verbose = FALSE)

# 进行QC

# 计算mitochondria基因比例

# Visualize QC metrics as a violin plot

tiff(file="QC_VlnPlot.tiff",res = 300,units = "cm",compression = "lzw", width=24,height=12)

VlnPlot(pbmc, features = c("nFeature_RNA", "nCount_RNA"),pt.size = 0, ncol = 2)

dev.off()

# Normalizing the data

pbmc <- NormalizeData(pbmc)

#Identification of highly variable features

pbmc <- FindVariableFeatures(pbmc, selection.method = "vst", nfeatures = 2000)

# Identify the 10 most highly variable genes

# top10 <- head(VariableFeatures(pbmc), 10)

# plot variable features with and without labels

# plot1 <- VariableFeaturePlot(pbmc)

# plot2 <- LabelPoints(plot = plot1, points = top10, repel = TRUE)

# plot1 + plot2

# Scale the data

all.genes <- rownames(pbmc)

pbmc <- ScaleData(pbmc, features = all.genes)

# Perform linear dimensional reduction

pbmc <- RunPCA(pbmc, features = VariableFeatures(object = pbmc))

#print(pbmc[["pca"]], dims = 1:5, nfeatures = 5)

tiff(file="PCA_VizDimLoadings.tiff",res = 300,units = "cm",compression = "lzw", width=24,height=24)

VizDimLoadings(pbmc, dims = 1:4, reduction = "pca")

dev.off()

tiff(file="PCA_DimHeatmap.tiff展示pc数量对数据分离的帮助",res = 300,units = "cm",compression = "lzw", width=24,height=24)

DimHeatmap(pbmc, dims = 1:15, cells = 500, balanced = TRUE)

dev.off()

tiff(file="PCA_DimPlot.tiff",res = 300,units = "cm",compression = "lzw", width=24,height=24)

DimPlot(pbmc, reduction = "pca")

dev.off()

# NOTE: This process can take a long time for big datasets, comment out for expediency. More

# approximate techniques such as those implemented in ElbowPlot() can be used to reduce

# computation time

##以下酌情运行，比较耗时，可以不运行，直接看ElbowPlot

#pbmc <- JackStraw(pbmc, num.replicate = 100)

#pbmc <- ScoreJackStraw(pbmc, dims = 1:20)

#JackStrawPlot(pbmc,dims = 1:20)

tiff(file="PCA_DimPlot.tiff",res = 300,units = "cm",compression = "lzw", width=10,height=10)

ElbowPlot(pbmc)

dev.off()

#ElbowPlot对主要PC进行排序，“拐弯”处之前的PC均可被选择，这里看到第10个PC处出现拐点，所以后续选择前10个PC进行降维

# Cluster the cells

pbmc <- FindNeighbors(pbmc, dims = 1:20)

##############

pbmc <- FindClusters(pbmc, resolution = 0.2)

#Run non-linear dimensional reduction (UMAP/tSNE)

pbmc <- RunUMAP(pbmc, dims = 1:20)

tiff(file="UMAP_DimPlot_Cluster.tiff",res = 300,units = "cm",compression = "lzw", width=10,height=10)

DimPlot(pbmc, reduction = "umap", label=T)+ NoLegend()

dev.off()

pbmc.markers <- FindAllMarkers(pbmc, only.pos = TRUE, min.pct = 0.25, logfc.threshold = 0.25)

logFCfilter=0.25

adjPvalFilter=0.05

sig.markers=pbmc.markers[(abs(as.numeric(as.vector(pbmc.markers$avg_log2FC)))>logFCfilter),]

write.table(sig.markers,file="06.markers_cluster.xls",sep="\t",row.names=F,quote=F)

saveRDS(pbmc,"pbmc.rds")

##############

# 查看每个cluster的marker gene

library(patchwork)

library(reshape2)

library(RColorBrewer)

library(ggplot2)

library(ggrepel) #用于注释文本

library(magrittr)

pbmc.markers1 <- pbmc.markers

# 查看每个cluster的marker gene

pbmc.markers %>% group_by(cluster) %>% top_n(n = 2, # 前两个

wt = avg_log2FC)

# 查看每个cluster的marker基因数量

table(pbmc.markers$cluster)

# 根据自己计算的marker基因数量确定log2FC的阈值，这里先定为0.5

pbmc.markers <- subset(pbmc.markers, p_val_adj < 0.05 & abs(avg_log2FC) > 0.5)

pbmc.markers$threshold <- as.factor(ifelse(pbmc.markers$avg_log2FC > 0 , 'Up', 'Down'))

dim(pbmc.markers)

table(pbmc.markers$threshold)

pbmc.markers$adj_p_signi <- as.factor(ifelse(pbmc.markers$p_val_adj < 0.01 , 'Highly', 'Lowly'))

pbmc.markers$thr_signi <- paste0(pbmc.markers$threshold, "_", pbmc.markers$adj_p_signi)

pbmc.markers$cluster %<>% as.vector(.) %>% as.numeric(.)

# 保存到文件

write.csv(pbmc.markers, "output_pbmc.markers.csv", quote = F)

#挑选log2FC为top5的基因进行展示

top_up_label <- pbmc.markers %>%

subset(., threshold%in%"Up") %>%

group_by(cluster) %>%

top_n(n = 5, wt = avg_log2FC) %>%

as.data.frame()

top_down_label <- pbmc.markers %>%

subset(., threshold %in% "Down") %>%

group_by(cluster) %>%

top_n(n = -5, wt = avg_log2FC) %>%

as.data.frame()

top_label <- rbind(top_up_label,top_down_label)

top_label$thr_signi %<>%

factor(., levels = c("Up_Highly","Down_Highly","Up_Lowly","Down_Lowly"))

# 保存到文件，便于小白套用格式

write.csv(top_label, "easy_input_label.csv", quote = F)

# 读取修改后的要标注的基因名文件

top_label <- read.csv("easy_input_label.csv")

### 准备绘制暗灰色背景所需数据

background_position <- pbmc.markers %>%

group_by(cluster) %>%

summarise(Min = min(avg_log2FC) - 0.2, Max = max(avg_log2FC) + 0.2) %>%

as.data.frame()

### 准备绘制暗灰色背景所需数据

background_position$cluster %<>% as.vector(.) %>% as.numeric(.)

background_position$start <- background_position$cluster - 0.4

background_position$end <- background_position$cluster + 0.4

### 准备绘制中间区域cluster彩色bar所需数据

cluster_bar_position <- background_position

cluster_bar_position$start <- cluster_bar_position$cluster - 0.5

cluster_bar_position$end <- cluster_bar_position$cluster + 0.5

cluster_bar_position$cluster %<>%

factor(., levels = c(0:max(as.vector(.))))

### 准备绘制中间区域cluster彩色bar所需数据

cluster_bar_position <- background_position

cluster_bar_position$start <- cluster_bar_position$cluster - 0.5

cluster_bar_position$end <- cluster_bar_position$cluster + 0.5

cluster_bar_position$cluster %<>%

factor(., levels = c(0:max(as.vector(.))))

## 设置填充颜色

cols_thr_signi <- c("Up_Highly" = "#d7301f",

"Down_Highly" = "#225ea8",

"Up_Lowly" = "black",

"Down_Lowly" = "black")

cols_cluster <- c("0" = "#35978f",

"1" = "#8dd3c7",

"2" = "#ffffb3",

"3" = "#bebada",

"4" = "#fb8072",

"5" = "#80b1d3",

"6" = "#fdb462",

"7" = "#b3de69",

"8" = "#fccde5",

"9" = "#35978f",

"10" = "#35978f",

"11" = "#8dd3c7",

"12" = "#ffffb3",

"13" = "#bebada",

"14" = "#fb8072",

"15" = "#80b1d3",

"16" = "#fdb462",

"17" = "#b3de69",

"18" = "#fccde5",

"19" = "#35978f",

"20" = "#35978f",

"21" = "#8dd3c7")

p <- ggplot() +

geom_rect(data = background_position, aes(xmin = start, xmax = end, ymin = Min,

ymax = Max),

fill = "#525252", alpha = 0.1) + ###添加灰色背景色

geom_jitter(data = pbmc.markers, aes(x = cluster, y = avg_log2FC, colour = thr_signi),

size = 1,position = position_jitter(seed = 1)) +

scale_color_manual(values = cols_thr_signi) +

scale_x_continuous(limits = c(-0.5, max(pbmc.markers$cluster) + 0.5),

breaks = seq(0, max(pbmc.markers$cluster), 1),

label = seq(0, max(pbmc.markers$cluster),1)) + #修改坐标轴显示刻度

# 根据top_label标注基因名

geom_text_repel(data = top_label, aes(x = cluster, y = avg_log2FC, label = gene),

position = position_jitter(seed = 1), show.legend = F, size = 2.5,

box.padding = unit(0, "lines")) +

geom_rect(data = cluster_bar_position, aes(xmin = start, xmax = end, ymin = -0.4,

ymax = 0.4, fill = cluster), color = "black", alpha = 1, show.legend = F) +

scale_fill_manual(values = cols_cluster) +

labs(x = "Cluster", y = "average log2FC") +

theme_bw()

plot1 <- p + theme(panel.grid.minor = element_blank(), ##去除网格线

panel.grid.major = element_blank(),

axis.text.y = element_text(colour = 'black', size = 14),

axis.text.x = element_text(colour = 'black', size = 14, vjust = 16), #调整x轴坐标,vjust的值按照最终结果稍加调整

panel.border = element_blank(), ## 去掉坐标轴

axis.ticks.x = element_blank(), ## 去掉的坐标刻度线

axis.line.y = element_line(colour = "black")) #添加y轴坐标轴

ggsave(filename = "Marker_gene_pointplot.pdf", plot = plot1, width = 9, height = 6)

##########cellann

library(SingleR)

library(Seurat)

library(tidyverse)

library(BiocNeighbors)

library(celldex)

hpca.se <- HumanPrimaryCellAtlasData()

counts<-pbmc@assays$RNA@counts

clusters<-pbmc@meta.data$seurat_clusters

ann=pbmc@meta.data$orig.ident

singler = SingleR(test = counts, ref = hpca.se,

labels = hpca.se$label.fine,

method = c("single", "cluster"),

clusters = NULL, genes = "de", quantile = 0.8, fine.tune = TRUE, tune.thresh = 0.05, sd.thresh = 1, prune = TRUE,

assay.type.test = "logcounts", assay.type.ref = "logcounts", check.missing = TRUE, BNPARAM = KmknnParam())

table(singler$labels)

pbmc[["SingleR.labels"]] <- singler$labels

pbmc[["bulk_labels"]] <- singler$labels

pbmc@meta.data$SingleR.labels = singler$labels

#################

#write.csv(pbmc@meta.data,file="metadata.csv",row.names=T,quote=F)

meta_data=read.csv("metadata.csv",sep=",",header=T,check.names=F,row.names = 1)

pbmc@meta.data = meta_data

tiff(file="UMAP_DimPlot_bulk_labels.tiff",res = 300,units = "cm",compression = "lzw", width=14,height=10)

DimPlot(pbmc, reduction = "umap", label = F, pt.size = 0.5, group.by = "bulk_labels")

dev.off()

tiff(file="UMAP_DimPlot_sample.tiff",res = 300,units = "cm",compression = "lzw", width=14,height=10)

DimPlot(object = pbmc, reduction = "umap", group.by = "sample", label=F)

dev.off()

tiff(file="UMAP_DimPlot_class.tiff",res = 300,units = "cm",compression = "lzw", width=14,height=10)

DimPlot(object = pbmc, reduction = "umap", group.by = "tissue", label=F)

dev.off()

##############

pA2 <- pA2 <- DimPlot(pbmc, reduction = "umap", label=F,group.by="bulk_labels")

# 自定义颜色

colourCount = length(unique(pbmc@meta.data$bulk_labels))

getPalette = colorRampPalette(brewer.pal(8, "Dark2"))

celltype_colors <- getPalette(colourCount)

pA2 <- pA2 <- DimPlot(pbmc, reduction = "umap", label=F,group.by="bulk_labels",cols=celltype_colors)

tiff(file="UMAP_DimPlot_bulk_labels_2.tiff",res = 300,units = "cm",compression = "lzw", width=14,height=10)

pA2

dev.off()

## 拼图

pA1 <- DimPlot(pbmc, reduction = "umap", label=T,,group.by="seurat_clusters")+ NoLegend()

pA_all <- pA1 + pA2 + plot_layout(ncol = 2, widths=c(1, 1))

tiff(file="UMAP_DimPlot_ALL.tiff",res = 300,units = "cm",compression = "lzw", width=24,height=10)

pA_all

dev.off()

####此处可计算细胞类型的marker用于展示，这里选择用于鉴定细胞类型的marker

Idents(pbmc) <- "bulk_labels"

pbmc.markers <- FindAllMarkers(pbmc, only.pos = TRUE, min.pct = 0.25, logfc.threshold = 0.25)

logFCfilter=0.25

adjPvalFilter=0.05

sig.markers=pbmc.markers[(abs(as.numeric(as.vector(pbmc.markers$avg_log2FC)))>logFCfilter),]

write.table(sig.markers,file="06.markers_cell_type.xls",sep="\t",row.names=F,quote=F)

# 查看top10 marker gene

top10 <- pbmc.markers %>% group_by(cluster) %>% top_n(n = 5, wt = avg_log2FC)

tiff(file="UMAP_DoHeatmap_cell_type.tiff",res = 300,units = "cm",compression = "lzw", width=40,height=20)

DoHeatmap(pbmc, features = top10$gene)

dev.off()

###########

#选择四个基因进行平均表达量展示

#####克利夫兰图

select_gene <- c("TG","C1QB","CD3D","COL1A1")

Idents(pbmc) <- "bulk_labels"

AveExpression <- AverageExpression(pbmc, assays = "RNA", features = select_gene,verbose = TRUE) %>% .$RNA

Ave_df <- melt(AveExpression,id.vars= "Gene")

colnames(Ave_df) <- c("Gene", "Cluster", "Expression")

Ave_df$Group <- paste(Ave_df$Cluster,Ave_df$Gene,sep = "_")

Ave_df$Expression[which(Ave_df$Expression>10)] <- 10 ####设置平均表达量大于10的值为10，放置画出来的bar太高，按照自己数据情况设定

pB1 <- ggplot(Ave_df,aes(x=Gene, y=Expression))+

geom_hline(yintercept = seq(0, 10, 2.5),linetype = 2, color = "lightgray",size=1)+

geom_line()+

geom_segment(aes(x=Gene,xend=Gene,y=0,yend=Expression),color="lightgray",size = 1.5)+

geom_point(size=3,aes(color=Gene))+

scale_color_manual(values=c("#00AFBB", "#E7B800", "#FC4E07", "#41ab5d")) +

theme_bw()+

theme(panel.grid =element_blank()) +

labs(x="",y="Gene exp.")

pB1 <- facet(pB1, facet.by = "Cluster",ncol = length(unique(Ave_df$Cluster)),panel.labs.font = list(size = 12),panel.labs.background = list(fill = "#a6cee3"))

pB1 <- pB1 + scale_y_continuous(position = "right")+ ####用来将y轴移动位置

theme(axis.text.y = element_text(size=12, colour = "black"))+

theme(axis.text.x = element_blank())+ ## 删去所有刻度标签# theme(axis.text.y = element_blank()) ## 设置 axis.text.y 则只删去 Y 轴的刻度标签，X 轴同理。

theme(axis.title.y = element_text(size=12, colour = "black"))+

theme(axis.title.x = element_blank())+

theme(legend.position = "right",

panel.border = element_blank(),## 去掉最外层的正方形边框

axis.ticks.x = element_line(color = NA))

###############

pB2_df <- table(pbmc@meta.data$bulk_labels,pbmc@meta.data$sample) %>% melt()

colnames(pB2_df) <- c("Cluster","Sample","Number")

a = pbmc@meta.data$bulk_labels

bulk_labels = a[!duplicated(a)]

pB2_df$Cluster <- factor(pB2_df$Cluster,levels = bulk_labels)

colourCount1 = length(unique(pbmc@meta.data$sample))

getPalette1 = colorRampPalette(brewer.pal(8, "Dark2"))

sample_color <- getPalette(colourCount1)

pB2 <- ggplot(data = pB2_df, aes(x = Cluster, y = Number, fill = Sample)) +

geom_bar(stat = "identity", width=0.8,position="fill")+

scale_fill_manual(values=sample_color) +

theme_bw()+

theme(panel.grid =element_blank()) +

labs(x="",y="Ratio")+

scale_y_continuous(position = "right")+ ####用来将y轴移动位置

theme(axis.text.y = element_text(size=12, colour = "black"))+

theme(axis.text.x = element_text(size=16, colour = "black",angle = 30,vjust= 0.62))

################

top5 <- pbmc.markers %>% group_by(cluster) %>% top_n(n = 5, wt = avg_log2FC)

heatmap_gene <- as.vector(top5$gene)

#heatmap_gene = heatmap_gene[-5]

heatmap_AveE <- AverageExpression(pbmc, assays = "RNA", features = heatmap_gene,verbose = TRUE,slot = "data") %>% .$RNA

#heatmap_AveE = heatmap_AveE[rowSums(abs(heatmap_AveE)==Inf)==0,,drop=F]

heatmap_AveE[heatmap_AveE==Inf]<-1.122423e+300

#RNA

dim(heatmap_AveE)

gene_num <- c(5,5,5,5,5,5,5)

gaps_row <- cumsum(gene_num)

cluster_num <- c(1,1,1,1,1,1,1)

gaps_col <- cumsum(cluster_num)

celltype = as.vector(pbmc$bulk_labels)

celltype = celltype[!duplicated(celltype)]

bk <- c(seq(-2,-0.1,by=0.01),seq(0,2,by=0.01))

annotation_row <- data.frame(row.names = rownames(heatmap_AveE),

`CellType` = rep(factor(celltype,levels = celltype),gene_num))

annotation_col <- data.frame(row.names = colnames(heatmap_AveE),

`CellType` = rep(factor(celltype,levels = celltype),cluster_num))

annotation_colors = list(`CellType` = celltype_colors)

names(annotation_colors$`CellType`) = celltype

ph3 <- pheatmap(heatmap_AveE,cluster_cols = F,cluster_rows = F,show_colnames=F,show_rownames=T,

border=F,#border_color = "white",

color = c(colorRampPalette(colors = c("#2166ac","#f7fbff"))(length(bk)/2),

colorRampPalette(colors = c("#f7fbff","#b2182b"))(length(bk)/2)),

breaks=bk,scale="row",legend_breaks=seq(-2,2,2),

gaps_row = gaps_row,gaps_col = gaps_col,

annotation_row = annotation_row,annotation_col = annotation_col,

annotation_colors = annotation_colors,

annotation_names_row = F,annotation_names_col = T,fontsize_row=8)

require(ggplotify)

pB3 = as.ggplot(ph3) ### 将pheatmap对象转为ggplot对象，便于后续拼图

pB_1_2 <- pB1 + pB2 + plot_layout(ncol = 1, heights = c(1, 2))

## 尝试将热图也拼在一起，发现并不能完全对，后期用AI拼效果更好

## 尝试将热图也拼在一起，发现并不能完全对

#pB_all <- pB1 + pB2 + pB3 + plot_layout(ncol = 1, heights = c(1,2,3))

tiff(file="UMAP_DoHeatmap_all.tiff",res = 300,units = "cm",compression = "lzw", width=30,height=20)

pB_1_2

dev.off()

tiff(file="UMAP_DoHeatmap_top5.tiff",res = 300,units = "cm",compression = "lzw", width=15,height=20)

pB3

dev.off()

##############

data_plotC <- table(pbmc@meta.data$sample, pbmc@meta.data$bulk_labels) %>% melt()

colnames(data_plotC) <- c("Sample", "CellType","Number")

pC1 <- ggplot(data = data_plotC, aes(x = Sample, y = Number, fill = CellType)) +

geom_bar(stat = "identity", width=0.8,aes(group=CellType),position="stack")+

scale_fill_manual(values=celltype_colors) +

theme_bw()+

theme(panel.grid =element_blank()) +

labs(x="",y="Average number")+

theme(axis.text = element_text(size=12, colour = "black"))+

theme(axis.title.y = element_text(size=12, colour = "black"))+

theme(panel.border = element_rect(size = 1, linetype = "solid", colour = "black"))+

theme(axis.text.x = element_text(angle = 45,hjust = 0.8, vjust = 0.6))

pC2 <- ggplot(data = data_plotC, aes(x = Sample, y = Number, fill = CellType)) +

geom_bar(stat = "identity", width=0.8,aes(group=CellType),position="fill")+

scale_fill_manual(values=celltype_colors) +

theme_bw()+

theme(panel.grid =element_blank()) +

labs(x="",y="Cell proportion")+

scale_y_continuous()+ ####用来将y轴移动位置

theme(axis.text = element_text(size=12, colour = "black"))+

theme(axis.title.y = element_text(size=12, colour = "black"))+

theme(panel.border = element_rect(size = 1, linetype = "solid", colour = "black"))+

theme(axis.text.x = element_text(angle = 45,hjust = 0.8, vjust = 0.6)) #让横轴上的标签倾斜45度

pC <- pC1 + pC2 + plot_layout(ncol = 2, widths = c(1,1),guides = 'collect')

tiff(file="UMAP_cell_number_proportion.tiff",res = 300,units = "cm",compression = "lzw", width=30,height=15)

pC

dev.off()

##########

setwd("/home/lq/scRNAseq/GSE184362_PTC")

sig.markers = read.table("06.markers_cell_type.xls",sep="\t",header=T,check.names=F)

cluster10Marker=sig.markers[,7]

cluster10Marker=as.matrix(cluster10Marker)

cluster10Marker=cluster10Marker[!duplicated(cluster10Marker),]

cluster10Marker=as.matrix(cluster10Marker)

picDir="UMAP_DEG_features"

dir.create(picDir)

setwd(picDir)

#?VlnPlot

for(i in 1:nrow(cluster10Marker)){

pdf(file=paste(cluster10Marker[i,],"Violin.pdf"),width=10,height=6)

p=VlnPlot(object = pbmc, features = cluster10Marker[i,],pt.size = 0 )

print(p)

dev.off()

}

#?FeaturePlot

for(i in 1:nrow(cluster10Marker)){

tiff(file=paste(cluster10Marker[i,],"FeaturePlot.tiff"),res = 300,units = "cm",width=8,height=8)

p=FeaturePlot(object = pbmc, features = cluster10Marker[i,])

print(p)

dev.off()

}

##########

setwd("/home/lq/scRNAseq/GSE184362_PTC")

########ccell cycle

cc.genes

## Not run:

# pbmc_small doesn't have any cell-cycle genes

# To run CellCycleScoring, please use a dataset with cell-cycle genes

# An example is available at http://satijalab.org/seurat/cell_cycle_vignette.html

pbmc <- CellCycleScoring(

object = pbmc,

g2m.features = cc.genes$g2m.genes,

s.features = cc.genes$s.genes,replace=T

)

head(x = pbmc@meta.data)

tiff(file="UMAP_cell_cycle_VlnPlot.tiff",res = 300,units = "cm",compression = "lzw", width=15,height=8)

VlnPlot(pbmc, features = c("G2M.Score","S.Score"), ncol = 2, pt.size = 0)#+scale_color_npg()

dev.off()

umapem<-pbmc@reductions$umap@cell.embeddings

metada = pbmc@meta.data

dim(umapem);dim(metada)

ccdata<-data.frame(umapem,metada)

head(ccdata)

library(ggplot2)

plot<-ggplot(ccdata, aes(UMAP_1, UMAP_2,label=Phase))+geom_point(aes(colour = factor(Phase)),size=0.3)+

#plot<-plot+scale_colour_manual(values=c("#CC33FF","Peru","#660000","#660099","#990033","black","red", "#666600", "green","#6699CC","#339900","#0000FF","#FFFF00","#808080"))+

labs("@yunlai",x = "", y="")

plot=plot+

theme_bw()+theme(panel.grid=element_blank(),legend.title=element_blank(),legend.text = element_text(color="black", size = 10, face = "bold"))

plot<-plot+guides(colour = guide_legend(override.aes = list(size=5))) +theme(plot.title = element_text(hjust = 0.5))

tiff(file="UMAP_cell_cycle.tiff",res = 300,units = "cm",compression = "lzw", width=10,height=8)

plot

dev.off()

######sub_cluster

#先执行不同resolution 下的分群

library(Seurat)

library(ggalluvial)

library(tidyverse)

pbmc_small <- FindClusters(

object = pbmc,

resolution = c(seq(.4,1.6,.2))

)

head(pbmc_small@meta.data)

pbmc_small$RNA_snn_res.0.25

tiff(file="UMAP_resolution.tiff",res = 300,units = "cm",compression = "lzw", width=15,height=15)

ggplot(data = pbmc_small@meta.data,

aes(axis1 = RNA_snn_res.0.4, axis2 = RNA_snn_res.0.6, axis3 = RNA_snn_res.0.8,axis4 = RNA_snn_res.1,

axis5 = RNA_snn_res.1.2,axis6 = RNA_snn_res.1.4,axis7 = RNA_snn_res.1.6)) +

scale_x_discrete(limits = c(paste0("RNA_snn_res",seq(.4,1.6,.2))), expand = c(.01, .05)) +

geom_alluvium(aes(fill = RNA_snn_res.1.6)) +

geom_stratum() + #geom_text(stat = "stratum", infer.label = TRUE) +

#coord_polar()+

theme(axis.text.x = element_text(angle = 90, hjust = 1))+

ggtitle("cell number in each cluster")

dev.off()

########

library(clustree)

tiff(file="UMAP_clustree.tiff",res = 300,units = "cm",compression = "lzw", width=20,height=25)

clustree::clustree(pbmc_small@meta.data, prefix = "RNA_snn_res.")

dev.off()

###############italk

#iTALK为R包，可通过以下方式安装

#devtools::install_github("Coolgenome/iTALK", build_vignettes = TRUE)

#我们以Seurat的对象文件为例展示iTALK的数据载入

library(iTALK)

library(Seurat)

library(Matrix)

library(dplyr)

# iTALK 要求的矩阵: 行为细胞，列为基因

data1 = pbmc@assays$RNA@counts

gene = pbmc@assays$RNA@var.features

data1= data1[gene,]

data1[1:5,1:5]

data1=as.matrix(data1)

gene=rownames(data1)

iTalk_data <- as.data.frame(t(data1))

# iTALK 要求包含cell_type列，我的细胞分群存储在seurat_cluster

iTalk_data$cell_type <- pbmc@meta.data$bulk_labels

# iTALK 要求包含compare_group列（多样本），表示每个细胞的生物学分组/样本，我的细胞分组存放在Group

iTalk_data$compare_group <- pbmc@meta.data$sample

unique(iTalk_data$cell_type)

# "cd56_nk" "cd14_monocytes" "b_cells" "cytotoxic_t" "regulatory_t" "memory_t" "naive_t"

unique(iTalk_data$compare_group)

# "group1" "group2" "group3"

###########

my10colors <-c('#E5D2DD', '#53A85F', '#F1BB72', '#F3B1A0', '#D6E7A3', '#57C3F3', '#476D87', '#E95C59', '#E59CC4', '#AB3282')

highly_exprs_genes <- rawParse(iTalk_data, top_genes=500, stats="mean")

# 通讯类型

comm_list<-c('growth factor','other','cytokine','checkpoint')

cell_types <- unique(iTalk_data$cell_type)

cell_col <- structure(my10colors[1:length(cell_types)], names=cell_types)

iTalk_res <- NULL

for(comm_type in comm_list){

res_cat <- FindLR(highly_exprs_genes, datatype='mean count', comm_type=comm_type)

iTalk_res <- rbind(iTalk_res, res_cat)

}

iTalk_res <- iTalk_res[order(iTalk_res$cell_from_mean_exprs*iTalk_res$cell_to_mean_exprs,decreasing=T),][1:100,]

dim(iTalk_res)

tiff(file="UMAP_iTALK_network.tiff",res = 300,units = "cm",compression = "lzw", width=15,height=15)

NetView(iTalk_res,col=cell_col,vertex.label.cex=1,arrow.width=1,edge.max.width=5)

dev.off()

tiff(file="UMAP_iTALK_LRPlot.tiff",res = 300,units = "cm",compression = "lzw", width=15,height=15)

LRPlot(iTalk_res[1:100,],datatype='mean count',cell_col=cell_col,link.arr.lwd=iTalk_res$cell_from_mean_exprs[1:100],link.arr.width=iTalk_res$cell_to_mean_exprs[1:100])

dev.off()

iTalk_res1=cbind(rownames(iTalk_res),iTalk_res)

write.table(iTalk_res1,file="UMAP_iTalk_res.txt",sep="\t",quote=F,row.names = F,col.names = T)

saveRDS(pbmc,"pbmc.rds")

###############

iTalk_res_final <- read.table("UMAP_iTalk_res_Final.txt", header=T, sep="\t",row.names = 1,check.names = F)

head(iTalk_res)

head(iTalk_res_final)

tiff(file="UMAP_iTALK_LRPlot_final.tiff",res = 300,units = "cm",compression = "lzw", width=15,height=15)

LRPlot(iTalk_res_final,datatype='mean count',cell_col=cell_col,link.arr.lwd=iTalk_res_final$cell_from_mean_exprs,link.arr.width=iTalk_res_final$cell_to_mean_exprs)

dev.off()

tiff(file="UMAP_iTALK_network_final.tiff",res = 300,units = "cm",compression = "lzw", width=15,height=15)

NetView(iTalk_res,col=cell_col,vertex.label.cex=1,arrow.width=1,edge.max.width=5)

dev.off()

#############################################################################################################

#############sub

Idents(pbmc) <- "bulk_labels"

subcluster = c("Malignant cells")

sub <- subset(pbmc, idents = subcluster)

df.data <- GetAssayData(object = sub, slot = "data")

#PCA

pbmc = sub

picDir="sub"

dir.create(picDir)

setwd(picDir)

# Normalizing the data

pbmc <- NormalizeData(pbmc)

#Identification of highly variable features

pbmc <- FindVariableFeatures(pbmc, selection.method = "vst", nfeatures = 2000)

# Identify the 10 most highly variable genes

# top10 <- head(VariableFeatures(pbmc), 10)

# plot variable features with and without labels

# plot1 <- VariableFeaturePlot(pbmc)

# plot2 <- LabelPoints(plot = plot1, points = top10, repel = TRUE)

# plot1 + plot2

# Scale the data

all.genes <- rownames(pbmc)

pbmc <- ScaleData(pbmc, features = all.genes)

# Perform linear dimensional reduction

pbmc <- RunPCA(pbmc, features = VariableFeatures(object = pbmc))

#print(pbmc[["pca"]], dims = 1:5, nfeatures = 5)

tiff(file="PCA_VizDimLoadings.tiff",res = 300,units = "cm",compression = "lzw", width=24,height=24)

VizDimLoadings(pbmc, dims = 1:4, reduction = "pca")

dev.off()

tiff(file="PCA_DimHeatmap.tiff",res = 300,units = "cm",compression = "lzw", width=24,height=24)

DimHeatmap(pbmc, dims = 1:15, cells = 500, balanced = TRUE)

dev.off()

tiff(file="PCA_DimPlot.tiff",res = 300,units = "cm",compression = "lzw", width=24,height=24)

DimPlot(pbmc, reduction = "pca")

dev.off()

# NOTE: This process can take a long time for big datasets, comment out for expediency. More

# approximate techniques such as those implemented in ElbowPlot() can be used to reduce

# computation time

##以下酌情运行，比较耗时，可以不运行，直接看ElbowPlot

#pbmc <- JackStraw(pbmc, num.replicate = 100)

#pbmc <- ScoreJackStraw(pbmc, dims = 1:20)

#JackStrawPlot(pbmc,dims = 1:20)

tiff(file="PCA_DimPlot.tiff",res = 300,units = "cm",compression = "lzw", width=10,height=10)

ElbowPlot(pbmc)

dev.off()

#ElbowPlot对主要PC进行排序，“拐弯”处之前的PC均可被选择，这里看到第10个PC处出现拐点，所以后续选择前10个PC进行降维

# Cluster the cells

pbmc <- FindNeighbors(pbmc, dims = 1:20)

##############

pbmc <- FindClusters(pbmc, resolution = 0.05)

#Run non-linear dimensional reduction (UMAP/tSNE)

pbmc <- RunUMAP(pbmc, dims = 1:20)

tiff(file="UMAP_DimPlot_Cluster.tiff",res = 300,units = "cm",compression = "lzw", width=10,height=10)

DimPlot(pbmc, reduction = "umap", label=T)+ NoLegend()

dev.off()

pbmc.markers <- FindAllMarkers(pbmc, min.pct = 0.25, logfc.threshold = 0.25)

logFCfilter=0.5

adjPvalFilter=0.05

sig.markers=pbmc.markers[(abs(as.numeric(as.vector(pbmc.markers$avg_log2FC)))>logFCfilter),]

write.table(sig.markers,file="06.markers_cluster.xls",sep="\t",row.names=F,quote=F)

##############

# 查看每个cluster的marker gene

library(patchwork)

library(reshape2)

library(RColorBrewer)

library(ggplot2)

library(ggrepel) #用于注释文本

library(magrittr)

pbmc.markers1 <- pbmc.markers

# 查看每个cluster的marker gene

pbmc.markers %>% group_by(cluster) %>% top_n(n = 2, # 前两个

wt = avg_log2FC)

# 查看每个cluster的marker基因数量

table(pbmc.markers$cluster)

# 根据自己计算的marker基因数量确定log2FC的阈值，这里先定为0.5

pbmc.markers <- subset(pbmc.markers, p_val_adj < 0.05 & abs(avg_log2FC) > 0.5)

pbmc.markers$threshold <- as.factor(ifelse(pbmc.markers$avg_log2FC > 0 , 'Up', 'Down'))

dim(pbmc.markers)

table(pbmc.markers$threshold)

pbmc.markers$adj_p_signi <- as.factor(ifelse(pbmc.markers$p_val_adj < 0.01 , 'Highly', 'Lowly'))

pbmc.markers$thr_signi <- paste0(pbmc.markers$threshold, "_", pbmc.markers$adj_p_signi)

pbmc.markers$cluster %<>% as.vector(.) %>% as.numeric(.)

# 保存到文件

write.csv(pbmc.markers, "output_pbmc.markers.csv", quote = F)

#挑选log2FC为top5的基因进行展示

top_up_label <- pbmc.markers %>%

subset(., threshold%in%"Up") %>%

group_by(cluster) %>%

top_n(n = 5, wt = avg_log2FC) %>%

as.data.frame()

top_down_label <- pbmc.markers %>%

subset(., threshold %in% "Down") %>%

group_by(cluster) %>%

top_n(n = -5, wt = avg_log2FC) %>%

as.data.frame()

top_label <- rbind(top_up_label,top_down_label)

top_label$thr_signi %<>%

factor(., levels = c("Up_Highly","Down_Highly","Up_Lowly","Down_Lowly"))

# 保存到文件，便于小白套用格式

write.csv(top_label, "easy_input_label.csv", quote = F)

# 读取修改后的要标注的基因名文件

top_label <- read.csv("easy_input_label.csv")

### 准备绘制暗灰色背景所需数据

background_position <- pbmc.markers %>%

group_by(cluster) %>%

summarise(Min = min(avg_log2FC) - 0.2, Max = max(avg_log2FC) + 0.2) %>%

as.data.frame()

### 准备绘制暗灰色背景所需数据

background_position$cluster %<>% as.vector(.) %>% as.numeric(.)

background_position$start <- background_position$cluster - 0.4

background_position$end <- background_position$cluster + 0.4

### 准备绘制中间区域cluster彩色bar所需数据

cluster_bar_position <- background_position

cluster_bar_position$start <- cluster_bar_position$cluster - 0.5

cluster_bar_position$end <- cluster_bar_position$cluster + 0.5

cluster_bar_position$cluster %<>%

factor(., levels = c(0:max(as.vector(.))))

### 准备绘制中间区域cluster彩色bar所需数据

cluster_bar_position <- background_position

cluster_bar_position$start <- cluster_bar_position$cluster - 0.5

cluster_bar_position$end <- cluster_bar_position$cluster + 0.5

cluster_bar_position$cluster %<>%

factor(., levels = c(0:max(as.vector(.))))

## 设置填充颜色

cols_thr_signi <- c("Up_Highly" = "#d7301f",

"Down_Highly" = "#225ea8",

"Up_Lowly" = "black",

"Down_Lowly" = "black")

cols_cluster <- c("0" = "#35978f",

"1" = "#8dd3c7",

"2" = "#ffffb3",

"3" = "#bebada",

"4" = "#fb8072",

"5" = "#80b1d3",

"6" = "#fdb462",

"7" = "#b3de69",

"8" = "#fccde5",

"9" = "#35978f",

"10" = "#35978f",

"11" = "#8dd3c7",

"12" = "#ffffb3",

"13" = "#bebada",

"14" = "#fb8072",

"15" = "#80b1d3",

"16" = "#fdb462",

"17" = "#b3de69",

"18" = "#fccde5",

"19" = "#35978f",

"20" = "#35978f",

"21" = "#8dd3c7")

p <- ggplot() +

geom_rect(data = background_position, aes(xmin = start, xmax = end, ymin = Min,

ymax = Max),

fill = "#525252", alpha = 0.1) + ###添加灰色背景色

geom_jitter(data = pbmc.markers, aes(x = cluster, y = avg_log2FC, colour = thr_signi),

size = 1,position = position_jitter(seed = 1)) +

scale_color_manual(values = cols_thr_signi) +

scale_x_continuous(limits = c(-0.5, max(pbmc.markers$cluster) + 0.5),

breaks = seq(0, max(pbmc.markers$cluster), 1),

label = seq(0, max(pbmc.markers$cluster),1)) + #修改坐标轴显示刻度

# 根据top_label标注基因名

geom_text_repel(data = top_label, aes(x = cluster, y = avg_log2FC, label = gene),

position = position_jitter(seed = 1), show.legend = F, size = 2.5,

box.padding = unit(0, "lines")) +

geom_rect(data = cluster_bar_position, aes(xmin = start, xmax = end, ymin = -0.4,

ymax = 0.4, fill = cluster), color = "black", alpha = 1, show.legend = F) +

scale_fill_manual(values = cols_cluster) +

labs(x = "Cluster", y = "average log2FC") +

theme_bw()

plot1 <- p + theme(panel.grid.minor = element_blank(), ##去除网格线

panel.grid.major = element_blank(),

axis.text.y = element_text(colour = 'black', size = 14),

axis.text.x = element_text(colour = 'black', size = 14, vjust = 71), #调整x轴坐标,vjust的值按照最终结果稍加调整

panel.border = element_blank(), ## 去掉坐标轴

axis.ticks.x = element_blank(), ## 去掉的坐标刻度线

axis.line.y = element_line(colour = "black")) #添加y轴坐标轴

ggsave(filename = "Marker_gene_pointplot.pdf", plot = plot1, width = 9, height = 6)

#################

#write.csv(pbmc@meta.data,file="metadata.csv",row.names=T,quote=F)

meta_data=read.csv("metadata.csv",sep=",",header=T,check.names=F,row.names = 1)

pbmc@meta.data = meta_data

tiff(file="UMAP_DimPlot_bulk_labels.tiff",res = 300,units = "cm",compression = "lzw", width=14,height=10)

DimPlot(pbmc, reduction = "umap", label = F, pt.size = 0.5, group.by = "bulk_labels")

dev.off()

tiff(file="UMAP_DimPlot_sample.tiff",res = 300,units = "cm",compression = "lzw", width=14,height=10)

DimPlot(object = pbmc, reduction = "umap", group.by = "sample", label=F)

dev.off()

#tiff(file="UMAP_DimPlot_class.tiff",res = 300,units = "cm",compression = "lzw", width=14,height=10)

#DimPlot(object = pbmc, reduction = "umap", group.by = "tissue", label=F)

#dev.off()

##############

pA2 <- pA2 <- DimPlot(pbmc, reduction = "umap", label=F,group.by="bulk_labels")

# 自定义颜色

colourCount = length(unique(pbmc@meta.data$bulk_labels))

getPalette = colorRampPalette(brewer.pal(8, "Dark2"))

celltype_colors <- getPalette(colourCount)

pA2 <- pA2 <- DimPlot(pbmc, reduction = "umap", label=F,group.by="bulk_labels",cols=celltype_colors)

tiff(file="UMAP_DimPlot_bulk_labels_2.tiff",res = 300,units = "cm",compression = "lzw", width=14,height=10)

pA2

dev.off()

## 拼图

pA1 <- DimPlot(pbmc, reduction = "umap", label=T)+ NoLegend()

pA_all <- pA1 + pA2 + plot_layout(ncol = 2, widths=c(1, 1))

tiff(file="UMAP_DimPlot_ALL.tiff",res = 300,units = "cm",compression = "lzw", width=24,height=10)

pA_all

dev.off()

saveRDS(pbmc,"pbmc.rds")

####此处可计算细胞类型的marker用于展示，这里选择用于鉴定细胞类型的marker

Idents(pbmc) <- "bulk_labels"

pbmc.markers <- FindAllMarkers(pbmc, only.pos = TRUE, min.pct = 0.25, logfc.threshold = 0.25)

logFCfilter=0.25

adjPvalFilter=0.05

sig.markers=pbmc.markers[(abs(as.numeric(as.vector(pbmc.markers$avg_log2FC)))>logFCfilter),]

write.table(sig.markers,file="06.markers_cell_type.xls",sep="\t",row.names=F,quote=F)

# 查看top10 marker gene

top10 <- pbmc.markers %>% group_by(cluster) %>% top_n(n = 5, wt = avg_log2FC)

tiff(file="UMAP_DoHeatmap_cell_type.tiff",res = 300,units = "cm",compression = "lzw", width=40,height=20)

DoHeatmap(pbmc, features = top10$gene)

dev.off()

###########

#选择四个基因进行平均表达量展示

#####克利夫兰图 Figure 2B

select_gene <- c("CD44","CD24","PROM1","MKI67")

Idents(pbmc) <- "bulk_labels"

AveExpression <- AverageExpression(pbmc, assays = "RNA", features = select_gene,verbose = TRUE) %>% .$RNA

Ave_df <- melt(AveExpression,id.vars= "Gene")

colnames(Ave_df) <- c("Gene", "Cluster", "Expression")

Ave_df$Group <- paste(Ave_df$Cluster,Ave_df$Gene,sep = "_")

Ave_df$Expression[which(Ave_df$Expression>10)] <- 10 ####设置平均表达量大于10的值为10，放置画出来的bar太高，按照自己数据情况设定

pB1 <- ggplot(Ave_df,aes(x=Gene, y=Expression))+

geom_hline(yintercept = seq(0, 10, 2.5),linetype = 2, color = "lightgray",size=1)+

geom_line()+

geom_segment(aes(x=Gene,xend=Gene,y=0,yend=Expression),color="lightgray",size = 1.5)+

geom_point(size=3,aes(color=Gene))+

scale_color_manual(values=c("#00AFBB", "#E7B800", "#FC4E07", "#41ab5d")) +

theme_bw()+

theme(panel.grid =element_blank()) +

labs(x="",y="Gene exp.")

pB1 <- facet(pB1, facet.by = "Cluster",ncol = length(unique(Ave_df$Cluster)),panel.labs.font = list(size = 12),panel.labs.background = list(fill = "#a6cee3"))

pB1 <- pB1 + scale_y_continuous(position = "right")+ ####用来将y轴移动位置

theme(axis.text.y = element_text(size=12, colour = "black"))+

theme(axis.text.x = element_blank())+ ## 删去所有刻度标签# theme(axis.text.y = element_blank()) ## 设置 axis.text.y 则只删去 Y 轴的刻度标签，X 轴同理。

theme(axis.title.y = element_text(size=12, colour = "black"))+

theme(axis.title.x = element_blank())+

theme(legend.position = "right",

panel.border = element_blank(),## 去掉最外层的正方形边框

axis.ticks.x = element_line(color = NA))

###############

pB2_df <- table(pbmc@meta.data$bulk_labels,pbmc@meta.data$sample) %>% melt()

colnames(pB2_df) <- c("Cluster","Sample","Number")

a = pbmc@meta.data$bulk_labels

bulk_labels = a[!duplicated(a)]

pB2_df$Cluster <- factor(pB2_df$Cluster,levels = bulk_labels)

colourCount1 = length(unique(pbmc@meta.data$sample))

getPalette1 = colorRampPalette(brewer.pal(8, "Dark2"))

sample_color <- getPalette(colourCount1)

pB2 <- ggplot(data = pB2_df, aes(x = Cluster, y = Number, fill = Sample)) +

geom_bar(stat = "identity", width=0.8,position="fill")+

scale_fill_manual(values=sample_color) +

theme_bw()+

theme(panel.grid =element_blank()) +

labs(x="",y="Ratio")+

scale_y_continuous(position = "right")+ ####用来将y轴移动位置

theme(axis.text.y = element_text(size=12, colour = "black"))+

theme(axis.text.x = element_text(size=16, colour = "black",angle = 30,vjust= 0.62))

################

top5 <- pbmc.markers %>% group_by(cluster) %>% top_n(n = 5, wt = avg_log2FC)

heatmap_gene <- as.vector(top5$gene)

#heatmap_gene = heatmap_gene[-5]

heatmap_AveE <- AverageExpression(pbmc, assays = "RNA", features = heatmap_gene,verbose = TRUE,slot = "data") %>% .$RNA

#heatmap_AveE = heatmap_AveE[rowSums(abs(heatmap_AveE)==Inf)==0,,drop=F]

#heatmap_AveE[heatmap_AveE==Inf]<-1.122423e+300

#RNA

dim(heatmap_AveE)

gene_num <- c(5,5,5,5,5,5,5)

gaps_row <- cumsum(gene_num)

cluster_num <- c(1,1,1,1,1,1,1)

gaps_col <- cumsum(cluster_num)

celltype = as.vector(pbmc$bulk_labels)

celltype = celltype[!duplicated(celltype)]

bk <- c(seq(-2,-0.1,by=0.01),seq(0,2,by=0.01))

annotation_row <- data.frame(row.names = rownames(heatmap_AveE),

`CellType` = rep(factor(celltype,levels = celltype),gene_num))

annotation_col <- data.frame(row.names = colnames(heatmap_AveE),

`CellType` = rep(factor(celltype,levels = celltype),cluster_num))

annotation_colors = list(`CellType` = celltype_colors)

names(annotation_colors$`CellType`) = celltype

ph3 <- pheatmap(heatmap_AveE,cluster_cols = F,cluster_rows = F,show_colnames=F,show_rownames=T,

border=F,#border_color = "white",

color = c(colorRampPalette(colors = c("#2166ac","#f7fbff"))(length(bk)/2),

colorRampPalette(colors = c("#f7fbff","#b2182b"))(length(bk)/2)),

breaks=bk,scale="row",legend_breaks=seq(-2,2,2),

gaps_row = gaps_row,gaps_col = gaps_col,

annotation_row = annotation_row,annotation_col = annotation_col,

annotation_colors = annotation_colors,

annotation_names_row = F,annotation_names_col = T,fontsize_row=8)

require(ggplotify)

pB3 = as.ggplot(ph3) ### 将pheatmap对象转为ggplot对象，便于后续拼图

pB_1_2 <- pB1 + pB2 + plot_layout(ncol = 1, heights = c(1, 2))

## 尝试将热图也拼在一起，发现并不能完全对，后期用AI拼效果更好

## 尝试将热图也拼在一起，发现并不能完全对

#pB_all <- pB1 + pB2 + pB3 + plot_layout(ncol = 1, heights = c(1,2,3))

tiff(file="UMAP_DoHeatmap_all.tiff",res = 300,units = "cm",compression = "lzw", width=35,height=20)

pB_1_2

dev.off()

tiff(file="UMAP_DoHeatmap_top5.tiff",res = 300,units = "cm",compression = "lzw", width=15,height=20)

pB3

dev.off()

##############

data_plotC <- table(pbmc@meta.data$sample, pbmc@meta.data$bulk_labels) %>% melt()

colnames(data_plotC) <- c("Sample", "CellType","Number")

pC1 <- ggplot(data = data_plotC, aes(x = Sample, y = Number, fill = CellType)) +

geom_bar(stat = "identity", width=0.8,aes(group=CellType),position="stack")+

scale_fill_manual(values=celltype_colors) +

theme_bw()+

theme(panel.grid =element_blank()) +

labs(x="",y="Average number")+

theme(axis.text = element_text(size=12, colour = "black"))+

theme(axis.title.y = element_text(size=12, colour = "black"))+

theme(panel.border = element_rect(size = 1, linetype = "solid", colour = "black"))+

theme(axis.text.x = element_text(angle = 45,hjust = 0.8, vjust = 0.6))

pC2 <- ggplot(data = data_plotC, aes(x = Sample, y = Number, fill = CellType)) +

geom_bar(stat = "identity", width=0.8,aes(group=CellType),position="fill")+

scale_fill_manual(values=celltype_colors) +

theme_bw()+

theme(panel.grid =element_blank()) +

labs(x="",y="Cell proportion")+

scale_y_continuous()+ ####用来将y轴移动位置

theme(axis.text = element_text(size=12, colour = "black"))+

theme(axis.title.y = element_text(size=12, colour = "black"))+

theme(panel.border = element_rect(size = 1, linetype = "solid", colour = "black"))+

theme(axis.text.x = element_text(angle = 45,hjust = 0.8, vjust = 0.6)) #让横轴上的标签倾斜45度

pC <- pC1 + pC2 + plot_layout(ncol = 2, widths = c(1,1),guides = 'collect')

tiff(file="UMAP_cell_number_proportion.tiff",res = 300,units = "cm",compression = "lzw", width=40,height=15)

pC

dev.off()

##########

setwd("/home/lq/scRNAseq/GSE184362_PTC/sub")

#pbmc = readRDS("pbmc.rds")

sig.markers = read.table("06.markers_cell_type.xls",sep="\t",header=T,check.names=F)

logFCfilter=1.5

adjPvalFilter=0.05

#sig.markers=sig.markers[(abs(as.numeric(as.vector(sig.markers$avg_log2FC)))>logFCfilter),]

cluster10Marker=sig.markers[,7]

cluster10Marker=as.matrix(cluster10Marker)

cluster10Marker=cluster10Marker[!duplicated(cluster10Marker),]

cluster10Marker=as.matrix(cluster10Marker)

picDir="UMAP_DEG_features"

dir.create(picDir)

setwd(picDir)

#?VlnPlot

for(i in 1:nrow(cluster10Marker)){

pdf(file=paste(cluster10Marker[i,],"Violin.pdf"),width=10,height=6)

p=VlnPlot(object = pbmc, features = cluster10Marker[i,],pt.size = 0 )

print(p)

dev.off()

}

#?FeaturePlot

for(i in 1:nrow(cluster10Marker)){

tiff(file=paste(cluster10Marker[i,],"FeaturePlot.tiff"),res = 300,units = "cm",width=8,height=8)

p=FeaturePlot(object = pbmc, features = cluster10Marker[i,])

print(p)

dev.off()

}到这里是整个Figure 1+Figure S2即全样本细胞单细胞测序的代码

##########

setwd("/home/lq/scRNAseq/GSE184362_PTC/sub")

########ccell cycle

cc.genes

## Not run:

# pbmc_small doesn't have any cell-cycle genes

# To run CellCycleScoring, please use a dataset with cell-cycle genes

# An example is available at http://satijalab.org/seurat/cell_cycle_vignette.html

pbmc <- CellCycleScoring(

object = pbmc,

g2m.features = cc.genes$g2m.genes,

s.features = cc.genes$s.genes,replace=T

)

head(x = pbmc@meta.data)

tiff(file="UMAP_cell_cycle_VlnPlot.tiff",res = 300,units = "cm",compression = "lzw", width=15,height=8)

VlnPlot(pbmc, features = c("G2M.Score","S.Score"), ncol = 2, pt.size = 0)#+scale_color_npg()

dev.off()

umapem<-pbmc@reductions$umap@cell.embeddings

metada = pbmc@meta.data

dim(umapem);dim(metada)

ccdata<-data.frame(umapem,metada)

head(ccdata)

library(ggplot2)

plot<-ggplot(ccdata, aes(UMAP_1, UMAP_2,label=Phase))+geom_point(aes(colour = factor(Phase)),size=0.3)+

#plot<-plot+scale_colour_manual(values=c("#CC33FF","Peru","#660000","#660099","#990033","black","red", "#666600", "green","#6699CC","#339900","#0000FF","#FFFF00","#808080"))+

labs("@yunlai",x = "", y="")

plot=plot+

theme_bw()+theme(panel.grid=element_blank(),legend.title=element_blank(),legend.text = element_text(color="black", size = 10, face = "bold"))

plot<-plot+guides(colour = guide_legend(override.aes = list(size=5))) +theme(plot.title = element_text(hjust = 0.5))

tiff(file="UMAP_cell_cycle.tiff",res = 300,units = "cm",compression = "lzw", width=10,height=8)

plot

dev.off()

######sub_cluster

#先执行不同resolution 下的分群

library(Seurat)

library(ggalluvial)

library(tidyverse)

pbmc_small <- FindClusters(

object = pbmc,

resolution = c(seq(.4,1.6,.2))

)

head(pbmc_small@meta.data)

pbmc_small$RNA_snn_res.0.25

tiff(file="UMAP_resolution.tiff",res = 300,units = "cm",compression = "lzw", width=15,height=15)

ggplot(data = pbmc_small@meta.data,

aes(axis1 = RNA_snn_res.0.4, axis2 = RNA_snn_res.0.6, axis3 = RNA_snn_res.0.8,axis4 = RNA_snn_res.1,

axis5 = RNA_snn_res.1.2,axis6 = RNA_snn_res.1.4,axis7 = RNA_snn_res.1.6)) +

scale_x_discrete(limits = c(paste0("RNA_snn_res",seq(.4,1.6,.2))), expand = c(.01, .05)) +

geom_alluvium(aes(fill = RNA_snn_res.1.6)) +

geom_stratum() + #geom_text(stat = "stratum", infer.label = TRUE) +

#coord_polar()+

theme(axis.text.x = element_text(angle = 90, hjust = 1))+

ggtitle("cell number in each cluster")

dev.off()

########

library(clustree)

tiff(file="UMAP_clustree.tiff",res = 300,units = "cm",compression = "lzw", width=20,height=25)

clustree::clustree(pbmc_small@meta.data, prefix = "RNA_snn_res.")

dev.off()

###############italk

#iTALK为R包，可通过以下方式安装

#devtools::install_github("Coolgenome/iTALK", build_vignettes = TRUE)

#我们以Seurat的对象文件为例展示iTALK的数据载入

library(iTALK)

library(Seurat)

library(Matrix)

library(dplyr)

# iTALK 要求的矩阵: 行为细胞，列为基因

data1 = pbmc@assays$RNA@counts

gene = pbmc@assays$RNA@var.features

data1= data1[gene,]

data1[1:5,1:5]

data1=as.matrix(data1)

gene=rownames(data1)

iTalk_data <- as.data.frame(t(data1))

# iTALK 要求包含cell_type列，我的细胞分群存储在seurat_cluster

iTalk_data$cell_type <- pbmc@meta.data$bulk_labels

# iTALK 要求包含compare_group列（多样本），表示每个细胞的生物学分组/样本，我的细胞分组存放在Group

iTalk_data$compare_group <- pbmc@meta.data$sample

unique(iTalk_data$cell_type)

# "cd56_nk" "cd14_monocytes" "b_cells" "cytotoxic_t" "regulatory_t" "memory_t" "naive_t"

unique(iTalk_data$compare_group)

# "group1" "group2" "group3"

###########

colourCount = length(unique(pbmc@meta.data$bulk_labels))

getPalette = colorRampPalette(brewer.pal(8, "Dark2"))

my10colors <- getPalette(colourCount)

highly_exprs_genes <- rawParse(iTalk_data, top_genes=500, stats="mean")

# 通讯类型

comm_list<-c('growth factor','other','cytokine','checkpoint')

cell_types <- unique(iTalk_data$cell_type)

cell_col <- structure(my10colors[1:length(cell_types)], names=cell_types)

iTalk_res <- NULL

for(comm_type in comm_list){

res_cat <- FindLR(highly_exprs_genes, datatype='mean count', comm_type=comm_type)

iTalk_res <- rbind(iTalk_res, res_cat)

}

iTalk_res <- iTalk_res[order(iTalk_res$cell_from_mean_exprs*iTalk_res$cell_to_mean_exprs,decreasing=T),][1:100,]

dim(iTalk_res)

tiff(file="UMAP_iTALK_network.tiff",res = 300,units = "cm",compression = "lzw", width=15,height=15)

NetView(iTalk_res,col=cell_col,vertex.label.cex=1,arrow.width=1,edge.max.width=5)

dev.off()

tiff(file="UMAP_iTALK_LRPlot.tiff",res = 300,units = "cm",compression = "lzw", width=15,height=15)

LRPlot(iTalk_res[1:100,],datatype='mean count',cell_col=cell_col,link.arr.lwd=iTalk_res$cell_from_mean_exprs[1:100],link.arr.width=iTalk_res$cell_to_mean_exprs[1:100])

dev.off()

iTalk_res1=cbind(rownames(iTalk_res),iTalk_res)

write.table(iTalk_res1,file="UMAP_iTalk_res.txt",sep="\t",quote=F,row.names = F,col.names = T)

############## moncle2

library(monoc num_clustersle)

library(DDRTree)

library(pheatmap)

library(Seurat)

library(cowplot)

library(ggplot2)

library(dplyr)

library(devtools)

library(roxygen2)

library(RColorBrewer)

setwd("/home/lq/scRNAseq/GSE184362_PTC/sub")

#pbmc = readRDS("pbmc.rds")

rt = pbmc@assays$RNA@counts

gene = pbmc@assays$RNA@var.features

#data <- as(as.matrix(rt[gene,]), 'sparseMatrix')

data <- as(as.matrix(rt), 'sparseMatrix')

pd <- new('AnnotatedDataFrame', data = pbmc@meta.data)

fData <- data.frame(gene_short_name = row.names(data), row.names = row.names(data))

fd <- new('AnnotatedDataFrame', data = fData)

#Construct monocle cds

monocle_cds <- newCellDataSet(data,

phenoData = pd,

featureData = fd,

lowerDetectionLimit = 0.5,

expressionFamily = negbinomial.size())

gbm <-monocle_cds

# this table will be useful for switching gene IDs to symbols

head(fData(gbm))

# rename gene symbol column

my_feat <- fData(gbm)

my_feat$id <-my_feat$gene_short_name

my_cds <- newCellDataSet(

exprs(gbm),

phenoData = new("AnnotatedDataFrame",

data = pData(gbm)),

featureData = new("AnnotatedDataFrame",

data = my_feat),

lowerDetectionLimit = 0.5,

expressionFamily = negbinomial.size())

my_cds <- estimateSizeFactors(my_cds) #--normlization ----

my_cds <- estimateDispersions(my_cds) #--calculate variance----

my_cds <- detectGenes(my_cds, min_expr = 0.1) #--filter lower quality cells

summary(fData(my_cds)$num_cells_expressed)

HSMM_myo<-my_cds

fData(HSMM_myo)$use_for_ordering <-fData(HSMM_myo)$num_cells_expressed > 0.05 * ncol(HSMM_myo)

HSMM_myo <- reduceDimension(HSMM_myo,max_components=2,norm_method = 'log',num_dim = 3,reduction_method = 'tSNE',verbose = T,check_duplicates = FALSE)

number <-5

HSMM_myo <- clusterCells(HSMM_myo, verbose = F,num_clusters = number) #输入2_s的

prefix= "UMAP_monocle2"

graph <- paste(prefix,'cell_clusters.tiff',sep='_')

colourCount = length(unique(pbmc@meta.data$seurat_clusters))

getPalette = colorRampPalette(brewer.pal(8, "Dark2"))

celltype_colors <- getPalette(colourCount)

tiff(graph,res = 300,units = "cm",compression = "lzw", width=10,height=15)

plot_cell_clusters(HSMM_myo, color_by = 'as.factor(Cluster)')+

scale_colour_manual(values=celltype_colors) +

theme(axis.ticks = element_blank(),axis.text = element_blank()) + #去掉刻度线

guides(color = guide_legend(ncol = 3, title = "Cluster",override.aes=list(size=3)))+#图例分2列，不要图例的题目

theme(legend.text=element_text(size=8))

dev.off()

# 拟时间分析

GM_state <- function(cds){

if (length(unique(pData(cds)$State)) > 1){

T0_counts <- table(pData(cds)$State, pData(cds)$Hours)[,"0"]

return(as.numeric(names(T0_counts)[which(T0_counts == max(T0_counts))]))

} else {

return (1)

}

}

#过滤基因

HSMM_expressed_genes <-row.names(subset(fData(HSMM_myo),num_cells_expressed >= 10))

#鉴定差异基因

clustering_DEG_genes <-differentialGeneTest(HSMM_myo[HSMM_expressed_genes,],fullModelFormulaStr = '~seurat_clusters') #这一步特别耗时

HSMM_ordering_genes <-row.names(clustering_DEG_genes)[order(clustering_DEG_genes$qval)][1:1000]

HSMM_myo <-setOrderingFilter(HSMM_myo,ordering_genes = HSMM_ordering_genes)

print ("Starting reduceDimension..............................")

# DDRTree 降维

HSMM_myo <-reduceDimension(HSMM_myo,max_components=2,norm_method = 'log',num_dim = 3, method = 'DDRTree', check_duplicates = FALSE)

print ("Finishing reduceDimension..............................")

saveRDS(HSMM_myo,"DDRTree.rds")

#HSMM_myo = readRDS("DDRTree.rds")

# 细胞排序

#memory.size()

#memory.limit()

#devtools::install_github("krlmlr/ulimit")

#ulimit::memory_limit(200000)

HSMM_myo <-orderCells(HSMM_myo)

#HSMM_myo <-orderCells(HSMM_myo, root_state = GM_state(HSMM_myo))

Cluster_num<-ceiling(length(unique(pData(HSMM_myo)$State)))

pData(HSMM_myo)$seurat_clusters <- as.factor(pData(HSMM_myo)$seurat_clusters)

colourCount = length(unique(HSMM_myo$State))

getPalette = colorRampPalette(brewer.pal(8, "Dark2"))

celltype_colors <- getPalette(colourCount)

graph <- paste(prefix,'cell_trajectory_State.tiff',sep='_')

tiff(graph,res = 300,units = "cm",compression = "lzw", width=10,height=10)

plot_cell_trajectory(HSMM_myo, color_by = "State",cell_size = 0.75)+

scale_colour_manual(values=celltype_colors) +

theme(axis.ticks = element_blank(),axis.text = element_blank()) + #去掉刻度线

guides(color = guide_legend(ncol = 3, title = "State",override.aes=list(size=3)))+#图例分2列，不要图例的题目

theme(legend.text=element_text(size=8))

dev.off()

graph <- paste(prefix,'cell_trajectory_State_all.tiff',sep='_')

tiff(graph,res = 300,units = "cm",compression = "lzw", width=8,height=15)

plot_cell_trajectory(HSMM_myo, color_by = "State",cell_size = 0.75)+

scale_colour_manual(values=celltype_colors) +

theme(axis.ticks = element_blank(),axis.text = element_blank()) + #去掉刻度线

guides(color = guide_legend(ncol = 3, title = "State",override.aes=list(size=3)))+#图例分2列，不要图例的题目

theme(legend.text=element_text(size=8))+

facet_wrap(~State, nrow = Cluster_num)

dev.off()

graph <- paste(prefix,'cell_trajectory_Pseudotime.tiff',sep='_')

tiff(graph,res = 300,units = "cm",compression = "lzw", width=10,height=10)

plot_cell_trajectory(HSMM_myo, color_by = "Pseudotime",cell_size = 0.75)

dev.off()

colourCount = length(unique(pbmc@meta.data$seurat_clusters))

getPalette = colorRampPalette(brewer.pal(8, "Dark2"))

celltype_colors <- getPalette(colourCount)

graph <- paste(prefix,'cell_trajectory_seurat_clusters.tiff',sep='_')

tiff(graph,res = 300,units = "cm",compression = "lzw", width=10,height=12)

plot_cell_trajectory(HSMM_myo, color_by = "seurat_clusters",cell_size = 0.75)+

scale_colour_manual(values=celltype_colors) +

theme(axis.ticks = element_blank(),axis.text = element_blank()) + #去掉刻度线

guides(color = guide_legend(ncol = 3, title = "Cluster",override.aes=list(size=3)))+#图例分2列，不要图例的题目

theme(legend.text=element_text(size=8))

dev.off()

colourCount = length(unique(pbmc@meta.data$bulk_labels))

getPalette = colorRampPalette(brewer.pal(8, "Dark2"))

celltype_colors <- getPalette(colourCount)

graph <- paste(prefix,'cell_trajectory_Cell_type.tiff',sep='_')

tiff(graph,res = 300,units = "cm",compression = "lzw", width=10,height=12)

plot_cell_trajectory(HSMM_myo, color_by = "bulk_labels",cell_size = 0.75)+

scale_colour_manual(values=celltype_colors) +

theme(axis.ticks = element_blank(),axis.text = element_blank()) + #去掉刻度线

guides(color = guide_legend(ncol = 3, title = "Cell type",override.aes=list(size=3)))+#图例分2列，不要图例的题目

theme(legend.text=element_text(size=8))

dev.off()

graph <- paste(prefix,'cell_trajectory_Cell_type_all.tiff',sep='_')

tiff(graph,res = 300,units = "cm",compression = "lzw", width=20,height=20)

plot_cell_trajectory(HSMM_myo, color_by = "bulk_labels",cell_size = 0.75)+

scale_colour_manual(values=celltype_colors) +

theme(axis.ticks = element_blank(),axis.text = element_blank()) + #去掉刻度线

guides(color = guide_legend(ncol = 3, title = "Cell type",override.aes=list(size=3)))+#图例分2列，不要图例的题目

theme(legend.text=element_text(size=8))+facet_wrap(~bulk_labels, nrow =Cluster_num)

dev.off()

################

HSMM_expressed_genes <-row.names(subset(fData(HSMM_myo),num_cells_expressed >= 10))

HSMM_filtered <- HSMM_myo[HSMM_expressed_genes,]

my_pseudotime_de <- differentialGeneTest(HSMM_myo,fullModelFormulaStr = "~sm.ns(Pseudotime)")

my_pseudotime_de %>% arrange(qval) %>% head()

## save the top genes

my_pseudotime_de %>% arrange(qval) %>% head() %>% select(id) -> my_pseudotime_gene

my_genes <- as.character(my_pseudotime_gene$id)

cds_subset <- HSMM_filtered[my_genes,]

colourCount = length(unique(HSMM_myo$State))

getPalette = colorRampPalette(brewer.pal(3, "Dark2"))

celltype_colors <- getPalette(colourCount)

graph <- paste(prefix,'genes_in_pseudotime_top_6.tiff',sep='_')

tiff(graph,res = 300,units = "cm",compression = "lzw", width=10,height=15)

plot_genes_in_pseudotime(cds_subset, color_by = "State")+

scale_colour_manual(values=celltype_colors) +

theme(axis.ticks = element_blank(),axis.text = element_blank()) + #去掉刻度线

guides(color = guide_legend(ncol = 1, title = "State",override.aes=list(size=3)))+#图例分2列，不要图例的题目

theme(legend.text=element_text(size=8))+theme(text = element_text(size=22))

dev.off()

colourCount = length(unique(HSMM_myo$bulk_labels))

getPalette = colorRampPalette(brewer.pal(8, "Dark2"))

celltype_colors <- getPalette(colourCount)

graph <- paste(prefix,'genes_in_Cell_type_top_6.tiff',sep='_')

tiff(graph,res = 300,units = "cm",compression = "lzw", width=10,height=15)

plot_genes_in_pseudotime(cds_subset, color_by = "bulk_labels")+

scale_colour_manual(values=celltype_colors) +

theme(axis.ticks = element_blank(),axis.text = element_blank()) + #去掉刻度线

guides(color = guide_legend(ncol = 1, title = "Cell type",override.aes=list(size=3)))+#图例分2列，不要图例的题目

theme(legend.text=element_text(size=8))+theme(text = element_text(size=22))

dev.off()

colourCount = length(unique(HSMM_myo$sample))

getPalette = colorRampPalette(brewer.pal(8, "Dark2"))

celltype_colors <- getPalette(colourCount)

graph <- paste(prefix,'genes_in_sample_top_6.tiff',sep='_')

tiff(graph,res = 300,units = "cm",compression = "lzw", width=10,height=15)

plot_genes_in_pseudotime(cds_subset, color_by = "sample")+

scale_colour_manual(values=celltype_colors) +

theme(axis.ticks = element_blank(),axis.text = element_blank()) + #去掉刻度线

guides(color = guide_legend(ncol = 1, title = "Sample",override.aes=list(size=3)))+#图例分2列，不要图例的题目

theme(legend.text=element_text(size=8))+theme(text = element_text(size=22)) Medullary Thyroid Cancer: 60 Years of Gradual Understanding

dev.off()

#plot_pseudotime_heatmap

#cluster the top 50 genes that vary as a function of pseudotime

my_pseudotime_de %>% arrange(qval) %>% head(100) %>% select(id) -> gene_to_cluster

gene_to_cluster <- gene_to_cluster$id

colourCount = length(unique(HSMM_myo$State))

getPalette = colorRampPalette(brewer.pal(3, "Dark2"))

celltype_colors <- getPalette(colourCount)

graph <- paste(prefix,'pseudotime_heatmap_top_100.tiff',sep='_')

tiff(graph,res = 300,units = "cm",compression = "lzw", width=10,height=18)

my_pseudotime_cluster <-plot_pseudotime_heatmap(HSMM_myo[gene_to_cluster,],

show_rownames = TRUE,return_heatmap = TRUE,num_clusters = Cluster_num)

# scale_colour_manual(values=celltype_colors)

dev.off()

#############绘制拟时间分支热图

## hierarchical clustering was used to cluster the genes

# we can cut the dendrogram to form the same 3 clusters as plot_pseudotime_heatmap

my_cluster <- cutree(my_pseudotime_cluster$tree_row, Cluster_num)

# genes in cluster 1

my_pseudotime_de[names(my_cluster[my_cluster == 1]),"gene_short_name"]

# genes in cluster 2

my_pseudotime_de[names(my_cluster[my_cluster == 2]),"gene_short_name"]

# genes in cluster 3

my_pseudotime_de[names(my_cluster[my_cluster == 3]),"gene_short_name"]

##The BEAM() function takes a CellDataSet that has been ordered with orderCells() and a branch point in the trajectory. A table of genes is returned with significance values that indicate whether genes have expression patterns that are branch dependent.

BEAM_res <- BEAM(HSMM_myo, branch_point = 1)

BEAM_res <- BEAM_res[order(BEAM_res$qval),]

BEAM_res <- BEAM_res[,c("gene_short_name", "pval", "qval")]

# check out the results

head(BEAM_res)

graph <- paste(prefix,'branched_heatmap.tiff',sep='_')

tiff(graph,res = 300,units = "cm",compression = "lzw", width=10,height=18)

my_branched_heatmap <- plot_genes_branched_heatmap(HSMM_myo[row.names(subset(BEAM_res, qval < 1e-3)),],branch_point = 1,num_clusters = Cluster_num,use_gene_short_name = TRUE,show_rownames = FALSE,return_heatmap = TRUE) ###show_rownames = TRUE

dev.off()

###########

state_DEG_genes <-differentialGeneTest(HSMM_myo[HSMM_expressed_genes,],fullModelFormulaStr = '~State') #这一步特别耗时

write.csv(state_DEG_genes,paste(prefix,'diff_state_gene.csv',sep="_"),quote=F)

##############

my_row <- my_branched_heatmap$annotation_row

my_row <- data.frame(cluster = my_row$Cluster,gene = row.names(my_row),stringsAsFactors = FALSE)

write.csv(my_row,paste(prefix,'diff_state_gene_cluster.csv',sep="_"),quote=F)

################

gene = pbmc@assays$RNA@var.features

state_DEG_genes_final= state_DEG_genes[gene,]

write.csv(state_DEG_genes_final,paste(prefix,'state_DEG_genes_final.csv',sep="_"),quote=F)

###################

diff_state_gene_cluster_final= my_row[which(as.vector(my_row[,2])%in%gene),]

write.csv(diff_state_gene_cluster_final,paste(prefix,'diff_state_gene_cluster_final.csv',sep="_"),quote=F)

###########

final = intersect(as.vector(diff_state_gene_cluster_final[,2]),rownames(state_DEG_genes_final))

x1 = state_DEG_genes_final[final,]

x2= diff_state_gene_cluster_final[which(as.vector(diff_state_gene_cluster_final[,2])%in%final),]

monocle_final = cbind(x1,x2)

write.csv(monocle_final,paste(prefix,'final_results.csv',sep="_"),quote=F)

#############################GSVA

library(Seurat)

library(ComplexHeatmap)

library(GSVA)

library(GSEABase)

library(limma)

library(ggplot2)

# 导入gmt文件，这里以MsigDB中的Hallmark为例

# MsigDB链接(https://www.gsea-msigdb.org/gsea/msigdb/)

# 下载文件时，注意选择对应的gene格式吗，一般10X的结果，都是gene symbol格式的。

load("/home/lq/scRNAseq/hallmark.gs.RData")

genesets <- gs

### 1. 找到感兴趣的细胞组别，两者间进行比较

# 这里以cluster6和cluster8为例

subcluster = unique(pbmc@meta.data$bulk_labels)

sub <- subset(pbmc, idents = subcluster)

df.data <- GetAssayData(object = sub, slot = "data")

# 将细胞所属的group信息保存起来

df.group <- data.frame(umi = names(Idents(sub)),

cluster = as.character(sub@meta.data$bulk_labels),

stringsAsFactors = F)

# 查看一下结果是什么样子的

head(df.group)

### 2. 做gsva

# 因为是normalized后的数据，所以使用默认参数就可以了。根据自己的电脑cpu来调整parallel.sz

gsvascore <- gsva(data.matrix(df.data), genesets)

gsvascore[1:5, 1:5]

# 先用热图展示一下hallmarker中全部的50个geneset得到的结果

tiff(file="UMAP_GSVA_heatmap.tiff",res = 300,units = "cm",compression = "lzw", width=30,height=20)

ha.t <- HeatmapAnnotation(Cluster = df.group$cluster)

Heatmap(as.matrix(gsvascore),

show_column_names = F,

cluster_rows = T,

cluster_columns = T,

top_annotation = ha.t,

column_split = df.group$cluster,

row_names_gp = gpar(fontsize = 8),

row_names_max_width = max_text_width(rownames(gsvascore),

gp = gpar(fontsize = 8)))

dev.off()
