# Supplementary figures and images for: Single-cell RNA sequencing and multi-omics analysis of prognosis-related staging in papillary thyroid cancer
Source: Cancer Immunol Immunother. 2025 Jul 12;74(8):267. doi: 10.1007/s00262-025-04101-4 (PMC12255609; doi:10.1007/s00262-025-04101-4)

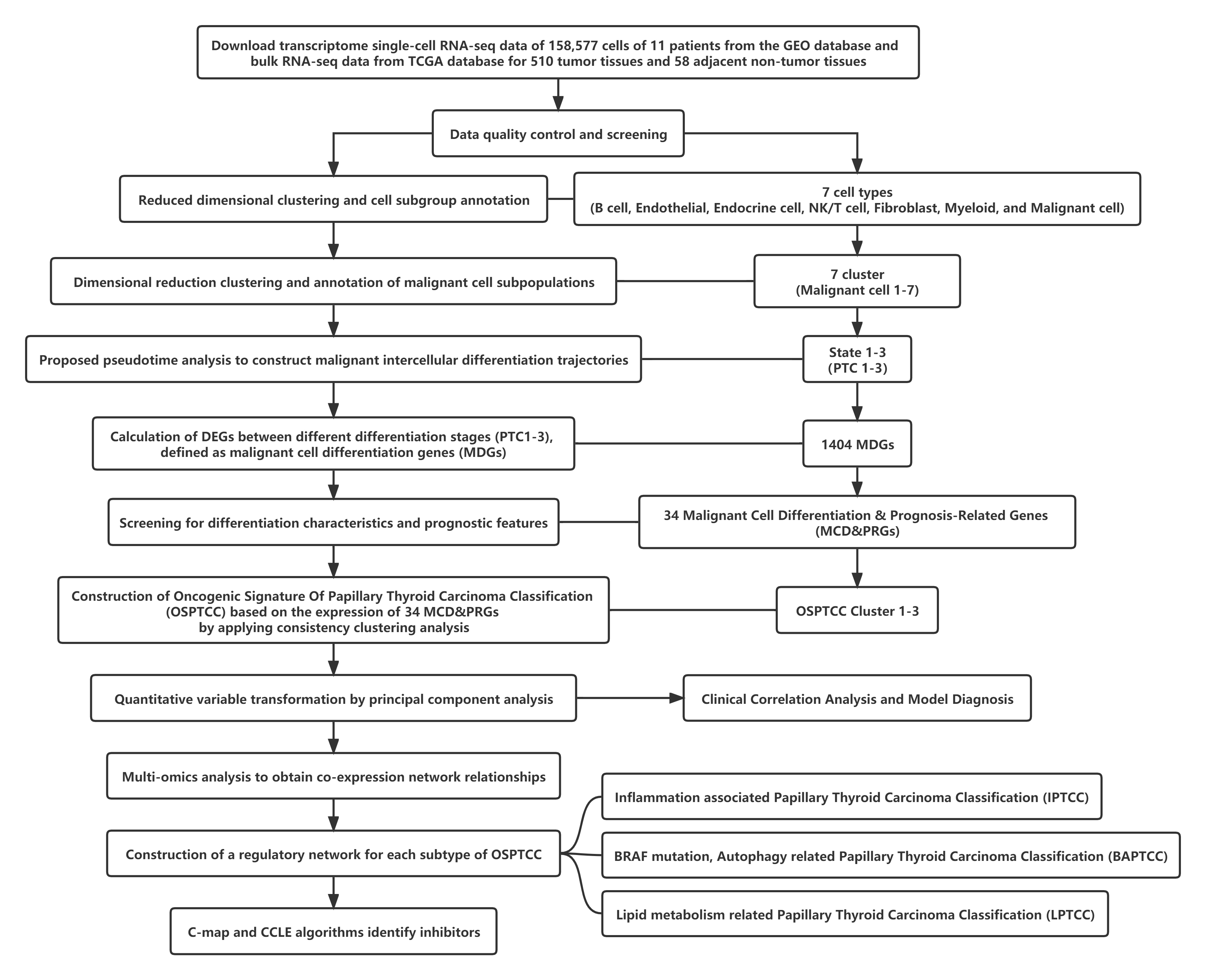

Supplement: Supplementary file 2 — Supplementary file2 (ZIP 46990 KB) [file 262_2025_4101_MOESM2_ESM.zip › New folder2/Figure S1.jpg]

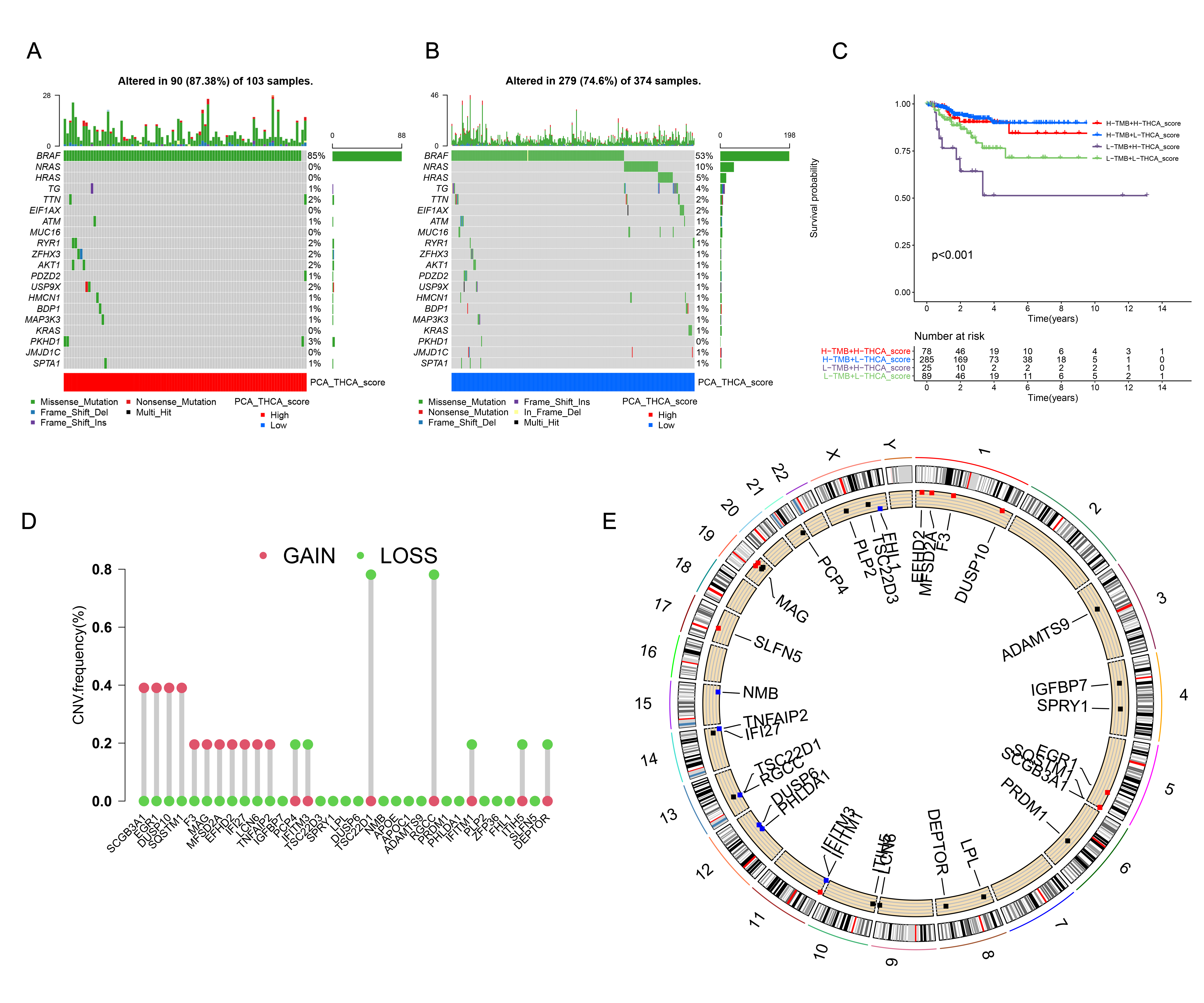

Supplement: Supplementary file 2 — Supplementary file2 (ZIP 46990 KB) [file 262_2025_4101_MOESM2_ESM.zip › New folder2/Figure S5.jpg]
