# Supplementary material for: Single-cell RNA sequencing and multi-omics analysis of prognosis-related staging in papillary thyroid cancer
Source: Cancer Immunol Immunother. 2025 Jul 12;74(8):267. doi: 10.1007/s00262-025-04101-4 (PMC12255609; doi:10.1007/s00262-025-04101-4)
Supplement: Supplementary file 3 — Supplementary file3 (ZIP 77 KB) [file 262_2025_4101_MOESM3_ESM.zip › Table S1.docx]

Supplementary Table S1: Clinico-Pathological Characteristics of the 8 Non-PTC Cases Included in the TCGA Analysis Cohort

| TCGA Case ID | Gender | Race | Ethnicity | Age at Diagnosis | Primary Diagnosis |
| --- | --- | --- | --- | --- | --- |
| TCGA-BJ-A192 | female | white | not reported | 54 years 155 days | Oxyphilic adenocarcinoma |
| TCGA-IM-A41Y | female | white | hispanic or latino | 42 years 354 days | Nonencapsulated sclerosing carcinoma |
| TCGA-BJ-A0ZF | female | white | not hispanic or latino | 54 years 255 days | Follicular carcinoma, minimally invasive |
| TCGA-IM-A4EB | male | white | hispanic or latino | 37 years 121 days | Nonencapsulated sclerosing carcinoma |
| TCGA-EM-A2CM | female | not reported | not reported | 62 years 81 days | Carcinoma, NOS |
| TCGA-DJ-A3VJ | male | white | not hispanic or latino | 22 years 306 days | Nonencapsulated sclerosing carcinoma |
| TCGA-DJ-A4UQ | male | asian | not hispanic or latino | 60 years 182 days | Nonencapsulated sclerosing carcinoma |
| TCGA-BJ-A0Z2 | male | black or african american | not hispanic or latino | 57 years 189 days | Follicular adenocarcinoma, NOS |

NOS: Not Otherwise Specified.
