# Supplementary material for: Single-cell RNA sequencing and multi-omics analysis of prognosis-related staging in papillary thyroid cancer
Source: Cancer Immunol Immunother. 2025 Jul 12;74(8):267. doi: 10.1007/s00262-025-04101-4 (PMC12255609; doi:10.1007/s00262-025-04101-4)
Supplement: Supplementary file 3 — Supplementary file3 (ZIP 77 KB) [file 262_2025_4101_MOESM3_ESM.zip › Table S4.docx]

**Table S4.** Information of Primer Sequences

| Gene | Sequences（5’→3’) |
| --- | --- |
| APOC1 | F: TCCAGTGCCTTGGATAAGCTG |
|  | R: GGCTGATGAGTTCCCGAGC |
| APOE | F: GTTGCTGGTCACATTCCTGG |
|  | R: GCAGGTAATCCCAAAAGCGAC |
| DEPTOR | F: GCGGAGGCGAAGACTGATG |
|  | R: GGCTCACTGACATAAAGCTGGTA |
| SQSTM1 | F: GACTACGACTTGTGTAGCGTC |
|  | R: AGTGTCCGTGTTTCACCTTCC |
| β-actin | F: CATGTACGTTGCTATCCAGGC |
|  | R: CTCCTTAATGTCACGCACGAT |
