# Supplementary material for: Single-cell RNA sequencing and multi-omics analysis of prognosis-related staging in papillary thyroid cancer
Source: Cancer Immunol Immunother. 2025 Jul 12;74(8):267. doi: 10.1007/s00262-025-04101-4 (PMC12255609; doi:10.1007/s00262-025-04101-4)
Supplement: Supplementary file 3 — Supplementary file3 (ZIP 77 KB) [file 262_2025_4101_MOESM3_ESM.zip › Table S5.docx]

**Table S5.** Information of Tissue Samples collected from 11 PTC patients

| **Patient ID** | **Primary Tumor** | **Paratumor** | **Lymph Node Metastasis** | **Subcutaneous Metastasis** | **Treatment History** |
| --- | --- | --- | --- | --- | --- |
| Patient 1 | Yes | Yes | No | No | No treatment |
| Patient 2 | Yes | Yes | Yes (Left) | No | No treatment |
| Patient 3 | Yes | Yes | Yes (Left & Right) | No | No treatment |
| Patient 4 | No | No | No | Yes | Iodine ablation & TSH suppression |
| Patient 5 | Yes | Yes | Yes (Right) | No | No treatment |
| Patient 6 | No | No | Yes (Right) | No | Total thyroidectomy & TSH suppression |
| Patient 7 | No | No | Yes (Right) | No | Hemi-thyroidectomy & TSH suppression |
| Patient 8 | Yes | Yes | No | No | No treatment |
| Patient 9 | Yes | Yes | No | No | No treatment |
| Patient 10 | Yes | No | Yes (Right) | No | No treatment |
| Patient 11 | No | No | No | Yes | Total thyroidectomy, three iodine ablations & TSH suppression |

This table summarizes tissue samples collected from 11 PTC patients, representing the complete spectrum of papillary thyroid carcinoma progression from treatment-naïve primary tumors to RAI-refractory distant metastases.
